# Supplementary material for: Loss of MBNL1 induces RNA misprocessing in the thymus and peripheral blood
Source: Nat Commun. 2020 Apr 24;11:2022. doi: 10.1038/s41467-020-15962-x (PMC7181699; doi:10.1038/s41467-020-15962-x)
Supplement: Supplementary file 1 — Supplementary Information [file 41467_2020_15962_MOESM1_ESM.pdf]

Supplementary Information

Loss of MBNL1 induces RNA mis-processing in the thymus and peripheral blood

Ł. J. Sznajder et al.

Table of Contents

Supplementary Figure 1.	Gene expression changes in 129-*Mbnl1* KO thymus	p. 3-4
Supplementary Figure 2.	Thymic pathology in 129-*Mbnl1* KO	p. 5-6
Supplementary Figure 3.	Genome browser views of selected mouse alternative splicing events	p. 7-12
Supplementary Figure 4.	MBNL1 directly regulates Lef1 E6	p. 13-14
Supplementary Figure 5.	Alternative splicing changes in *Mbnl1* KO splenocytes and PBMCs	p. 15
Supplementary Figure 6.	Alternative splicing changes in DM2 PBMCs	p. 16-17
Supplementary Figure 7.	Gene expression changes in DM2 whole blood	p. 18
Supplementary Figure 8.	Genome browser views of selected human alternative splicing events	p. 19-31
Supplementary Figure 9.	Alternative splicing changes in DM2 whole blood	p. 32-33
Supplementary Figure 10.	Blood DM2 alternative splicing changes are not age dependent	p. 34
Supplementary Figure 11.	Graphical abstract	p. 35
Supplementary References		p. 36

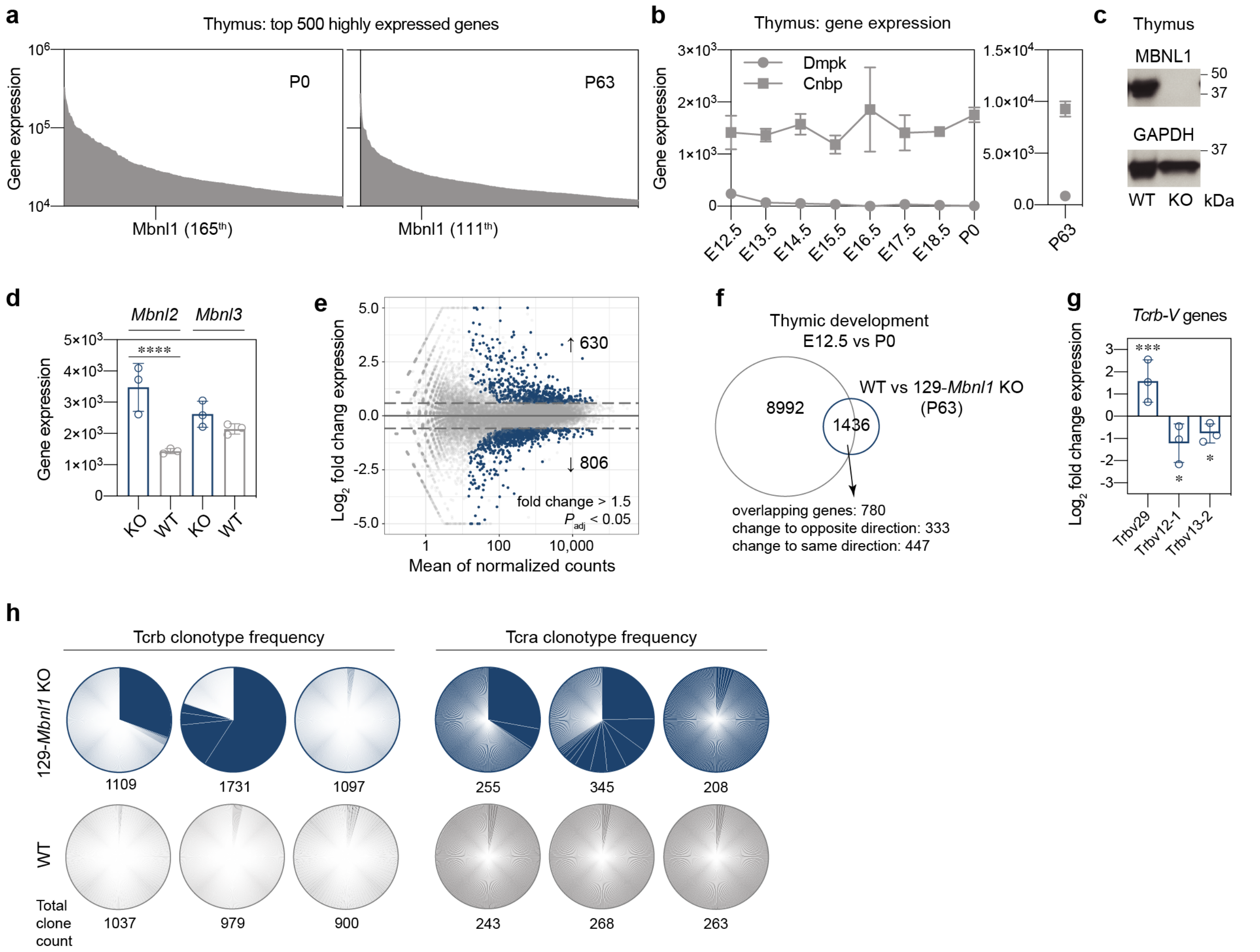

Supplementary Fig. 1. Gene expression changes in 129-*Mbnl1* KO thymus

a Top 500 highly expressed genes in postnatal day 0 (P0; n = 2) and 63 (P63; n = 3) thymus RNA-seq data. *Mbnl1* is the 165th and 111th highest expressed gene at P0 and P63, respectively. P0 RNA-seq data were obtained from GSE107910[1] and P63 represents our RNA-seq.

b *Dmpk* and *Cnbp* gene expression levels during thymus organogenesis and in the postnatal gland. RNA-seq was performed at embryonic (E) days: 12.5 (n = 3), 13.5 (n = 3), 14.5 (n = 2), 15.5 (n = 2), 16.5 (n = 3), 17.5 (n = 2), 18.5 (n = 2) and postnatal day 0 (P0; n = 2) and 63 (P63; n = 3). Data from embryonic and P0 thymi were obtained from GSE107910[1] and P63 represents our RNA-seq. Points connected by lines show mean expression ± standard deviation (SD).

c MBNL1 protein is not detectable in *Mbnl1* knockout (KO) thymus by western blot. WT - wild type. Uncropped and unprocessed scans of the blots containing molecular weight markers are provided in the Source Data file.

d *Mbnl2* gene expression increases in *Mbnl1* KO (n = 3) compared to WT (n = 3) thymus. *Mbnl3* expression level is unchanged. Bar graph shows mean expression ± SD. DESeq2; **** adjusted P value (P_{adj}) < 0.0001.

e Differential gene expression analysis for 129-*Mbnl1* KO. Blue dots on the volcano plot represent differentially expressed genes in *Mbnl1* KO thymus RNA-seq.

f Venn diagram shows differentially expressed genes during embryonic thymus organogenesis (embryonic day (E)12.5 vs P0) and at postnatal day 63 thymus with depleted Mbnl1 protein (WT vs 129-*Mbnl1* KO). Data from embryonic and P0 thymi were obtained from GSE107910[1] and P63 represents our RNA-seq.

g TCR beta variable genes mis-expression in 129-*Mbnl1* KO (n = 3) compared to WT (n = 3) thymus RNA-seq. Bar graph shows mean ± SD. DESeq2; * $P_{adj} < 0.05$, *** $P_{adj} = 0.0005$.

h Pie charts represent Tcrb and Tcra clonotype frequencies in 129-*Mbnl1* KO (n =3) and WT (n = 3) thymic RNA-seq.

Source data are provided as the Source Data and Supplementary Data 1 files.

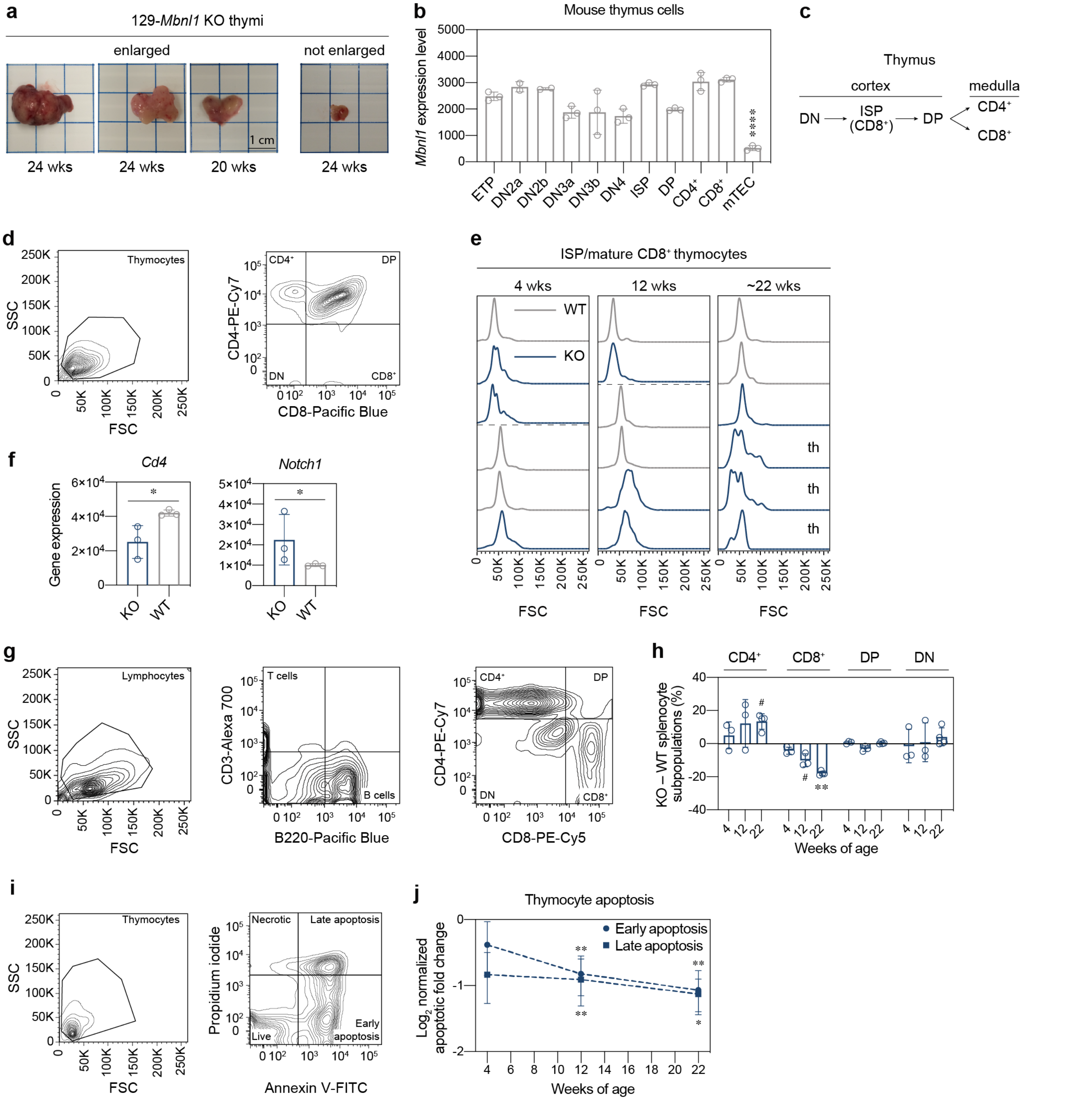

Supplementary Fig. 2. Thymic pathology in 129-*Mbnl1* KO

a Photos of enlarged/not enlarged thymi in 129-*Mbnl1* KO at 20-24 weeks of age (wks).

b *Mbnl1* expression in mouse Early T lineage Precursor (ETP; CD4$^-$,CD8$^-$, CD11b$^-$, CD11c$^-$, CD19$^-$, NK1.1$^-$, TCR$^-$, CD44^{hi}, CD117^{hi}, CD25$^-$), Double Negative 2a (DN2a; CD4$^-$, CD8$^-$, CD11b$^-$, CD11c$^-$, CD19$^-$, NK1.1$^-$, TCR$^-$, CD44^{hi}, CD117^{hi}, CD25^{hi}), Double Negative 2b (DN2b; CD4$^-$, CD8$^-$, CD11b$^-$, CD11c$^-$, CD19$^-$, NK1.1$^-$, TCR$^-$, CD44^{hi}, CD117^{int}, CD25^{hi}), Double Negative 3a (DN3a; CD4$^-$, CD8$^-$, CD11b$^-$, CD11c$^-$, CD19$^-$, NK1.1$^-$, TCR$^-$, CD44$^-$, CD25^{hi}, CD28$^-$), Double-Negative 3b (DN3b; CD4$^-$, CD8$^-$, CD11b$^-$, CD11c$^-$, CD19$^-$, NK1.1$^-$, TCR$^-$, CD44$^-$, CD25^{hi}, CD28$^+$), Double Negative 4 (DN4; CD4$^-$, CD8$^-$, CD11b$^-$, CD11c$^-$, CD19$^-$, NK1.1$^-$, TCR$^-$, CD44$^-$, CD25$^-$, CD28$^+$), Immature Single Positive (ISP; CD4$^-$, CD8$^+$, TCR$^{-/lo}$, CD24^{hi}), Double Positive (DP; CD4$^+$, CD8$^+$, TCR$^{-/lo}$, CD69$^-$), Mature CD4 Single

Positive ($CD4^+$; $CD4^+$, $CD8^-$, TCR^{hi}, $CD24^{-/lo}$), Mature CD8 Single Positive ($CD8^+$; $CD4^-$, $CD8^+$, TCR^{hi}, $CD24^{-/lo}$), and Medullary Thymic Epithelial Cells (mTEC; $CD45^-$, $EpCAM^+$, $Ly51^-$, $MHCII^{hi}$). Gene expression obtained from the BloodSpot database (servers.binf.ku.dk/bloodspot)[2]; Dataset: Mouse ImmGen Microarray - GSE15907 (Immunological Genome Project; www.immgen.org). Bar graph shows mean expression ± SD. Significant difference was determined by Dunnett's multiple comparisons test. **** $P_{adj} < 0.0001$.

c Mouse thymocyte development scheme.

d Schematic of gating strategy for the flow cytometry analysis of thymocyte subpopulations.

e Flow cytometry Forward Scatter (FSC) parameter plots showing different relative thymocyte $CD8^+$ cell sizes in 129-*Mbnl1* KO (th - thymus hyperplasia).

f *Cd4* and *Notch1* gene expression decrease and increases in *Mbnl1* KO (n = 3) compared to WT (n = 3) thymus, respectively. Bar graphs show mean expression ± SD. DESeq2; * $P_{adj} < 0.05$.

g Schematic of gating strategy for the flow cytometry analysis of splenocytes.

h Splenocyte composition at 4 (WT = 3; KO = 3), 8 (WT = 3; KO = 3) and ~22 (WT = 2; KO = 4) wks. $CD4^+$, $CD8^+$, $CD4^+CD8^+$ double positive (DP) and $CD4^-CD8^-$ double negative (DN) thymocytes were analyzed by flow cytometry. Graph represents mean KO - WT subpopulations ± SD. Significant difference was determined by the two-tailed t-test: # $P = 0.065$ ($CD4^+$) and 0.079 ($CD8^+$), ** $P = 0.005$.

i Schematic of gating strategy for the flow cytometry analysis of dexamethasone-induced apoptosis.

j Thymocytes derived from 4, 12 and ~22 wks 129-*Mbnl* KO mice (n = 3 for KO and WT at each time point) are resistant to 10^{-7} M dexamethasone-induced apoptosis. Propidium iodide (PI) and Annexin V markers were used for early and late apoptosis, respectively. Results were normalized to untreated thymocytes derived from the same animal. Graph shows mean KO/WT fold change ± SD. Significant difference was determined by two-tailed t-test test: * $P < 0.05$, ** $P < 0.01$.

Source data are provided as a Supplementary Data 1 file.

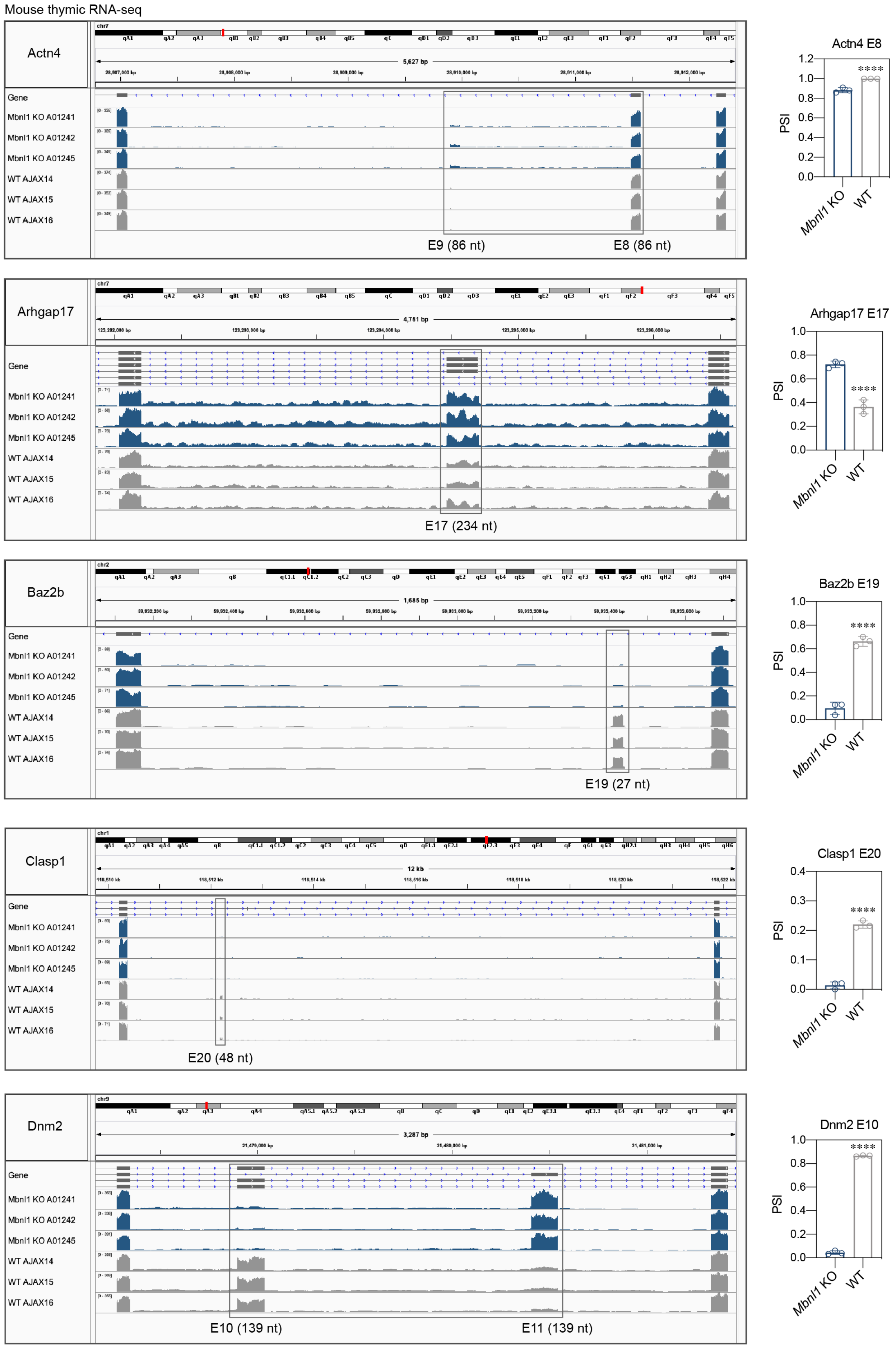

Mouse thymic RNA-seq
Actn4
Gene
Mbnl1 KO A01241
Mbnl1 KO A01242
Mbnl1 KO A01245
WT AJAX14
WT AJAX15
WT AJAX16
E9 (86 nt)
E8 (86 nt)
Actn4 E8
PSI
****
Mbnl1 KO
WT
Arhgap17
Gene
Mbnl1 KO A01241
Mbnl1 KO A01242
Mbnl1 KO A01245
WT AJAX14
WT AJAX15
WT AJAX16
E17 (234 nt)
Arhgap17 E17
PSI
****
Mbnl1 KO
WT
Baz2b
Gene
Mbnl1 KO A01241
Mbnl1 KO A01242
Mbnl1 KO A01245
WT AJAX14
WT AJAX15
WT AJAX16
E19 (27 nt)
Baz2b E19
PSI
****
Mbnl1 KO
WT
Clasp1
Gene
Mbnl1 KO A01241
Mbnl1 KO A01242
Mbnl1 KO A01245
WT AJAX14
WT AJAX15
WT AJAX16
E20 (48 nt)
Clasp1 E20
PSI
****
Mbnl1 KO
WT
Dnm2
Gene
Mbnl1 KO A01241
Mbnl1 KO A01242
Mbnl1 KO A01245
WT AJAX14
WT AJAX15
WT AJAX16
E10 (139 nt)
E11 (139 nt)
Dnm2 E10
PSI
****
Mbnl1 KO
WT

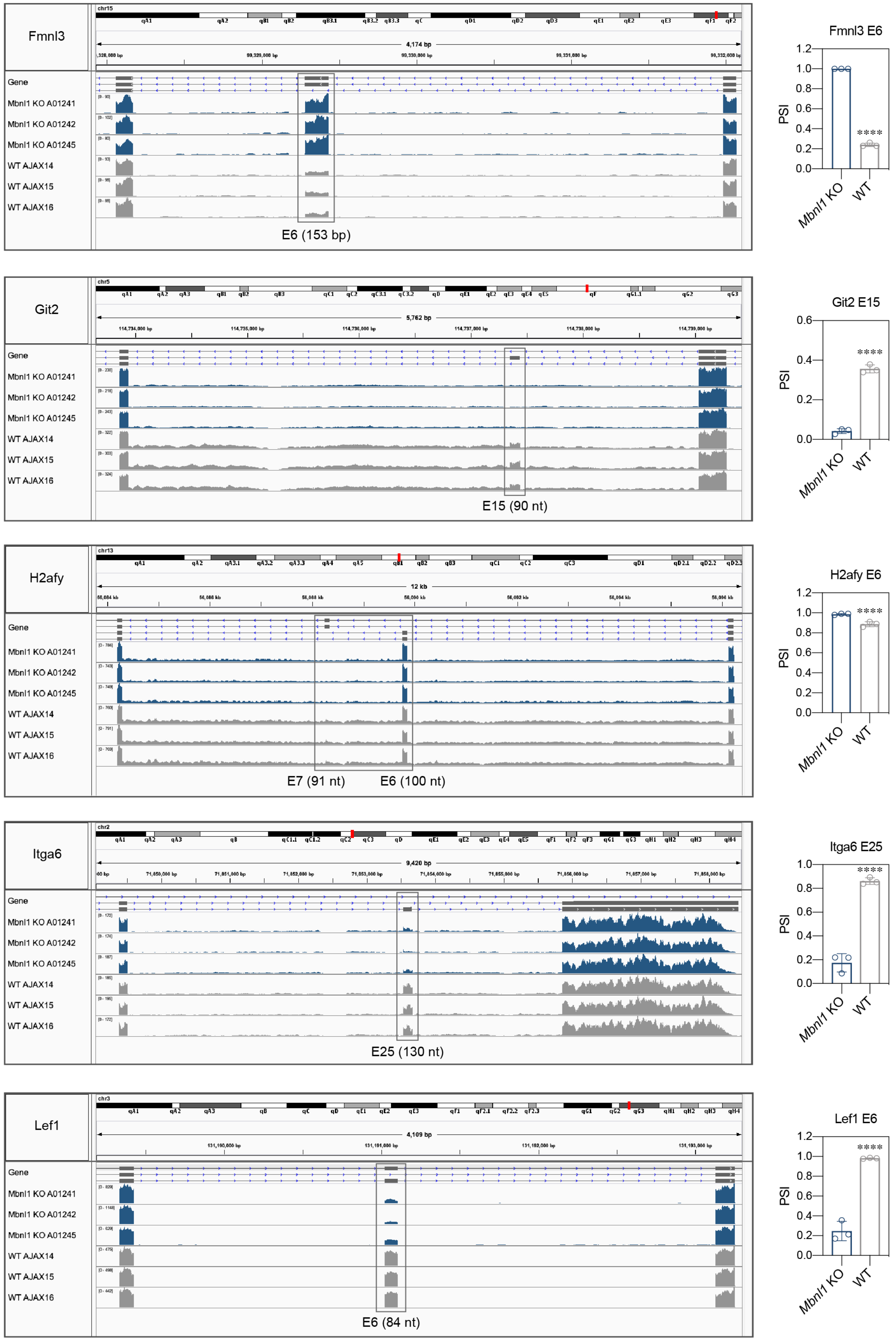
Fmnl3
Gene
Mbnl1 KO A01241
Mbnl1 KO A01242
Mbnl1 KO A01245
WT AJAX14
WT AJAX15
WT AJAX16
E6 (153 bp)
Fmnl3 E6
PSI
Mbnl1 KO
WT
****
Git2
Gene
Mbnl1 KO A01241
Mbnl1 KO A01242
Mbnl1 KO A01245
WT AJAX14
WT AJAX15
WT AJAX16
E15 (90 nt)
Git2 E15
PSI
Mbnl1 KO
WT
****
H2afy
Gene
Mbnl1 KO A01241
Mbnl1 KO A01242
Mbnl1 KO A01245
WT AJAX14
WT AJAX15
WT AJAX16
E7 (91 nt)
E6 (100 nt)
H2afy E6
PSI
Mbnl1 KO
WT
****
Itga6
Gene
Mbnl1 KO A01241
Mbnl1 KO A01242
Mbnl1 KO A01245
WT AJAX14
WT AJAX15
WT AJAX16
E25 (130 nt)
Itga6 E25
PSI
Mbnl1 KO
WT
****
Lef1
Gene
Mbnl1 KO A01241
Mbnl1 KO A01242
Mbnl1 KO A01245
WT AJAX14
WT AJAX15
WT AJAX16
E6 (84 nt)
Lef1 E6
PSI
Mbnl1 KO
WT
****

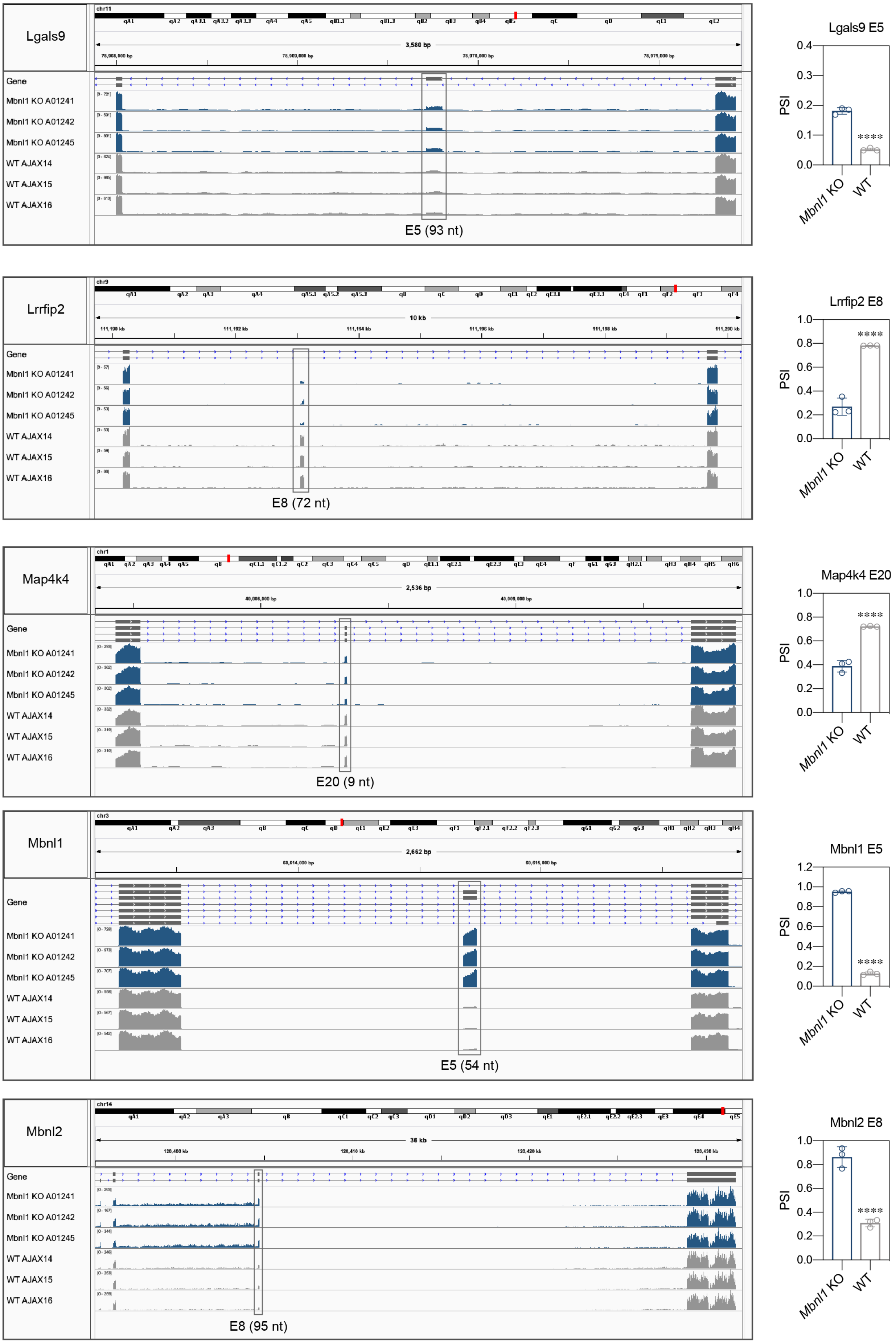
Lgals9
Gene
Mbnl1 KO A01241
Mbnl1 KO A01242
Mbnl1 KO A01245
WT AJAX14
WT AJAX15
WT AJAX16
E5 (93 nt)
Lgals9 E5
PSI
0.4
0.3
0.2
0.1
0.0
Mbnl1 KO
WT
****
Lrrfip2
Gene
Mbnl1 KO A01241
Mbnl1 KO A01242
Mbnl1 KO A01245
WT AJAX14
WT AJAX15
WT AJAX16
E8 (72 nt)
Lrrfip2 E8
PSI
1.0
0.8
0.6
0.4
0.2
0.0
Mbnl1 KO
WT
****
Map4k4
Gene
Mbnl1 KO A01241
Mbnl1 KO A01242
Mbnl1 KO A01245
WT AJAX14
WT AJAX15
WT AJAX16
E20 (9 nt)
Map4k4 E20
PSI
1.0
0.8
0.6
0.4
0.2
0.0
Mbnl1 KO
WT
****
Mbnl1
Gene
Mbnl1 KO A01241
Mbnl1 KO A01242
Mbnl1 KO A01245
WT AJAX14
WT AJAX15
WT AJAX16
E5 (54 nt)
Mbnl1 E5
PSI
1.2
1.0
0.8
0.6
0.4
0.2
0.0
Mbnl1 KO
WT
****
Mbnl2
Gene
Mbnl1 KO A01241
Mbnl1 KO A01242
Mbnl1 KO A01245
WT AJAX14
WT AJAX15
WT AJAX16
E8 (95 nt)
Mbnl2 E8
PSI
1.0
0.8
0.6
0.4
0.2
0.0
Mbnl1 KO
WT
****

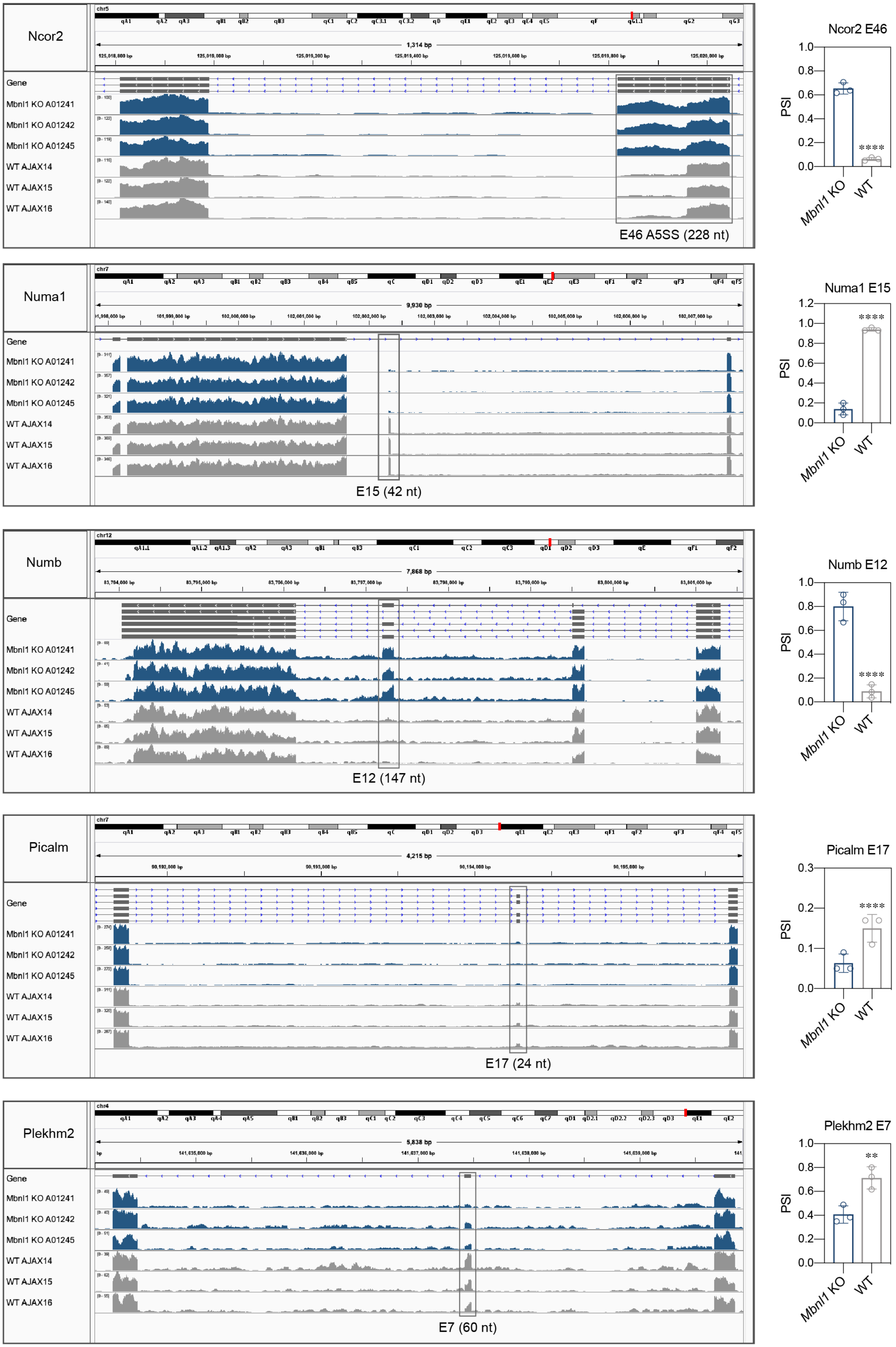
Ncor2
Gene
Mbnl1 KO A01241
Mbnl1 KO A01242
Mbnl1 KO A01245
WT AJAX14
WT AJAX15
WT AJAX16
E46 A5SS (228 nt)
Ncor2 E46
PSI
****
Mbnl1 KO
WT
Numa1
E15 (42 nt)
Numa1 E15
Numb
E12 (147 nt)
Numb E12
Picalm
E17 (24 nt)
Picalm E17
Plekhm2
E7 (60 nt)
Plekhm2 E7
**

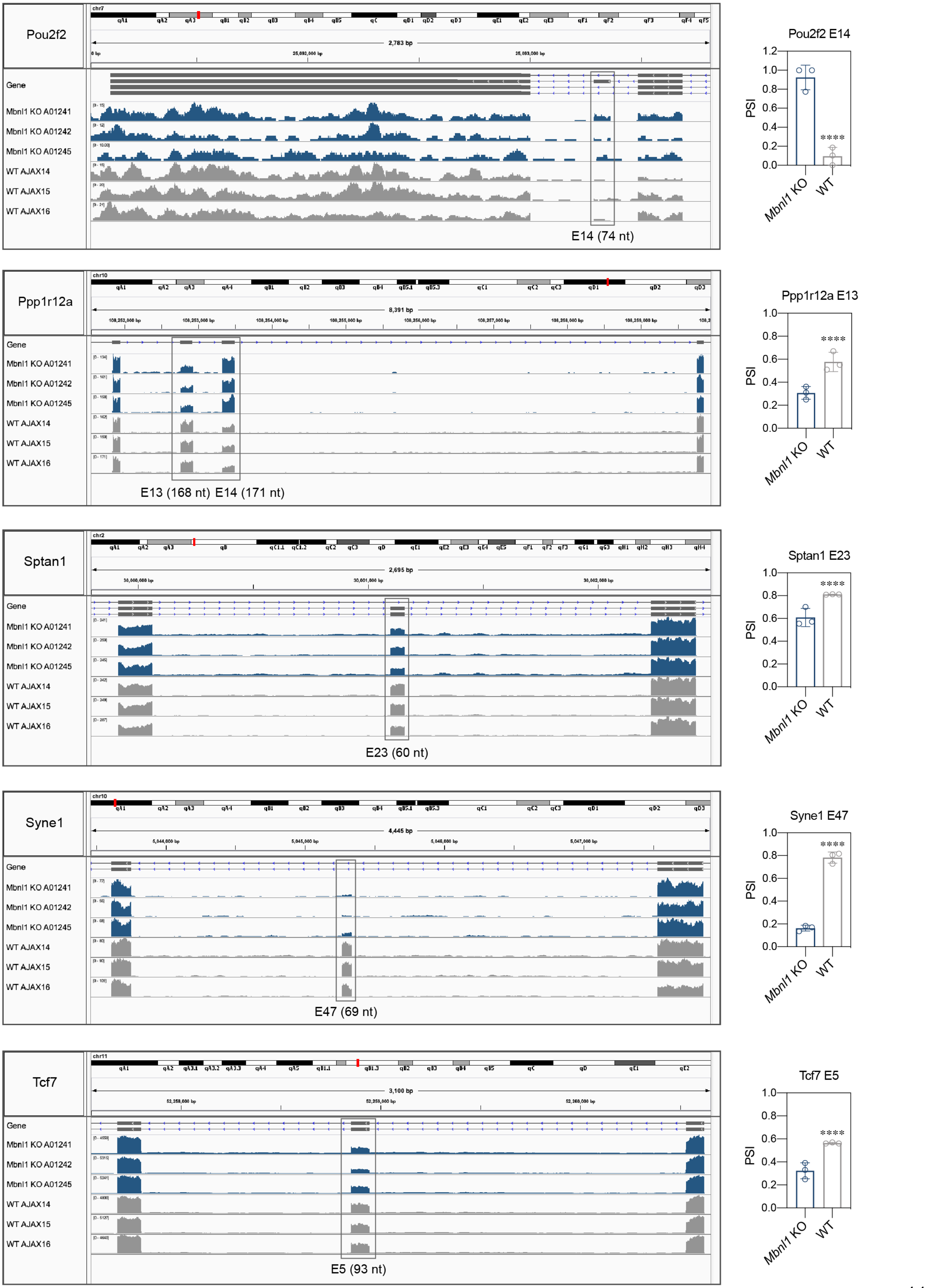
Pou2f2
chr7
2,783 bp
Gene
Mbnl1 KO A01241
Mbnl1 KO A01242
Mbnl1 KO A01245
WT AJAX14
WT AJAX15
WT AJAX16
E14 (74 nt)
Pou2f2 E14
PSI
1.2
1.0
0.8
0.6
0.4
0.2
0.0
****
Mbnl1 KO
WT
Ppp1r12a
chr10
8,391 bp
Gene
Mbnl1 KO A01241
Mbnl1 KO A01242
Mbnl1 KO A01245
WT AJAX14
WT AJAX15
WT AJAX16
E13 (168 nt) E14 (171 nt)
Ppp1r12a E13
PSI
1.0
0.8
0.6
0.4
0.2
0.0
****
Mbnl1 KO
WT
Sptan1
chr2
2,695 bp
Gene
Mbnl1 KO A01241
Mbnl1 KO A01242
Mbnl1 KO A01245
WT AJAX14
WT AJAX15
WT AJAX16
E23 (60 nt)
Sptan1 E23
PSI
1.0
0.8
0.6
0.4
0.2
0.0
****
Mbnl1 KO
WT
Syne1
chr10
4,445 bp
Gene
Mbnl1 KO A01241
Mbnl1 KO A01242
Mbnl1 KO A01245
WT AJAX14
WT AJAX15
WT AJAX16
E47 (69 nt)
Syne1 E47
PSI
1.0
0.8
0.6
0.4
0.2
0.0
****
Mbnl1 KO
WT
Tcf7
chr11
3,100 bp
Gene
Mbnl1 KO A01241
Mbnl1 KO A01242
Mbnl1 KO A01245
WT AJAX14
WT AJAX15
WT AJAX16
E5 (93 nt)
Tcf7 E5
PSI
1.0
0.8
0.6
0.4
0.2
0.0
****
Mbnl1 KO
WT

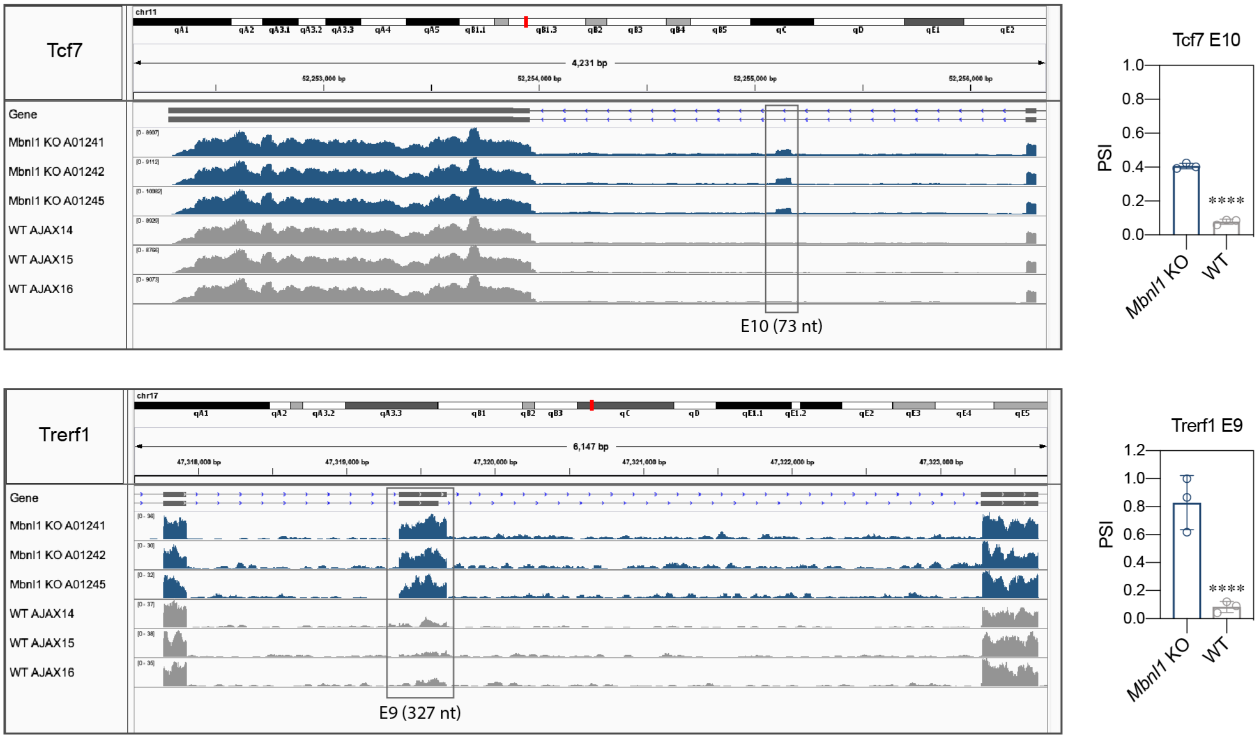

Supplementary Fig. 3. Genome browser views of selected mouse alternative splicing events

Genome browser view of selected alternative splicing (AS) events from thymus *Mbnl1* KO (n = 3) and WT (n = 3) RNA-seq data. *Mbnl1* KO and WT are represented by blue and grey wiggle plots, respectively. Bar graphs show mean percent spliced in (PSI) ± SD. rMATS; ** FDR < 0.01, **** FDR < 0.0001.

Source data are provided as a Supplementary Data 1 file.

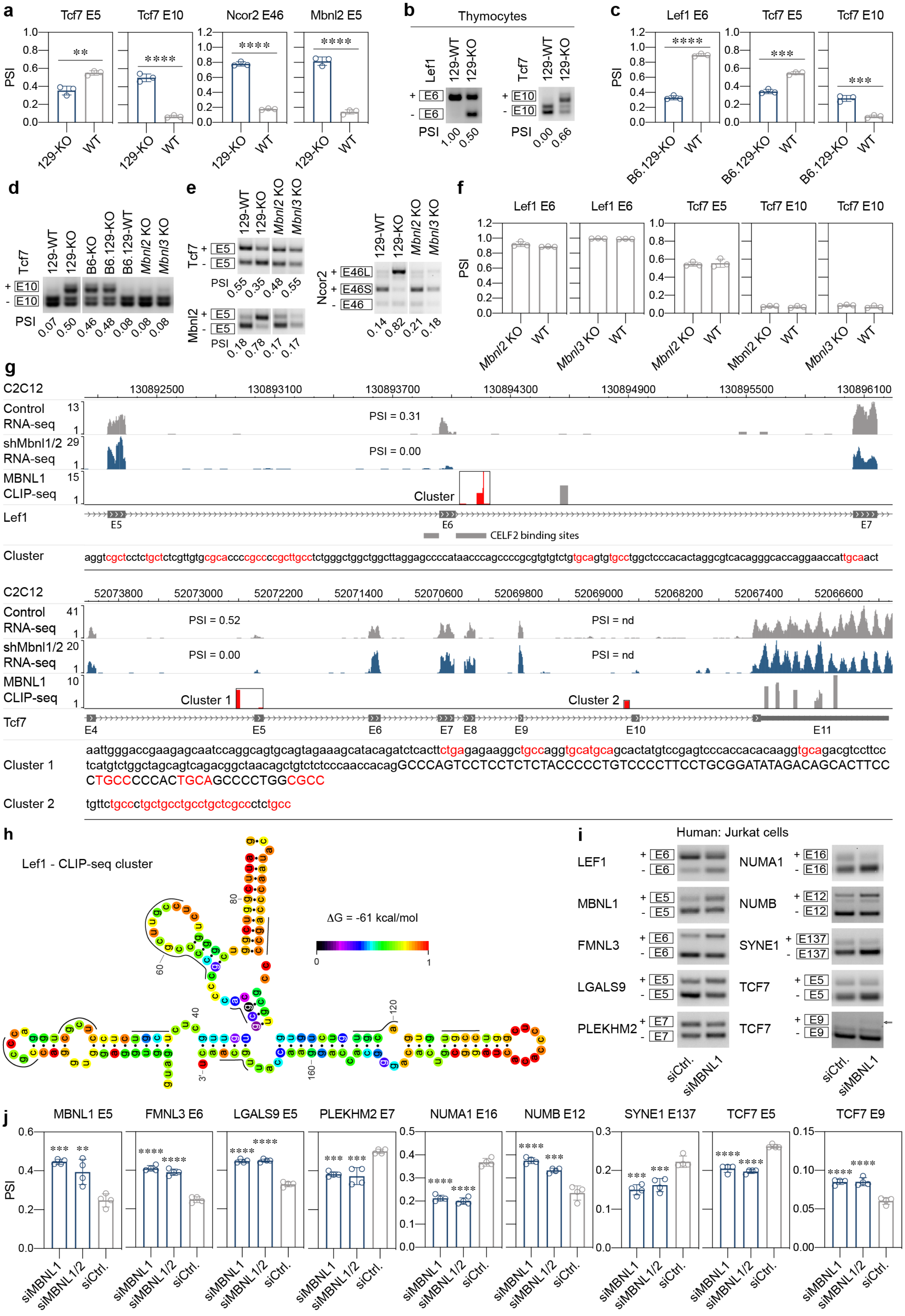

a
Tcf7 E5
Tcf7 E10
Ncor2 E46
Mbnl2 E5
PSI
129-KO
WT
b
Thymocytes
Lef1
Tcf7
129-WT
129-KO
PSI 1.00 0.50
PSI 0.00 0.66
c
Lef1 E6
Tcf7 E5
Tcf7 E10
B6.129-KO
WT
d
Tcf7
129-WT 129-KO B6-KO B6.129-KO B6.129-WT Mbnl2 KO Mbnl3 KO
PSI 0.07 0.50 0.46 0.48 0.08 0.08 0.08
e
Tcf7
PSI 0.55 0.35 0.48 0.55
Mbnl2
PSI 0.18 0.78 0.17 0.17
Ncor2
E46L
E46S
E46
0.14 0.82 0.21 0.18
f
Lef1 E6
Tcf7 E5
Tcf7 E10
Mbnl2 KO
Mbnl3 KO
g
C2C12
Control RNA-seq
shMbnl1/2 RNA-seq
MBNL1 CLIP-seq
PSI = 0.31
PSI = 0.00
Cluster
Lef1
CELF2 binding sites
Cluster
aggtcgctcctctgctctcgttgtgcgcacccgcccgcttgcctctgggctggctggcttaggagccccataacccagccccgcgtgtgtctgtgcagtgtgcctggctcccacactaggcgtcacagggcaccaggaaccattgcaact
PSI = 0.52
PSI = 0.00
PSI = nd
Cluster 1
Cluster 2
Tcf7
Cluster 1
aattgggaccgaagagcaatccaggcagtgcagtagaaagcatacagatctcacttctgagagaaggctgccaggtgcatgcagcactatgtccgagtcccaccacacaaggtgcagacgtccttcc
tcatgtctggctagcagtcagacggctaacagctgtctctcccaaccacagGCCCAGTCCTCCTCTCTACCCCCTGTCCCCTTCCTGCGGATATAGACAGCACTTCC
CTGCCCCCACTGCAGCCCCTGGCGCC
Cluster 2
tgttctgccctgctgcctgcctgctcgccctctgcc
h
Lef1 - CLIP-seq cluster
ΔG = -61 kcal/mol
i
Human: Jurkat cells
LEF1
MBNL1
FMNL3
LGALS9
PLEKHM2
NUMA1
NUMB
SYNE1
TCF7
siCtrl.
siMBNL1
j
MBNL1 E5
FMNL3 E6
LGALS9 E5
PLEKHM2 E7
NUMA1 E16
NUMB E12
SYNE1 E137
TCF7 E5
TCF7 E9
PSI
siMBNL1
siMBNL1/2
siCtrl.

Supplementary Fig. 4. MBNL1 directly regulates Lef1 E6

a Tcf7 E5, Tcf7 E10, Ncor2 E46 and Mbnl2 E5 splicing changes in 129-*Mbnl1* KO (n = 3) compared to WT (n = 3) thymi detected by RT-PCR. Bar graphs show mean PSI ± SD. Significant differences were determined by the two-tailed t-test: ** P = 0.006, **** P < 0.0001.

b Representative gel of Lef1 E6 and Tcf7 E10 RT-PCR assays of thymocytes from 129-*Mbnl1* KO. Mean PSI values are indicated below the photo.

c Lef1 E6, Tcf7 E5 and Tcf7 E10 splicing changes in B6.129-*Mbnl1* KO (n = 3) compared to WT (n = 3) thymi detected by RT-PCR. Bar graphs show mean PSI ± SD. Significant differences were determined by the two-tailed t-test: *** P = 0.0003, **** P < 0.0001.

d Representative gel of Tcf7 E10 RT-PCR assays of thymi from the different *Mbnl1* KO strains as well as *Mbnl2* and *Mbnl3* KOs. Mean PSI values are indicated below the photo.

e Representative gel of Tcf7 E5, Ncor2 E46 and Mbnl2 E5 RT-PCR assays of thymi from the *Mbnl1*, *Mbnl2* and *Mbnl3* KOs. Mean PSI values are indicated below the photo.

f Lef1 E6, Tcf7 E5 and Tcf7 E10 splicing changes in *Mbnl2* KO (n = 3), *Mbnl3* KO (n = 3) compared to WT (n = 3) thymi detected by RT-PCR. Bar graphs show mean PSI ± SD. Significant difference were determined by the two-tailed t-test. There are no statistically significant differences.

g Genome browser view of RNA-seq generated from C2C12 cells with compound Mbnl1 and Mbnl2 knock-down by shRNA. Cluster of MBNL1 CLIP-seq reads indicates MBNL1 binding sites in C2C12. YGCY/A motifs are marked (red). Predicted CELF2 binding sites are shown.

h Proposed secondary structure of the 5'-end of Lef1 intron 6 with potential MBNL1-binding motifs indicated (YGCY/A) (black line). The optimal thermodynamic stability of the structure is expressed in Gibbs free energy (ΔG) in kcal/mol for the reaction at 37°C using RNAfold software (http://rna.tbi.univie.ac.at/). The color annotation represents the pair probabilities of the nucleotides.

i Representative gel photos of AS events assessed by RT-PCR assays in human model of T cells (Jurkat) with MBNL1 knock-down by siRNA.

j MBNL1 (n = 4) and compound MBNL1 and MBNL2 (n = 4) knock-down causes AS changes in Jurkat cells. Bar graphs show mean PSI ± SD. The significant differences were determined by Dunnett's multiple comparison test: ** P_{adj} < 0.01, *** P_{adj} < 0.001, **** P_{adj} < 0.0001. Note that LEF1 E6 graph is Fig. 3i.

Source data are provided as the Source Data and Supplementary Data 1 files.

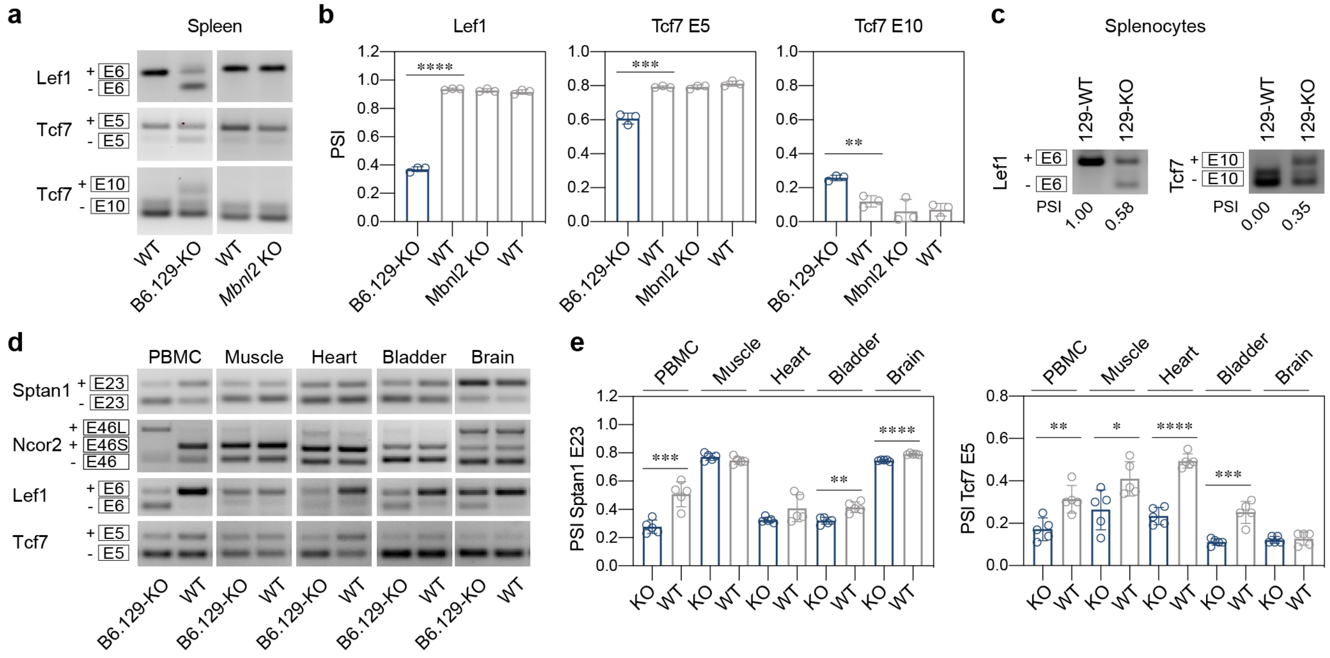

Supplementary Fig. 5. Alternative splicing changes in *Mbnl1* KO splenocytes and PBMCs

a Representative gel of Lef1 E6 and Tcf7 E5 RT-PCR assays on spleen isolated from the 129-*Mbnl1* KO and WT.

b Lef1 E6, Tcf7 E5 and Tcf7 E10 splicing changes in B6.129-*Mbnl1* KO (n = 3) compared to WT (n = 3) spleens detected by RT-PCR. Bar graphs show mean PSI ± SD. Significant differences were determined by the two-tailed t-test: *** $P = 0.0006$, **** $P < 0.0001$.

c Representative gels of Lef1 E6 and Tcf7 E10 RT-PCR assays on splenocytes isolated from the 129-*Mbnl1* KO (n = 2) and WT. Mean PSI values are provided below the photo.

d Representative gels of AS events assessed by RT-PCR assays on B6.129-*Mbnl1* KO and WT PBMCs, muscle (tibialis anterior), heart, bladder and brain.

e B6.129-*Mbnl1* KO (n = 5) causes the same AS changes in PBMCs, muscle, heart, bladder and brain. Bar graphs show mean PSI ± SD. Significant differences were determined by the two-tailed t-test test: * $P < 0.05$, ** $P < 0.01$, *** $P < 0.001$, **** $P < 0.0001$.

Source data are provided as the Source Data and Supplementary Data 1 files.

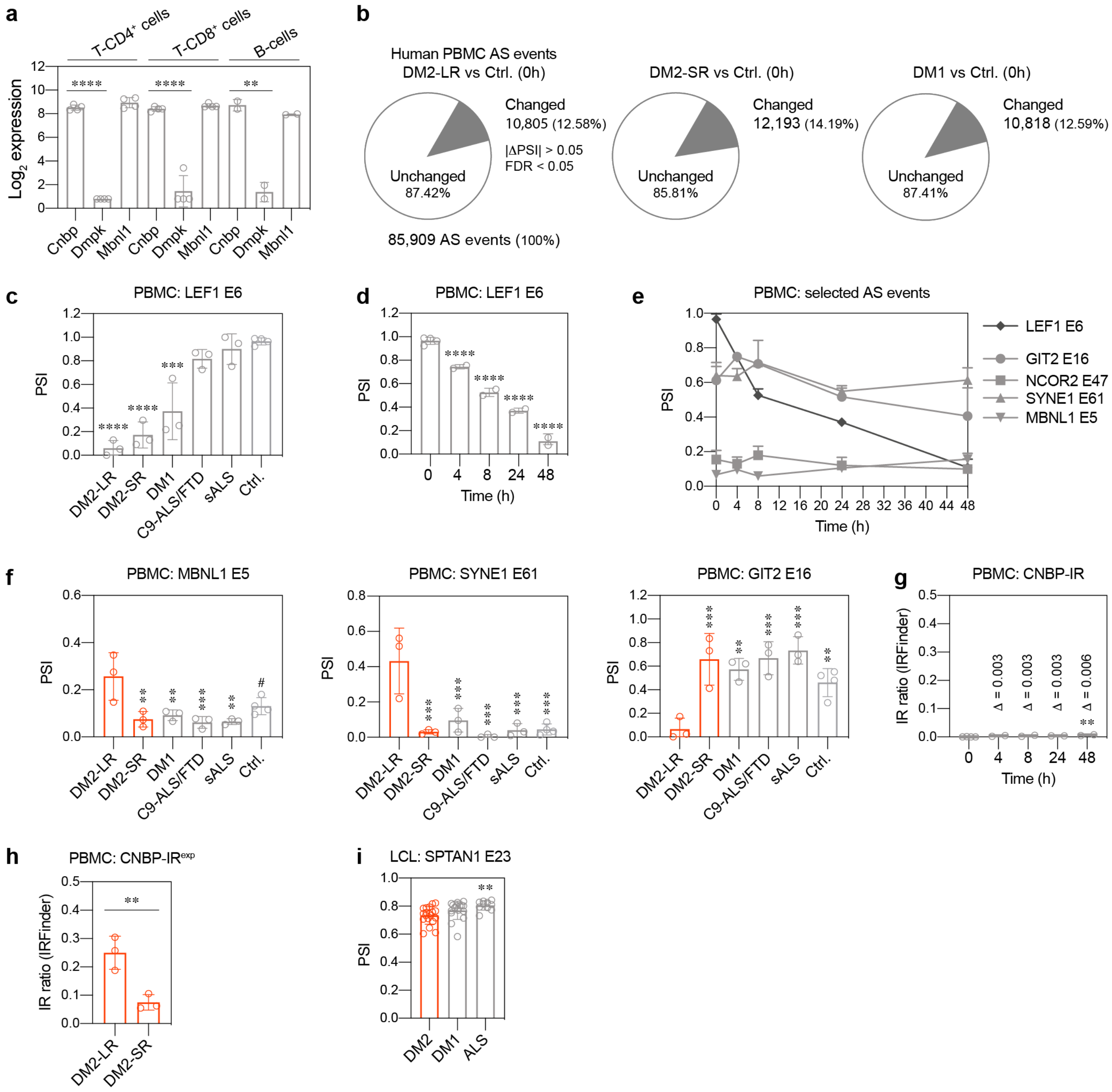

Supplementary Fig. 6. Alternative splicing changes in DM2 PBMCs

a *Cnbp*, *Dmpk* and *Mbnl1* expression in mouse T-CD4$^+$ cells (B220$^-$, CD3$^+$, CD19$^-$, CD4$^+$, CD8$^-$), T-CD8$^+$ cells (B220$^-$, CD3$^+$, CD19$^-$, CD4$^-$, CD8$^+$) and B-cells (B220$^+$, CD3$^-$, CD19$^+$). Gene expression obtained from the BloodSpot database (servers.binf.ku.dk/bloodspot)[2]; Dataset: Mouse Normal (RNA-seq) - GSE60101. Bar graph shows mean expression ± SD. Significant differences were determined by the two-tailed t-test test: ** $P < 0.008$, **** $P < 0.0001$.

b Delayed PBMC isolation results in transcriptomic changes. Pie charts represent the proportion of significantly changed AS events (gray triangle) to all detected AS events in DM2-LR (n = 3), DM2-SR (n = 3) and DM1 (n = 3) PBMC RNA-seq samples. RNA-seq from freshly isolated (0h) PBMCs was used as a control - GSE58335[3].

c LEF1 E6 is significantly excluded in DM2-LR, DM2-SR and DM1 RNA-seq data. Bar graph shows mean PSI ± SD. Significant difference was determined by Dunnett's multiple comparison test: *** $P_{adj} = 0.0001$, **** $P_{adj} < 0.0001$.

d Delayed PBMC isolation leads to significant exclusion of LEF1 E6 in unaffected PBMCs. Bar graph shows mean PSI ± SD. rMATS; **** FDR < 0.0001.

e Four AS events resistant to change caused by delayed PBMC isolation. Selected AS events were analyzed in this study and there were no significant changes between 0h and 4/8/24/48 hrs. Points connected by lines show mean PSI ± SD.

f Aberrant MBNL1 E5, SYNE1 E61 and GIT2 E16 splicing in DM2-LR but not in DM2-SR, DM1, C9-ALS/FTD nor sALS RNA-seq PBMC samples. Bar graphs show mean PSI ± SD. Significant difference were determined by Dunnett's multiple comparisons test: # P_{adj} = 0.157, ** P_{adj} < 0.01, *** P_{adj} < 0.001.

g Delayed PBMC isolation does not affect CNBP intron 1 retention (CNBP-IR) in unaffected samples. Bar graph shows mean IR ratio ± SD. Significant difference was determined by Dunnett's multiple comparison test: ** P_{adj} = 0.009.

h Significant differences in CNBP-IR^{exp} between DM2-LR and DM2-SR. Bar graph shows mean IR ratio ± SD. Significant difference was determined by the two-tailed t-test test: ** *P* = 0.009.

i Aberrant SPTAN1 E23 exclusion in a large cohort of DM2-derived LCLs (n = 19); DM1 (n = 16); ALS (n = 10). Bar graph shows mean PSI ± SD. Significant difference was determined by Dunn's multiple comparison test: ** P_{adj} = 0.003.

Source data are provided as a Supplementary Data 1 file.

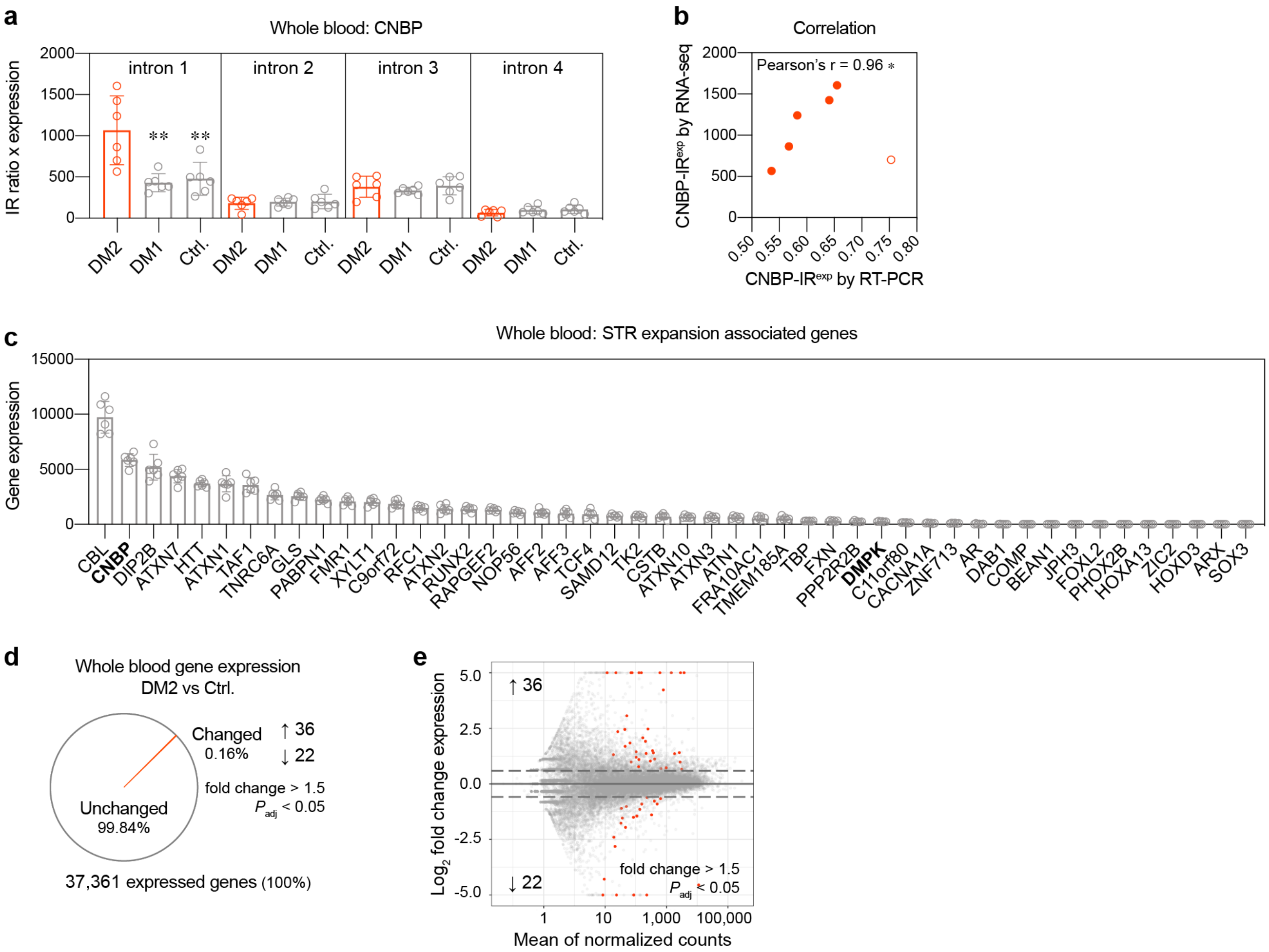

Supplementary Fig. 7. Gene expression changes in DM2 whole blood

a *CNBP* intron 1, 2, 3 and 4 retention analysis in DM2 (n = 6), DM1 (n = 6) and unaffected control (n = 6) samples. Bar graph shows mean IR ratio x CNBP expression ± SD. Significant difference was determined by Dunnett's multiple comparisons test: ** $P_{adj} < 0.004$.

b Correlation between CNBP-IR^{exp} measured by RT-PCR (Fig. 4a) and RNA-seq (Supplementary Fig. 7a). One sample (circle) was excluded from the analysis because 5-times less total RNA (200 ng) was used for library preparation and the sequencing depth was relatively low (29 million uniquely mapped reads). * $P = 0.01$.

c Expression of microsatellite expansion associated genes in unaffected whole blood RNA-seq samples. Bar graph shows mean expression ± SD.

d Gene expression changes in DM2 whole blood. Pie chart represents the proportion of significantly altered genes (orange) to all detected genes (gray) in DM2 (n = 6) compared to unaffected control (n = 6) RNA-seq samples.

e Differential gene expression analysis for DM2 whole blood (n = 6). Orange dots on the volcano plot represent significantly different expressed genes in DM2 compared to unaffected control whole blood samples.

Source data are provided as a Supplementary Data 1 file.

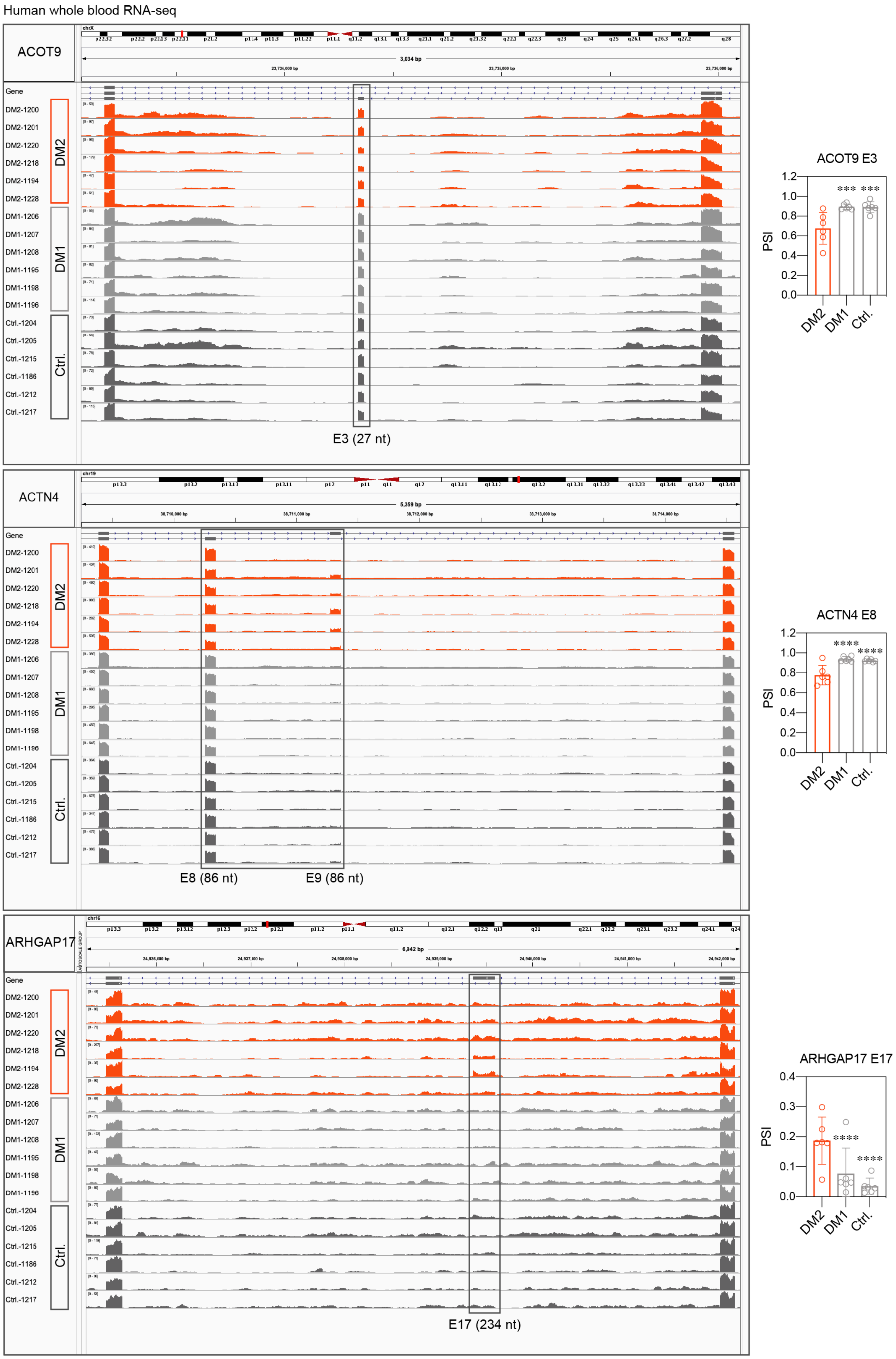
Human whole blood RNA-seq
ACOT9
chrX
3,034 bp
Gene
DM2-1200
DM2-1201
DM2-1220
DM2-1218
DM2-1194
DM2-1228
DM1-1206
DM1-1207
DM1-1208
DM1-1195
DM1-1198
DM1-1196
Ctrl.-1204
Ctrl.-1205
Ctrl.-1215
Ctrl.-1186
Ctrl.-1212
Ctrl.-1217
DM2
DM1
Ctrl.
E3 (27 nt)
ACOT9 E3
PSI
1.2
1.0
0.8
0.6
0.4
0.2
0.0
***
***
ACTN4
chr19
5,359 bp
E8 (86 nt)
E9 (86 nt)
ACTN4 E8
****
****
ARHGAP17
chr16
6,942 bp
E17 (234 nt)
ARHGAP17 E17
0.4
0.3
0.2
0.1
0.0
****
****

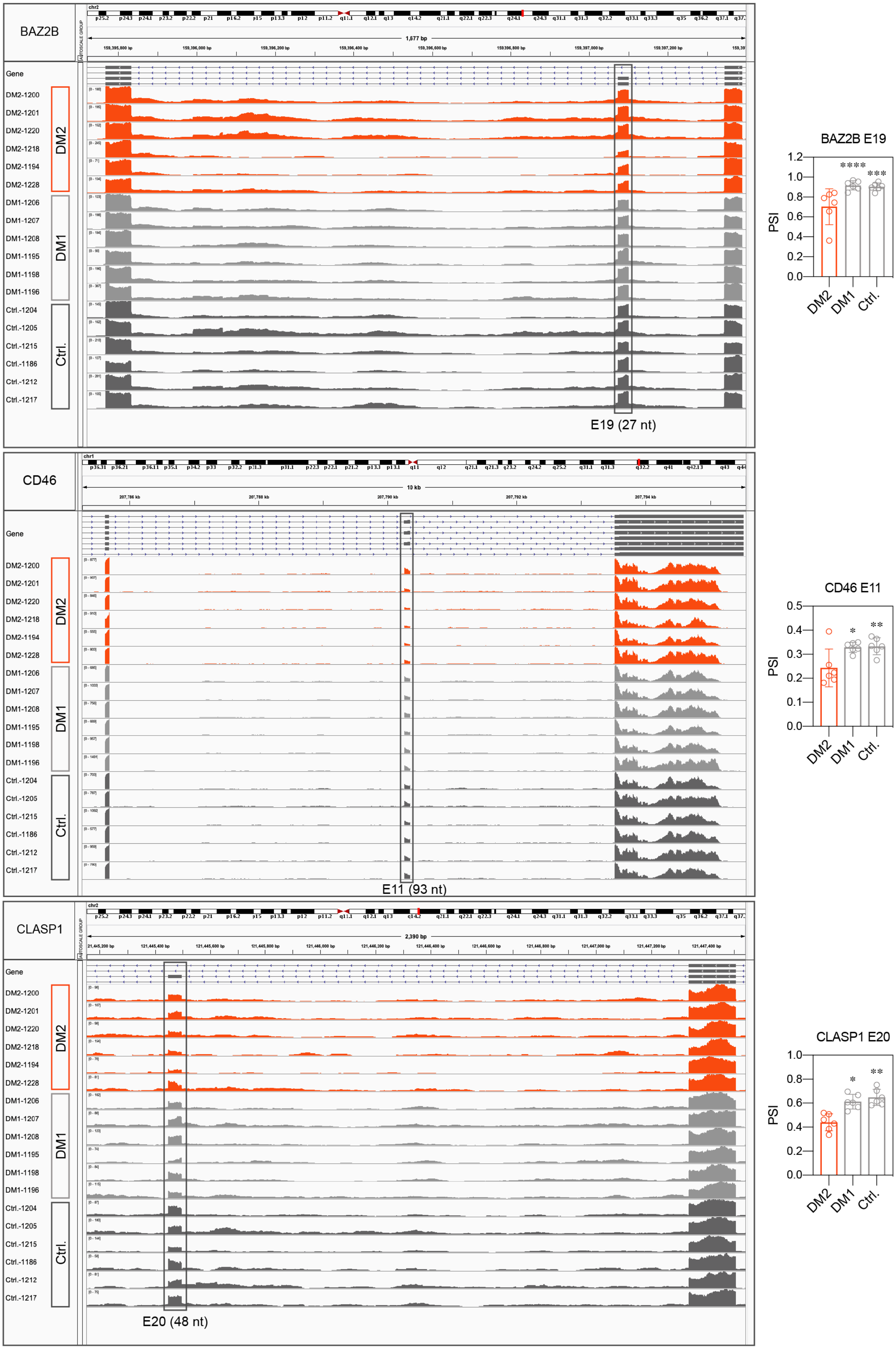
BAZ2B
Gene
DM2-1200
DM2-1201
DM2-1220
DM2-1218
DM2-1194
DM2-1228
DM1-1206
DM1-1207
DM1-1208
DM1-1195
DM1-1198
DM1-1196
Ctrl.-1204
Ctrl.-1205
Ctrl.-1215
Ctrl.-1186
Ctrl.-1212
Ctrl.-1217
DM2
DM1
Ctrl.
E19 (27 nt)
BAZ2B E19
PSI
****
***
CD46
E11 (93 nt)
CD46 E11
*
**
CLASP1
E20 (48 nt)
CLASP1 E20
*
**

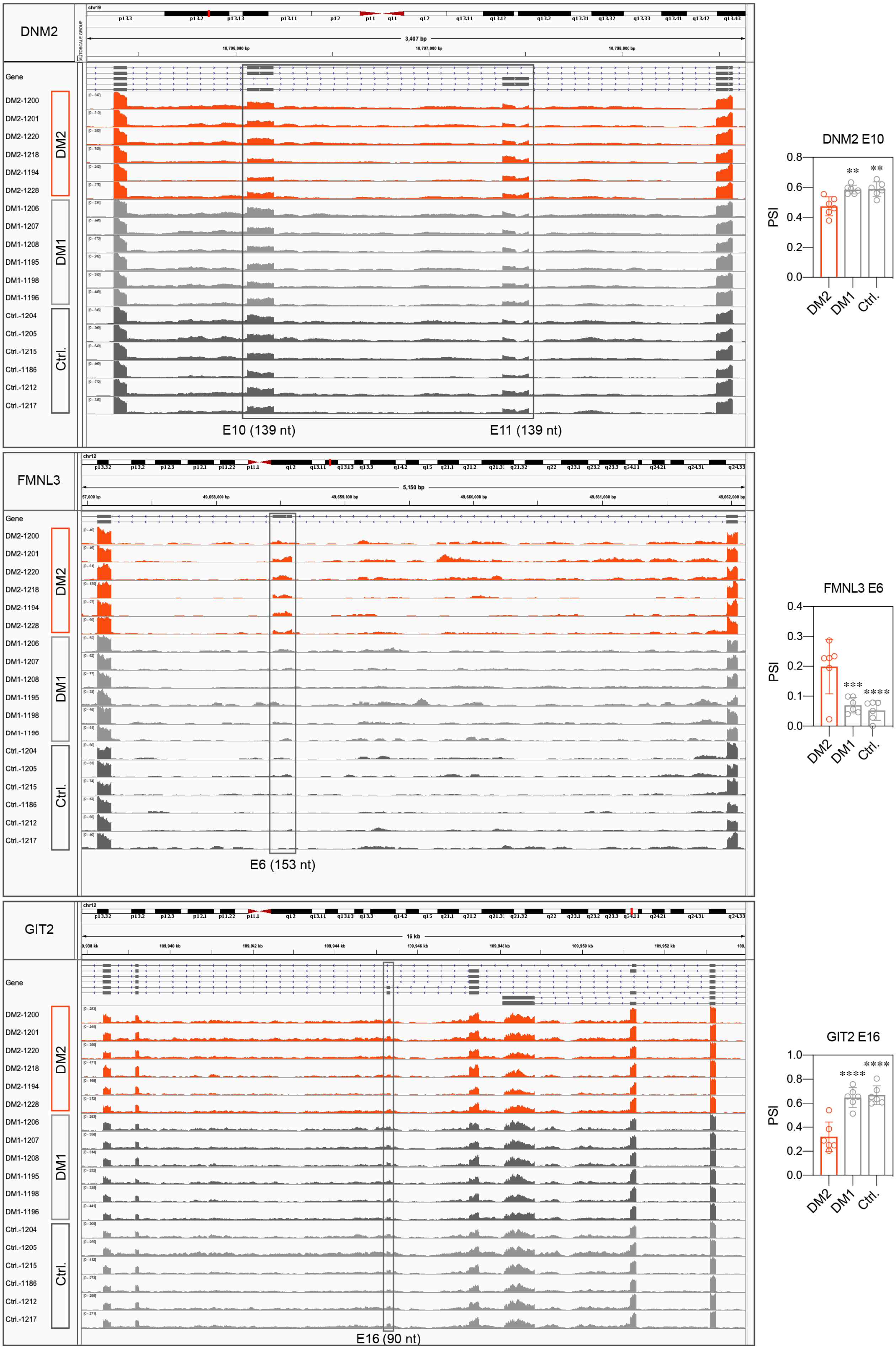
DNM2
Gene
DM2-1200
DM2-1201
DM2-1220
DM2-1218
DM2-1194
DM2-1228
DM1-1206
DM1-1207
DM1-1208
DM1-1195
DM1-1198
DM1-1196
Ctrl.-1204
Ctrl.-1205
Ctrl.-1215
Ctrl.-1186
Ctrl.-1212
Ctrl.-1217
DM2
DM1
Ctrl.
E10 (139 nt)
E11 (139 nt)
DNM2 E10
PSI
0.8
0.6
0.4
0.2
0.0
**
**
FMNL3
E6 (153 nt)
FMNL3 E6
0.4
0.3
0.2
0.1
0.0
***
****
GIT2
E16 (90 nt)
GIT2 E16
1.0
****
****

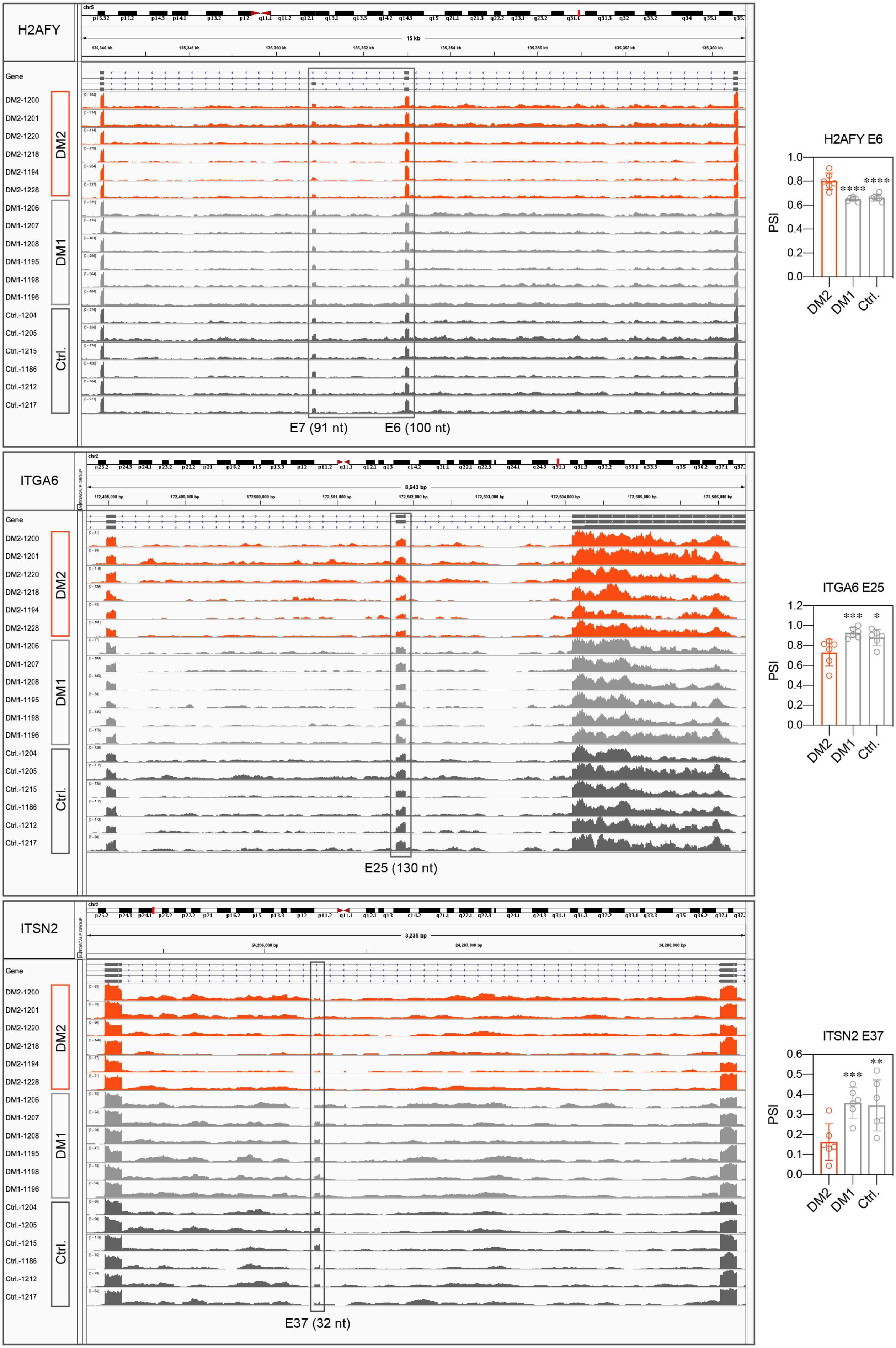
H2AFY
Gene
DM2-1200
DM2-1201
DM2-1220
DM2-1218
DM2-1194
DM2-1228
DM1-1206
DM1-1207
DM1-1208
DM1-1195
DM1-1198
DM1-1196
Ctrl.-1204
Ctrl.-1205
Ctrl.-1215
Ctrl.-1186
Ctrl.-1212
Ctrl.-1217
DM2
DM1
Ctrl.
E7 (91 nt)
E6 (100 nt)
H2AFY E6
PSI
1.0
0.8
0.6
0.4
0.2
0.0
****
****
DM2
DM1
Ctrl.
ITGA6
E25 (130 nt)
ITGA6 E25
1.2
1.0
0.8
0.6
0.4
0.2
0.0
***
*
ITSN2
E37 (32 nt)
ITSN2 E37
0.6
0.5
0.4
0.3
0.2
0.1
0.0
***
**

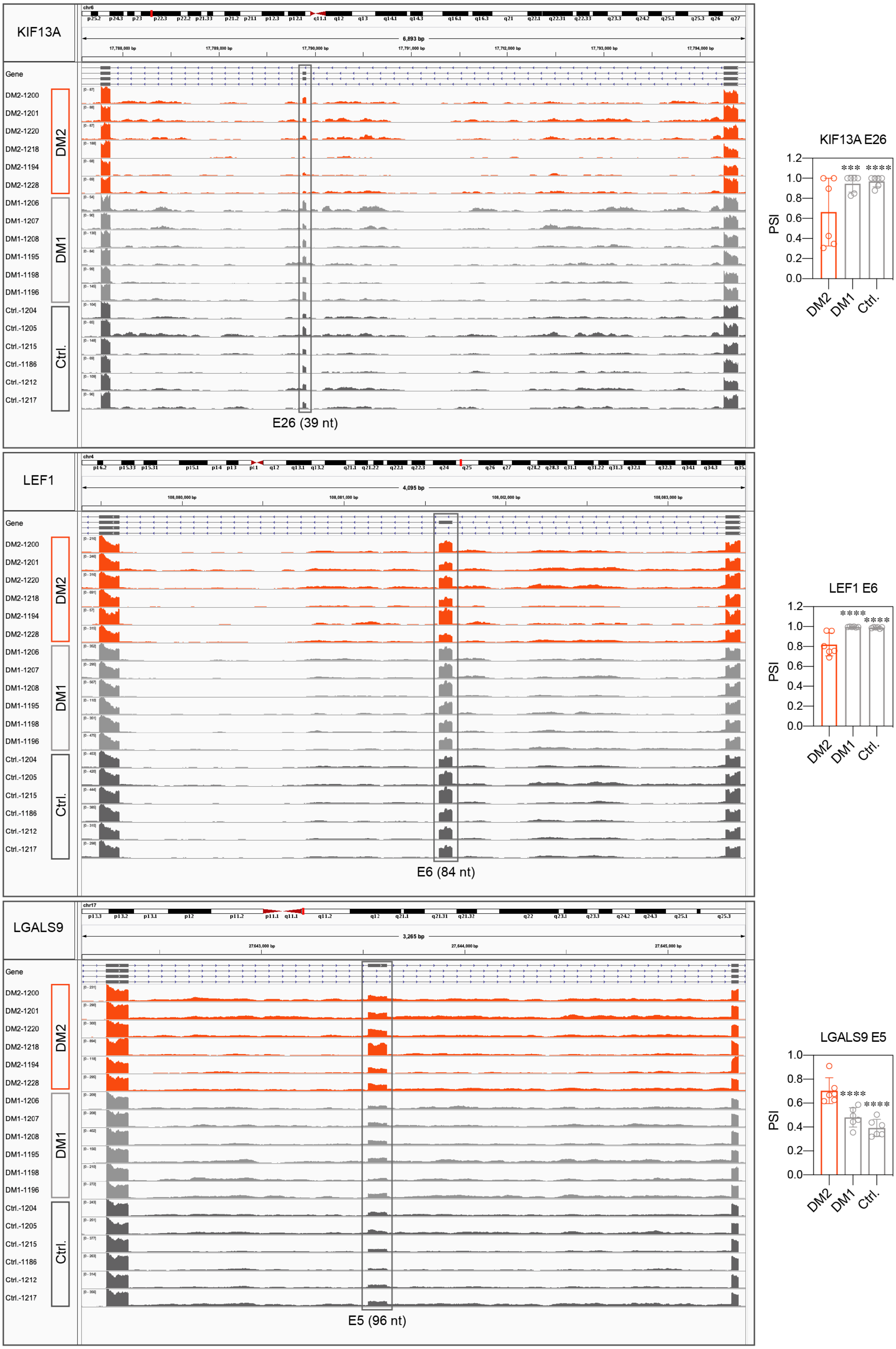

KIF13A
Gene
DM2-1200
DM2-1201
DM2-1220
DM2-1218
DM2-1194
DM2-1228
DM1-1206
DM1-1207
DM1-1208
DM1-1195
DM1-1198
DM1-1196
Ctrl.-1204
Ctrl.-1205
Ctrl.-1215
Ctrl.-1186
Ctrl.-1212
Ctrl.-1217
DM2
DM1
Ctrl.
E26 (39 nt)
KIF13A E26
PSI
1.2
1.0
0.8
0.6
0.4
0.2
0.0
***
****
DM2
DM1
Ctrl.
LEF1
Gene
DM2-1200
DM2-1201
DM2-1220
DM2-1218
DM2-1194
DM2-1228
DM1-1206
DM1-1207
DM1-1208
DM1-1195
DM1-1198
DM1-1196
Ctrl.-1204
Ctrl.-1205
Ctrl.-1215
Ctrl.-1186
Ctrl.-1212
Ctrl.-1217
DM2
DM1
Ctrl.
E6 (84 nt)
LEF1 E6
PSI
1.2
1.0
0.8
0.6
0.4
0.2
0.0
****
****
DM2
DM1
Ctrl.
LGALS9
Gene
DM2-1200
DM2-1201
DM2-1220
DM2-1218
DM2-1194
DM2-1228
DM1-1206
DM1-1207
DM1-1208
DM1-1195
DM1-1198
DM1-1196
Ctrl.-1204
Ctrl.-1205
Ctrl.-1215
Ctrl.-1186
Ctrl.-1212
Ctrl.-1217
DM2
DM1
Ctrl.
E5 (96 nt)
LGALS9 E5
PSI
1.0
0.8
0.6
0.4
0.2
0.0
****
****
DM2
DM1
Ctrl.

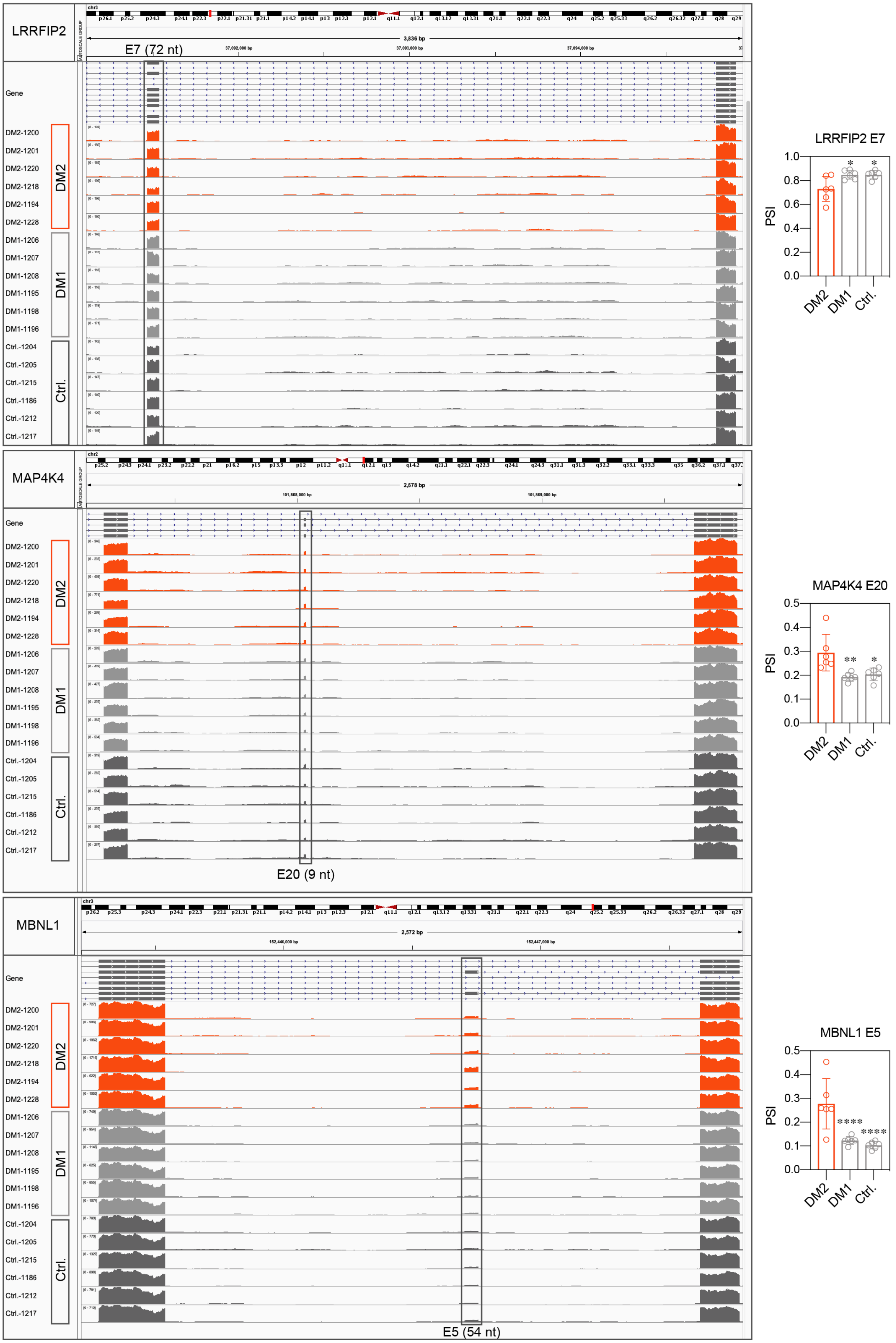

LRRFIP2
E7 (72 nt)
Gene
DM2-1200
DM2-1201
DM2-1220
DM2-1218
DM2-1194
DM2-1228
DM1-1206
DM1-1207
DM1-1208
DM1-1195
DM1-1198
DM1-1196
Ctrl.-1204
Ctrl.-1205
Ctrl.-1215
Ctrl.-1186
Ctrl.-1212
Ctrl.-1217
DM2
DM1
Ctrl.
LRRFIP2 E7
PSI
1.0
0.8
0.6
0.4
0.2
0.0
*
*
DM2
DM1
Ctrl.
MAP4K4
E20 (9 nt)
MAP4K4 E20
PSI
0.5
0.4
0.3
0.2
0.1
0.0
**
*
MBNL1
E5 (54 nt)
MBNL1 E5
PSI
0.5
0.4
0.3
0.2
0.1
0.0
****
****

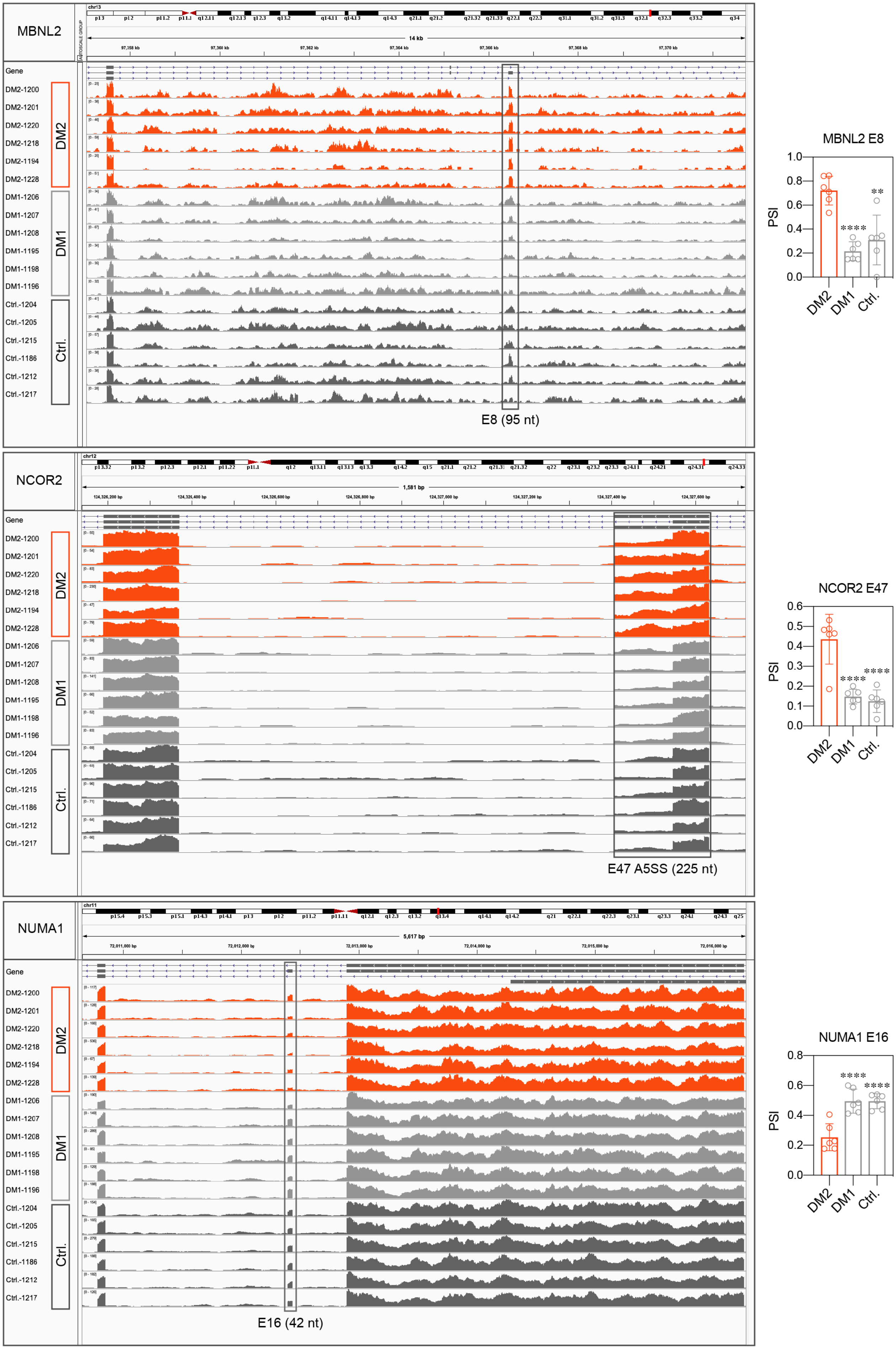
MBNL2
Gene
DM2-1200
DM2-1201
DM2-1220
DM2-1218
DM2-1194
DM2-1228
DM1-1206
DM1-1207
DM1-1208
DM1-1195
DM1-1198
DM1-1196
Ctrl.-1204
Ctrl.-1205
Ctrl.-1215
Ctrl.-1186
Ctrl.-1212
Ctrl.-1217
DM2
DM1
Ctrl.
E8 (95 nt)
MBNL2 E8
PSI
1.0
0.8
0.6
0.4
0.2
0.0
****
**
NCOR2
E47 A5SS (225 nt)
NCOR2 E47
0.6
0.5
0.4
0.3
0.2
0.1
0.0
NUMA1
E16 (42 nt)
NUMA1 E16

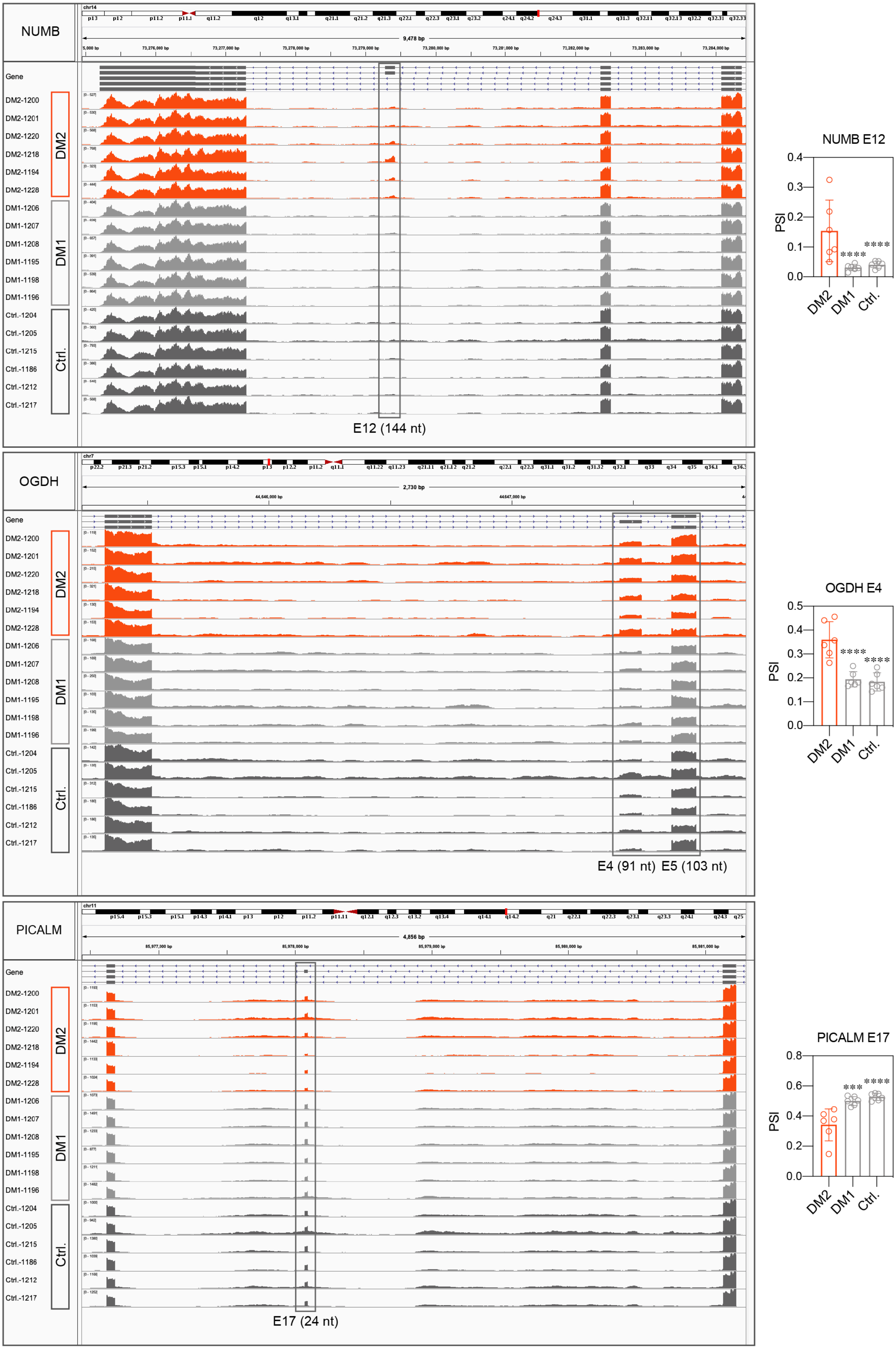
NUMB
E12 (144 nt)
NUMB E12
PSI
DM2
DM1
Ctrl.
OGDH
E4 (91 nt)
E5 (103 nt)
OGDH E4
PICALM
E17 (24 nt)
PICALM E17
Gene
DM2-1200
DM2-1201
DM2-1220
DM2-1218
DM2-1194
DM2-1228
DM1-1206
DM1-1207
DM1-1208
DM1-1195
DM1-1198
DM1-1196
Ctrl.-1204
Ctrl.-1205
Ctrl.-1215
Ctrl.-1186
Ctrl.-1212
Ctrl.-1217

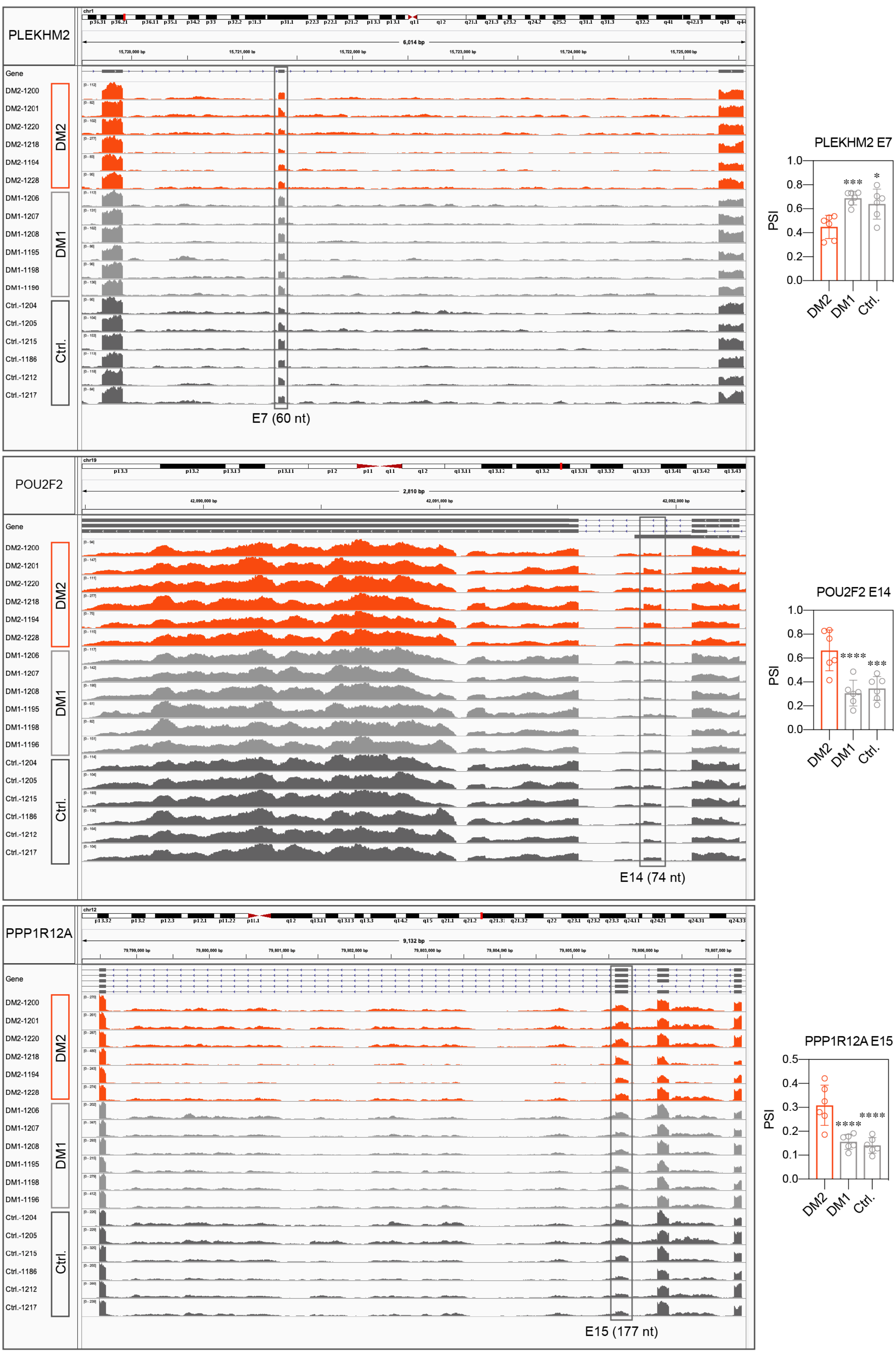

PLEKHM2
Gene
DM2-1200
DM2-1201
DM2-1220
DM2-1218
DM2-1194
DM2-1228
DM1-1206
DM1-1207
DM1-1208
DM1-1195
DM1-1198
DM1-1196
Ctrl.-1204
Ctrl.-1205
Ctrl.-1215
Ctrl.-1186
Ctrl.-1212
Ctrl.-1217
DM2
DM1
Ctrl.
E7 (60 nt)
PLEKHM2 E7
PSI
1.0
0.8
0.6
0.4
0.2
0.0
***
*
DM2
DM1
Ctrl.
POU2F2
Gene
E14 (74 nt)
POU2F2 E14
****
***
PPP1R12A
Gene
E15 (177 nt)
PPP1R12A E15
0.5
0.4
0.3
0.2
0.1
0.0
****
****

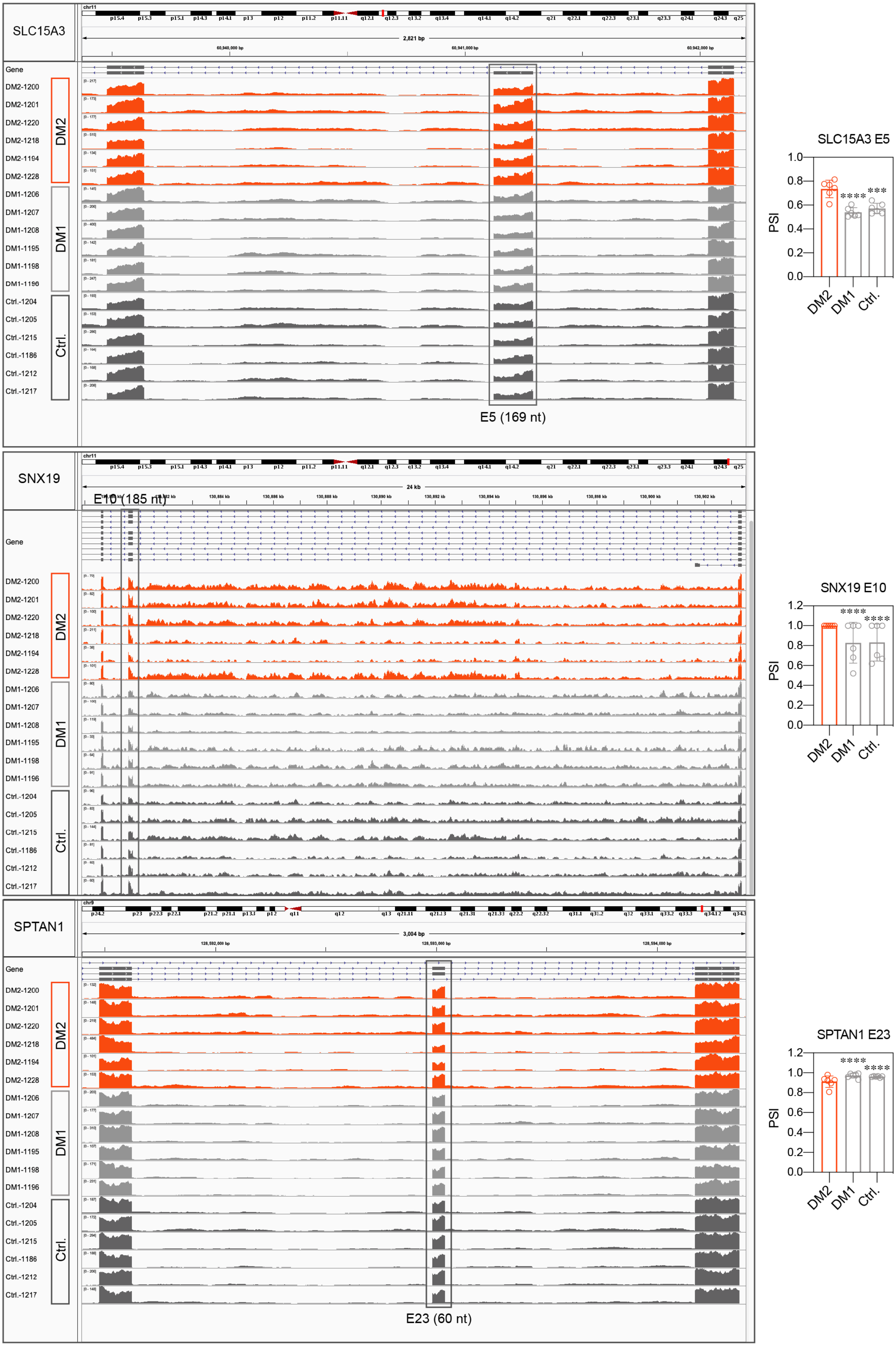
SLC15A3
Gene
DM2-1200
DM2-1201
DM2-1220
DM2-1218
DM2-1194
DM2-1228
DM1-1206
DM1-1207
DM1-1208
DM1-1195
DM1-1198
DM1-1196
Ctrl.-1204
Ctrl.-1205
Ctrl.-1215
Ctrl.-1186
Ctrl.-1212
Ctrl.-1217
DM2
DM1
Ctrl.
E5 (169 nt)
SLC15A3 E5
PSI
****
***
SNX19
E10 (185 nt)
SNX19 E10
SPTAN1
E23 (60 nt)
SPTAN1 E23

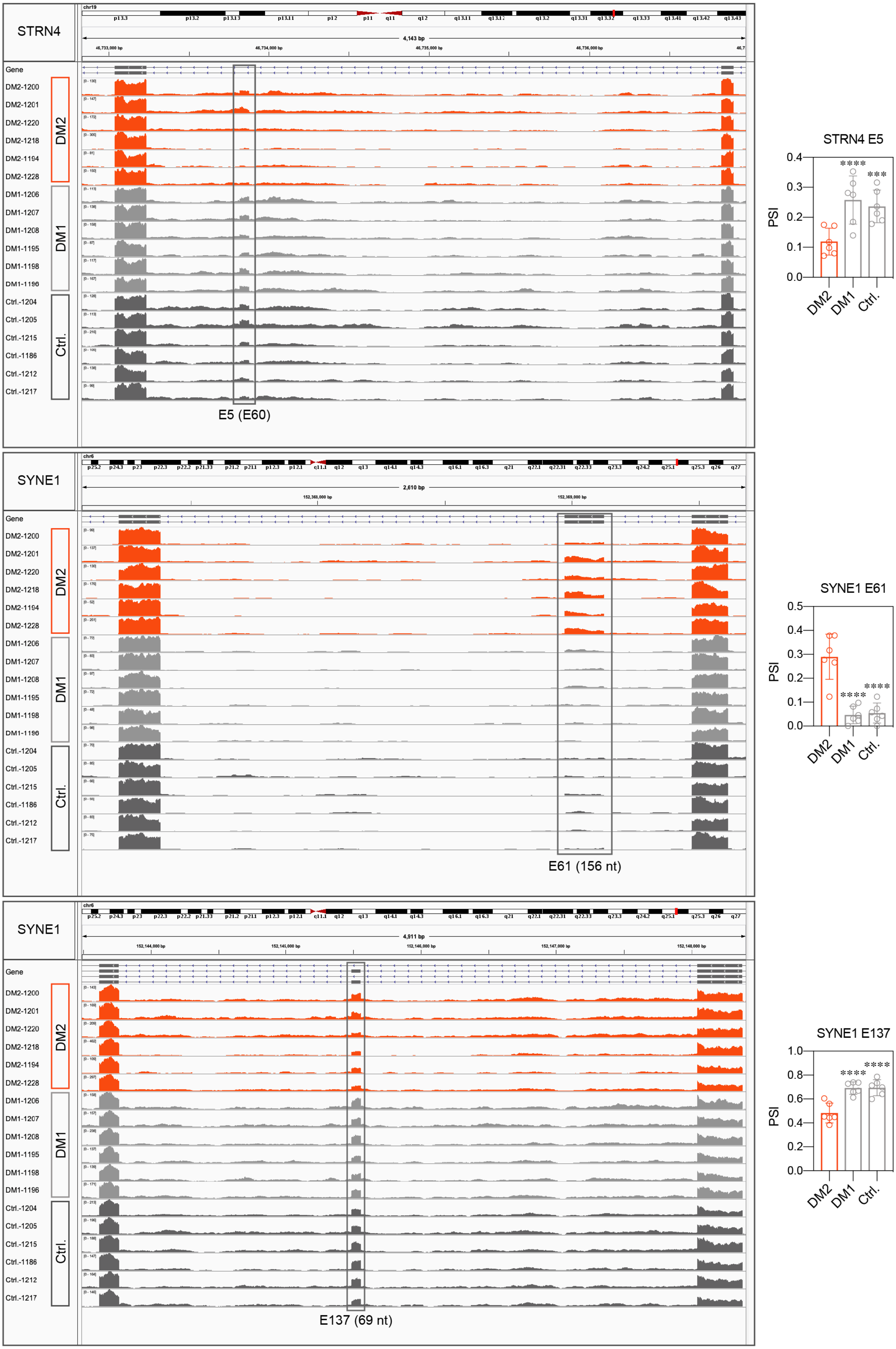
STRN4
Gene
DM2-1200
DM2-1201
DM2-1220
DM2-1218
DM2-1194
DM2-1228
DM1-1206
DM1-1207
DM1-1208
DM1-1195
DM1-1198
DM1-1196
Ctrl.-1204
Ctrl.-1205
Ctrl.-1215
Ctrl.-1186
Ctrl.-1212
Ctrl.-1217
DM2
DM1
Ctrl.
E5 (E60)
STRN4 E5
PSI
SYNE1
E61 (156 nt)
SYNE1 E61
SYNE1
E137 (69 nt)
SYNE1 E137

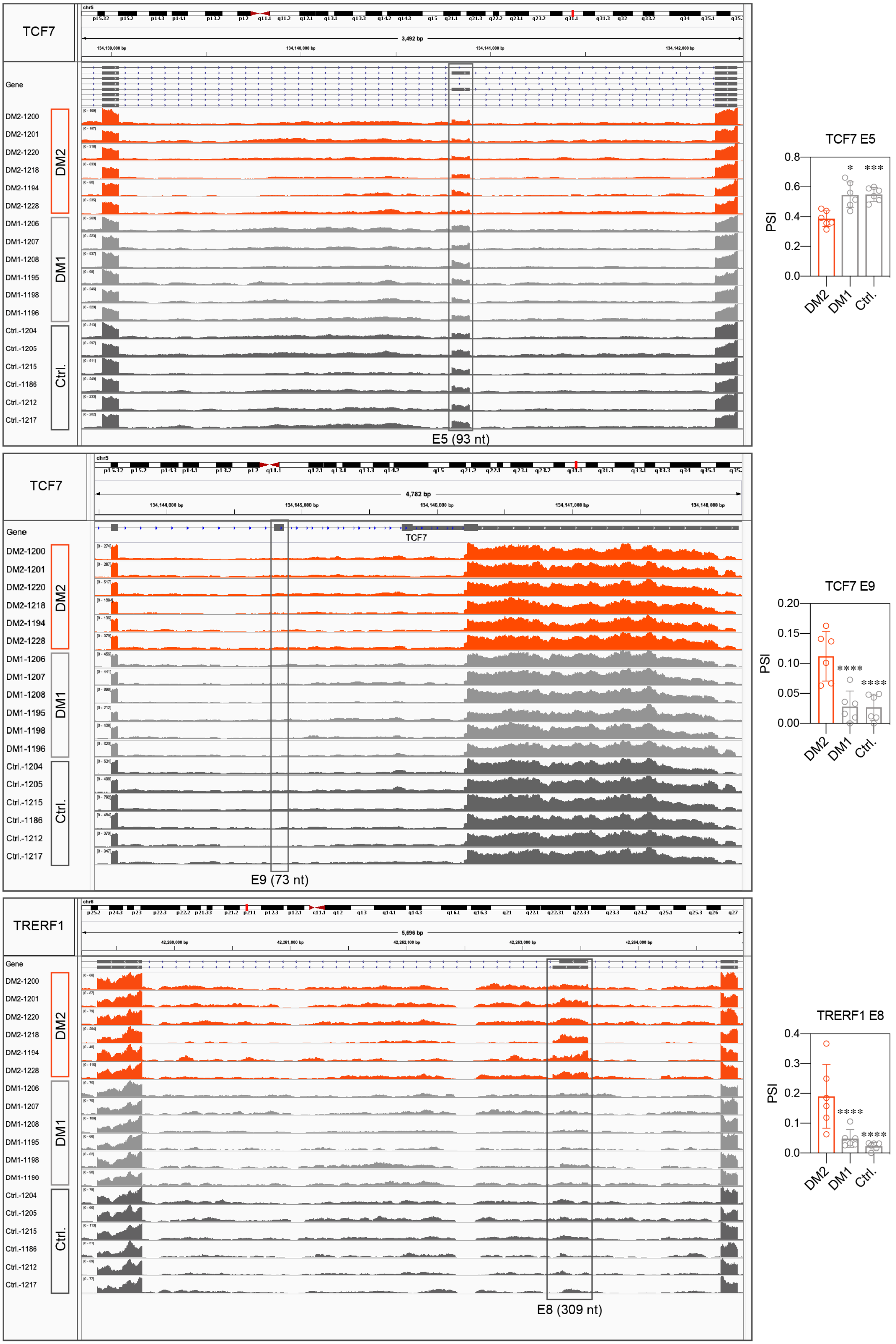
TCF7
chr5
3,492 bp
Gene
DM2-1200
DM2-1201
DM2-1220
DM2-1218
DM2-1194
DM2-1228
DM1-1206
DM1-1207
DM1-1208
DM1-1195
DM1-1198
DM1-1196
Ctrl.-1204
Ctrl.-1205
Ctrl.-1215
Ctrl.-1186
Ctrl.-1212
Ctrl.-1217
DM2
DM1
Ctrl.
E5 (93 nt)
TCF7 E5
PSI
0.8
0.6
0.4
0.2
0.0
*
***
DM2
DM1
Ctrl.
TCF7
chr5
4,782 bp
Gene
TCF7
DM2-1200
DM2-1201
DM2-1220
DM2-1218
DM2-1194
DM2-1228
DM1-1206
DM1-1207
DM1-1208
DM1-1195
DM1-1198
DM1-1196
Ctrl.-1204
Ctrl.-1205
Ctrl.-1215
Ctrl.-1186
Ctrl.-1212
Ctrl.-1217
DM2
DM1
Ctrl.
E9 (73 nt)
TCF7 E9
PSI
0.20
0.15
0.10
0.05
0.00
****
****
DM2
DM1
Ctrl.
TRERF1
chr6
5,696 bp
Gene
DM2-1200
DM2-1201
DM2-1220
DM2-1218
DM2-1194
DM2-1228
DM1-1206
DM1-1207
DM1-1208
DM1-1195
DM1-1198
DM1-1196
Ctrl.-1204
Ctrl.-1205
Ctrl.-1215
Ctrl.-1186
Ctrl.-1212
Ctrl.-1217
DM2
DM1
Ctrl.
E8 (309 nt)
TRERF1 E8
PSI
0.4
0.3
0.2
0.1
0.0
****
****
DM2
DM1
Ctrl.

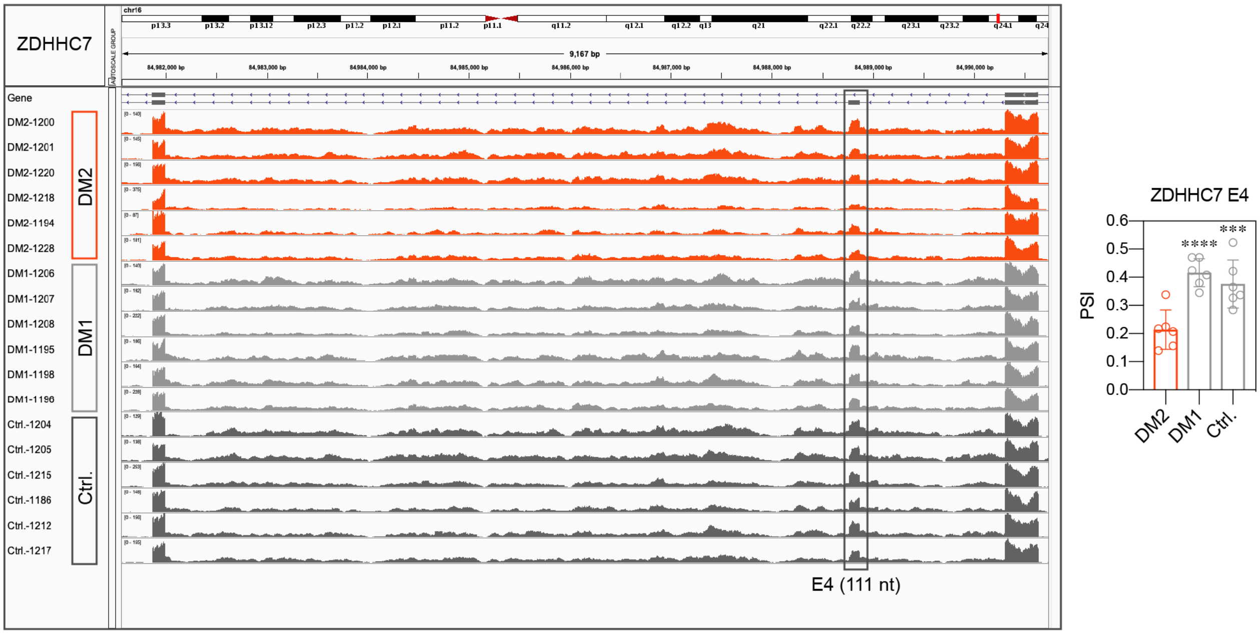

Supplementary Fig. 8. Genome browser views of selected human alternative splicing events

Genome browser view of selected AS events from DM2 (n = 6), DM1 (n = 6) and unaffected control (Ctrl.; n = 6) whole blood RNA-seq data. DM2, DM1 and Ctrl. are represented by orange, light grey and dark gray wiggle plots, respectively. Bar graphs show mean PSI ± SD. rMATS; * FDR < 0.05, ** FDR < 0.01, *** FDR < 0.001, **** FDR < 0.0001.

Source data are provided as a Supplementary Data 1 file.

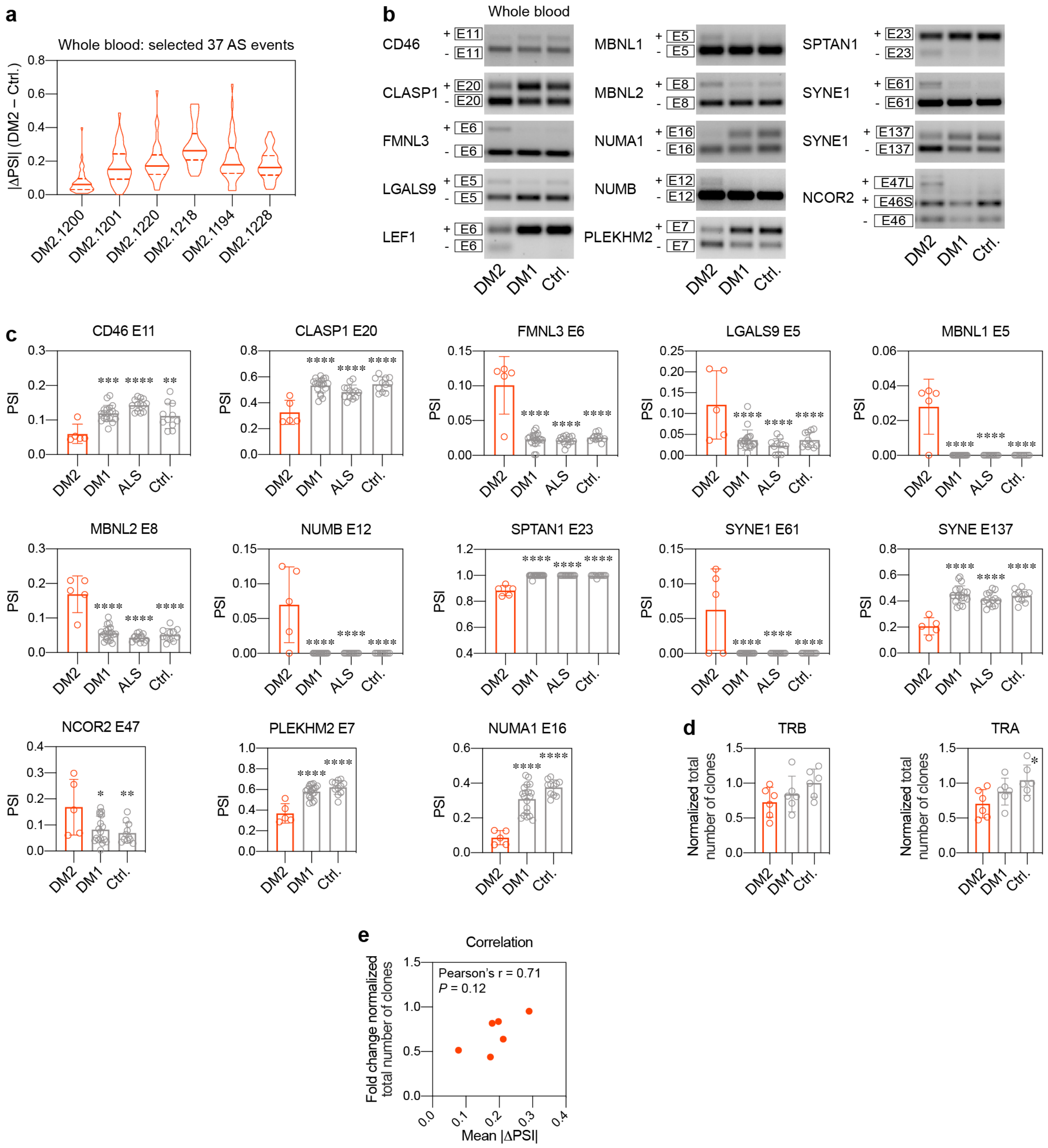

Supplementary Fig. 9. Alternative splicing changes in DM2 whole blood

a Six DM2 whole blood samples representing different levels of spliceopathy detected. Violin plot represents the distribution of |ΔPSI| values for 37 selected AS events. Center line represents the median, and the dash lines extend from the 25th to 75th percentiles.

b Representative gels of 14 AS event RT-PCR assays on DM2, DM1 and unaffected control (Ctrl.) whole blood samples.

c Aberrant AS events in a large cohort of DM2 (n = 5), DM1 (n = 19), ALS = (n = 13) and unaffected (n = 11) control whole blood samples. Bar graphs show mean PSI ± SD. Significant difference were determined by Dunnett's multiple comparison test: * $P_{adj} < 0.05$, ** $P_{adj} < 0.01$, *** $P_{adj} < 0.001$, **** $P_{adj} < 0.0001$.

d Aberrant TCR repertoire detected in whole blood RNA-seq. Bar graphs show mean total number of clones normalized to uniquely mapped reads ± SD. Significant difference were determined by Dunnett's multiple comparison test: * $P_{adj} = 0.022$.

e Correlation between fold change TCR repertoire and spliceopathy in DM2 (n = 6) whole blood RNA-seq. TCR repertoire represents fold change normalized total number of TRB and TRA clones. Mean spliceopathy is represented by 37 selected AS events.

Source data are provided as the Source Data and Supplementary Data 1 files.

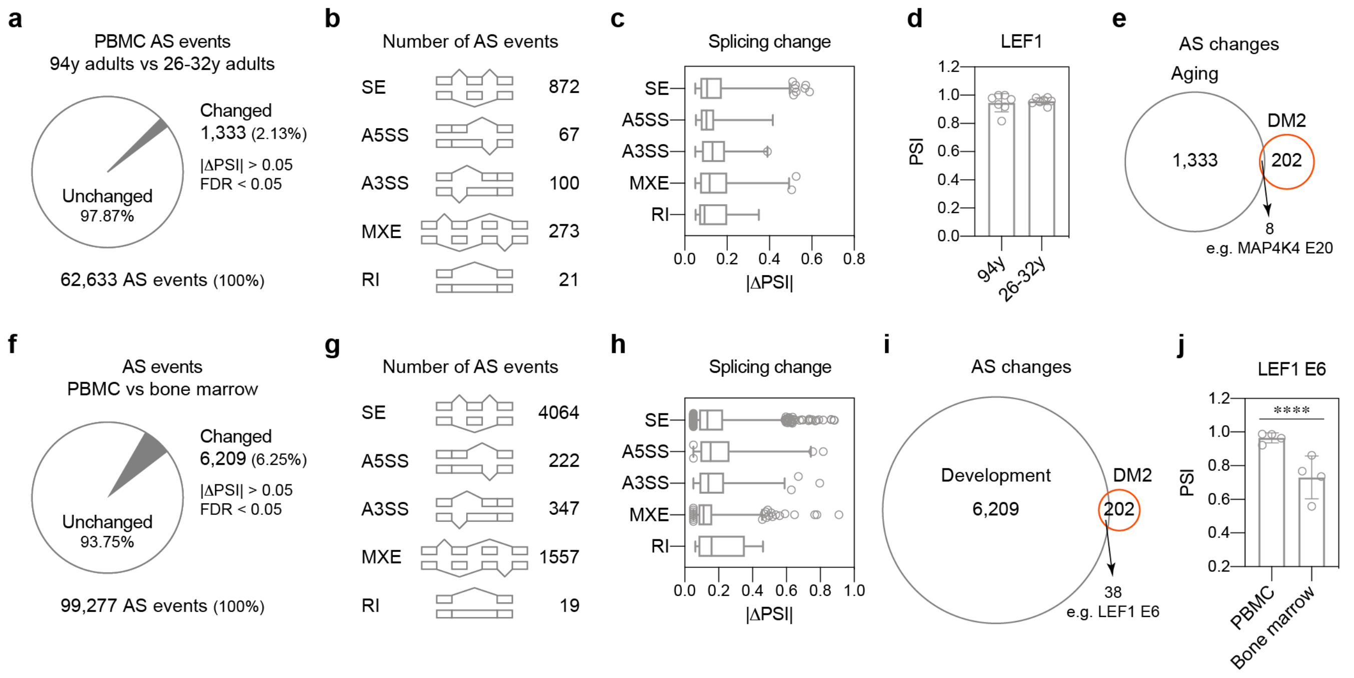

Supplementary Fig. 10. Blood DM2 alternative splicing changes are not age dependent

a Pie chart represents all detected AS events in the PBMC RNA-seq data. Gray triangle represents significantly changed AS events in PBMC obtained from 94 year-old (n = 7) compared to 26-32 year-old females (n = 7) - GSE122309[4].

b Number of significantly changed AS events assigned to different categories.

c Wide range of splicing changes. Box plot of AS event |ΔPSI| values. Center line represents the median, and the box extends from the 25th to 75th percentiles. Whiskers show 1-99 percentile.

d Aging does not influence LEF1 E6 splicing. Bar graph shows mean PSI ± SD. rMATS; FDR > 0.05.

e Venn diagram shows minimal overlap between AS changes during aging (PBMC) and DM2 (whole blood).

f Pie chart represents all detected AS events in the PBMC RNA-seq data. Gray triangle represents significantly changed AS events in PBMCs (n = 4) compared to bone marrow (n = 4) - GSE58335 and GSE61410[3].

g Number of significantly changed AS events assigned to different categories.

h Wide range of splicing changes. Box plot of AS event |ΔPSI| values. Center line represents the median, and the box extends from the 25th to 75th percentiles. Whiskers show 1-99 percentile.

i Venn diagram shows overlap between AS changes during blood cell development and DM2 (whole blood).

j LEF1 E6 splicing is developmentally regulated. Bar graph shows mean PSI ± SD. rMATS; **** FDR < 0.0001.

Source data are provided as a Supplementary Data 1 file.

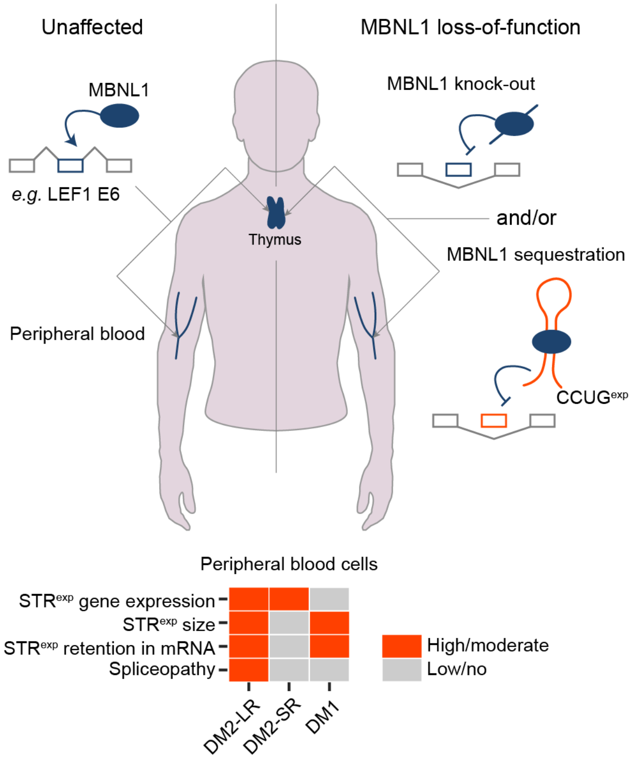

Supplementary Fig. 11. Graphical abstract

MBNL1 depletion leads to pre-mRNA misprocessing in lymphoid organs and cells. A high load of CCUG repeats cause spliceopathy in myotonic dystrophy type 2 peripheral blood cells. STR^{exp} - short tandem repeat expansion; LR - long repeats; SR - short repeats.

Supplementary References

1 Kernfeld, E. M. *et al.* A Single-Cell Transcriptomic Atlas of Thymus Organogenesis Resolves Cell Types and Developmental Maturation. *Immunity* **48**, 1258-1270.e1256, doi:10.1016/j.immuni.2018.04.015 (2018).

2 Bagger, F. O., Kinalis, S. & Rapin, N. BloodSpot: a database of healthy and malignant haematopoiesis updated with purified and single cell mRNA sequencing profiles. *Nucleic Acids Res* **47**, D881-D885, doi:10.1093/nar/gky1076 (2019).

3 Dvinge, H. *et al.* Sample processing obscures cancer-specific alterations in leukemic transcriptomes. *Proc Natl Acad Sci U S A* **111**, 16802-16807, doi:10.1073/pnas.1413374111 (2014).

4 Nevalainen, T. *et al.* Aging-associated patterns in the expression of human endogenous retroviruses. *PLoS One* **13**, e0207407, doi:10.1371/journal.pone.0207407 (2018).
