# Supplementary material for: Loss of MBNL1 induces RNA misprocessing in the thymus and peripheral blood
Source: Nat Commun. 2020 Apr 24;11:2022. doi: 10.1038/s41467-020-15962-x (PMC7181699; doi:10.1038/s41467-020-15962-x)
Supplement: Supplementary file 3 — Reporting Summary [file 41467_2020_15962_MOESM3_ESM.pdf]

nature research

Corresponding author(s): Łukasz J. Sznajder and Maurice S. Swanson

Last updated by author(s): 03/03/2020

Reporting Summary

Nature Research wishes to improve the reproducibility of the work that we publish. This form provides structure for consistency and transparency in reporting. For further information on Nature Research policies, see Authors & Referees and the Editorial Policy Checklist.

Statistics

For all statistical analyses, confirm that the following items are present in the figure legend, table legend, main text, or Methods section.

n/a	Confirmed	
☐	☒	The exact sample size (*n*) for each experimental group/condition, given as a discrete number and unit of measurement
☐	☒	A statement on whether measurements were taken from distinct samples or whether the same sample was measured repeatedly
☐	☒	The statistical test(s) used AND whether they are one- or two-sided<br>*Only common tests should be described solely by name; describe more complex techniques in the Methods section.*
☐	☒	A description of all covariates tested
☐	☒	A description of any assumptions or corrections, such as tests of normality and adjustment for multiple comparisons
☐	☒	A full description of the statistical parameters including central tendency (e.g. means) or other basic estimates (e.g. regression coefficient) AND variation (e.g. standard deviation) or associated estimates of uncertainty (e.g. confidence intervals)
☐	☒	For null hypothesis testing, the test statistic (e.g. *F*, *t*, *r*) with confidence intervals, effect sizes, degrees of freedom and *P* value noted<br>*Give P values as exact values whenever suitable.*
☒	☐	For Bayesian analysis, information on the choice of priors and Markov chain Monte Carlo settings
☒	☐	For hierarchical and complex designs, identification of the appropriate level for tests and full reporting of outcomes
☐	☒	Estimates of effect sizes (e.g. Cohen's *d*, Pearson's *r*), indicating how they were calculated

Our web collection on statistics for biologists contains articles on many of the points above.

Software and code

Policy information about availability of computer code

Data collection	Basecalling was done using Illumina Real-Time Analysis (RTA) and demultiplexed fastq files were obtained from BaseSpace with BaseMount. Scanned histological tissue sections were processed with Aperio ImageScope software.
Data analysis	STAR was used to map reads to the reference genome (mm10 or hg38). Salmon and DEseq2 were used to quantify and normalize gene expression followed by differential analysis. MiXCR was used for repertoire analysis. rMATS turbo was used for differential alternative splicing. IRFinder was used for retained intron quantification. Flow cytometry data were analyzed using FlowJo Data Analysis software (Tree Star). RT-PCR results were analyzed using Multi Gauge software (FujiFilm) or Image Lab (BioRad). Statistical analysis was performed using GraphPad Prism (Version 8) software.

For manuscripts utilizing custom algorithms or software that are central to the research but not yet described in published literature, software must be made available to editors/reviewers. We strongly encourage code deposition in a community repository (e.g. GitHub). See the Nature Research guidelines for submitting code & software for further information.

Data

Policy information about availability of data

All manuscripts must include a data availability statement. This statement should provide the following information, where applicable:

- Accession codes, unique identifiers, or web links for publicly available datasets
- A list of figures that have associated raw data
- A description of any restrictions on data availability

The GEO accession numbers for RNA-seq data are listed in Supplementary Data. RNA-seq data generated in this study have been deposited in GEO under accession GSE138691. Data used for graph generation are in Supplementary Data.

Field-specific reporting

Please select the one below that is the best fit for your research. If you are not sure, read the appropriate sections before making your selection.

☒ Life sciences ☐ Behavioural & social sciences ☐ Ecological, evolutionary & environmental sciences

For a reference copy of the document with all sections, see nature.com/documents/nr-reporting-summary-flat.pdf

Life sciences study design

All studies must disclose on these points even when the disclosure is negative.

Sample size	Sample size selection for RNA-seq experiments was supported by the power analysis. For human alternative splicing RT-PCR validations we used all samples available to us. For other experiments sample size was determined based on experiment type and our previous studies or preliminary data.
Data exclusions	Some RT-PCR results for random samples were excluded from the analysis due to very low/no amplification of particular transcripts. Other data were not excluded from the analysis.
Replication	All attempts at replication data included in the manuscript were successful. If attempted, same results were achieved independently by two researchers.
Randomization	In order to avoid batch effects in multiple flow cell RNA-seq experiment, the genotypes were distributed equally on each flow cell. For RT-PCR experiments, the samples were run according to their ID number instead of genotype.
Blinding	For the most of the study investigators were not blinded. Since human samples were deidentified, initial RT-PCR screenings were blinded.

Reporting for specific materials, systems and methods

We require information from authors about some types of materials, experimental systems and methods used in many studies. Here, indicate whether each material, system or method listed is relevant to your study. If you are not sure if a list item applies to your research, read the appropriate section before selecting a response.

Materials & experimental systems

n/a	Involved in the study	
☐	☒	Antibodies
☐	☒	Eukaryotic cell lines
☒	☐	Palaeontology
☐	☒	Animals and other organisms
☐	☒	Human research participants
☒	☐	Clinical data

Methods

n/a	Involved in the study	
☒	☐	ChIP-seq
☐	☒	Flow cytometry
☒	☐	MRI-based neuroimaging

Antibodies

Antibodies used	anti-Mbnl1 A2764 (a gift from Dr. Charles Thornton); anti-Gapdh 6c5 (Abcam; ab8245); anti-rabbit IgG and anti-mouse-IgG conjugated to horseradish peroxidase (GE Healthcare; NA934V and NA931V); anti-CD4 (RM4-5)-PE-Cy7 or -APC; anti-CD8 (53-6.7)-Pacific Blue or -PE-Cy5 (BD Biosciences); anti-CD3-Alexa700 (17A2); anti-CD45/B220-Pacific Blue (RA3-6B2); anti-CD4-PE-Cy7 (RM4-5); anti-CD8-PE-Cy5 (53-6.7).
Validation	anti-Mbnl1 A2764 (DOI: 10.1073/pnas.0604970103)

Eukaryotic cell lines

Policy information about cell lines

Cell line source(s)	ATCC
Authentication	Cell lines from ATCC have been thoroughly tested and authenticated.
Mycoplasma contamination	Cell line has been tested for mycoplasma contamination.
Commonly misidentified lines (See ICLAC register)	*Name any commonly misidentified cell lines used in the study and provide a rationale for their use.*

Animals and other organisms

Policy information about studies involving animals; ARRIVE guidelines recommended for reporting animal research

Laboratory animals	Mouse strains: 129S1-Mbnl1 KO, B6-Mbnl1 KO, B6.129S1-Mbnl1 KO, Mbnl2 KO and Mbnl3 KO, 129S1-wild type, B6-wild type, B6.129S1-wild type. Sex: Males and females. Age: 0-50 weeks of age.
Wild animals	Study did not involve wild animals.
Field-collected samples	Study did not involve samples collected from the field.
Ethics oversight	All animal procedures and endpoints were in accordance with University of Florida approved Institutional Animal Care and Use Committee (IACUC) guidelines and animals were sacrificed in accordance with IACUC approved protocols.

Note that full information on the approval of the study protocol must also be provided in the manuscript.

Human research participants

Policy information about studies involving human research participants

Population characteristics	Genotype/diagnosis: DM2, DM1, sporadic ALS, unaffected controls. Sex: females and males. Age: 19-77 years of age.
Recruitment	Patients were recruited at the University of Florida Department of Neurology clinic as well as during Myotonic Dystrophy Foundation Annual Conferences.
Ethics oversight	Patient blood samples were collected following written informed consent as approved by the Universities of Florida Institutional Review Board (IRB).

Note that full information on the approval of the study protocol must also be provided in the manuscript.

Flow Cytometry

Plots

Confirm that:

- [x] The axis labels state the marker and fluorochrome used (e.g. CD4-FITC).
- [x] The axis scales are clearly visible. Include numbers along axes only for bottom left plot of group (a 'group' is an analysis of identical markers).
- [x] All plots are contour plots with outliers or pseudocolor plots.
- [x] A numerical value for number of cells or percentage (with statistics) is provided.

Methodology

Sample preparation	Thymus and spleen were dissected into ice cold 1× PBS and connective tissue and fat removed. Single cell suspensions were made by mincing tissue through 100 um cell strainers (BD Biosciences) into 1× PBS, pH 7.4. Cells were pelleted 400 × g, 10 minutes, room temperature (RT) and resuspended in 150 mM ammonium chloride, 1 mM potassium bicarbonate, 0.01 mM EDTA, pH 7.2 (Thermo Fisher) for red blood cell lysis. Cells were resuspended in RPMI-1640 (Mediatech) supplemented with 10% fetal bovine serum (FBS) (cRPMI; Hyclone).
Instrument	Becton Dickinson LSR II
Software	BD FACSDiva was used at collection. Final analysis utilized FlowJo 7.6 software.
Cell population abundance	Cells were not sorted for the purposes of these experiments. Cells were gated on side scatter and foward scatter to remove debris from analysis. A minimum of 1 million cells was used in each flow sample, recording 10,000-20,000 events. Gating from debris included ≥ 90% of collected events in subsequent gating strategies and analysis.
Gating strategy	Thymocytes and splenocytes were gated away from debris in SSC and FSC plots based on size. Subsequent gates of the thymocyte populations for either CD4/CD8 staining or AnnexinV-FITC/PI apoptosis staining were completed based on single-dye control samples. Splenocyte gates for CD3/CD45(B220) and CD4/CD8 staining were completed based on single-dye controls.

- [x] Tick this box to confirm that a figure exemplifying the gating strategy is provided in the Supplementary Information.
