# Supplementary material for: Loss of MBNL1 induces RNA misprocessing in the thymus and peripheral blood
Source: Nat Commun. 2020 Apr 24;11:2022. doi: 10.1038/s41467-020-15962-x (PMC7181699; doi:10.1038/s41467-020-15962-x)
Supplement: Supplementary file 4 — Description of Additional Supplementary Files [file 41467_2020_15962_MOESM4_ESM.pdf]

Description of Additional Supplementary Files

File Name: Supplementary Data 1
Description: Supplementary Data 1 file contains primer and siRNA sequences used in this study, detailed information about RNA-seq samples, and all results used for graph generation in the main text as well as the Supplementary Information file.
