# Supplementary material for: Loss of MBNL1 induces RNA misprocessing in the thymus and peripheral blood
Source: Nat Commun. 2020 Apr 24;11:2022. doi: 10.1038/s41467-020-15962-x (PMC7181699; doi:10.1038/s41467-020-15962-x)
Supplement: Supplementary file 6 — Source Data [file 41467_2020_15962_MOESM6_ESM.pdf]

Source File

Loss of MBNL1 induces RNA mis-processing in the thymus and peripheral blood

Ł. J. Sznajder et al.

Fig. 3g

Lef1 E6

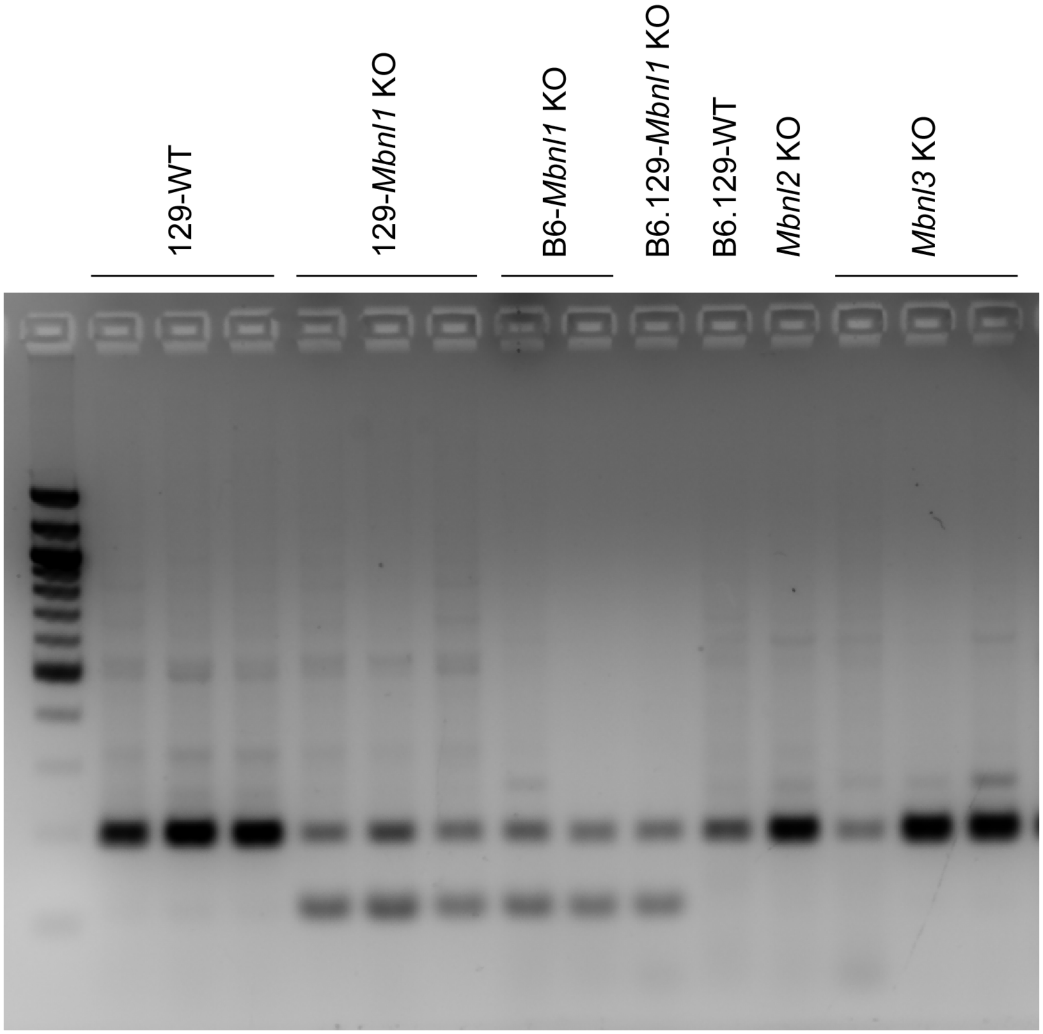

Lef1: 205 bp (+E6); 121 bp (-E6)

Fig. 4a

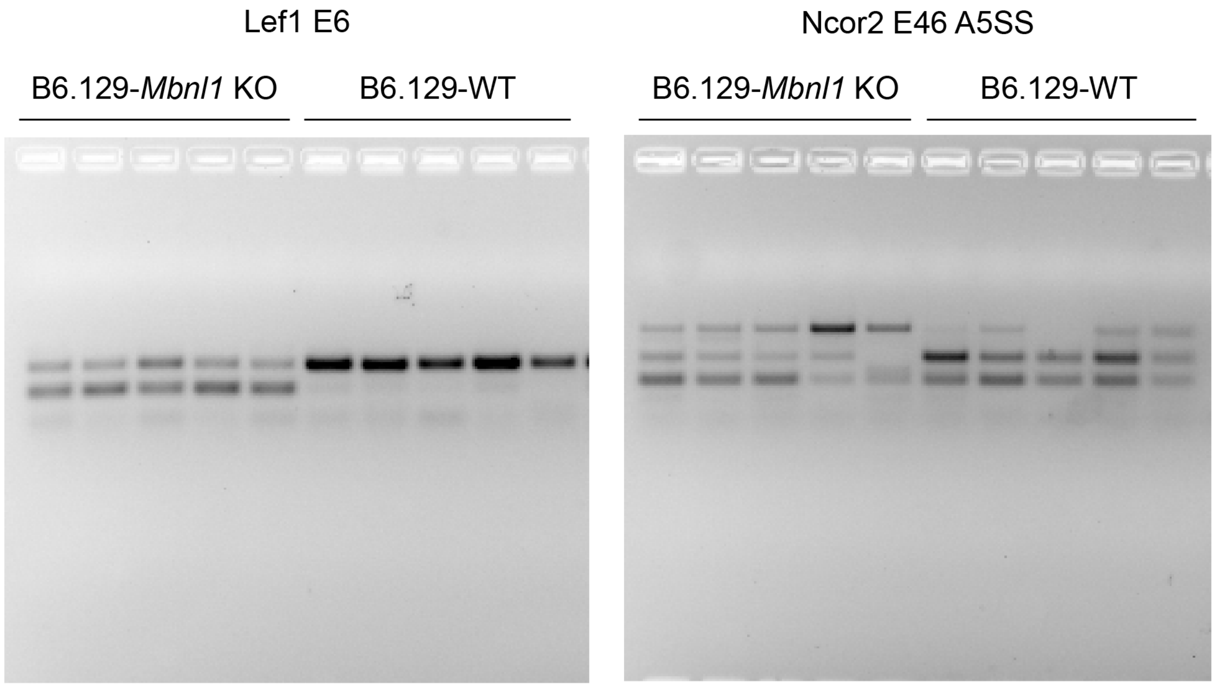

Lef1: 205 bp (+E6); 121 bp (-E6)
Ncor2: 368 bp (+E46L); 227 bp (+E46S); 140 bp (-E46)

Fig. 5a

CNBP-IR

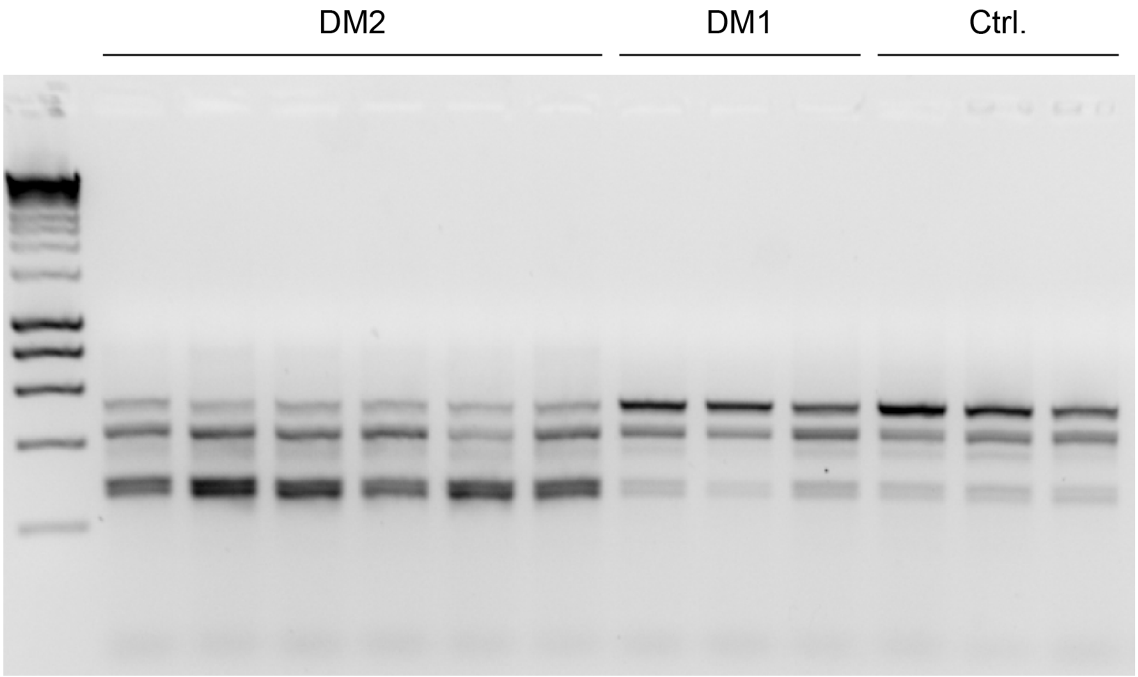

CNBP-IR: 553 bp (pre-mRNA; I1-E2-I2-E3-I3-E4);
458 bp (intermediate; I1-E2-E3-I3-E4); 296 bp (CNBP-IR; I1-E2-E3-E4)

Fig. 5g

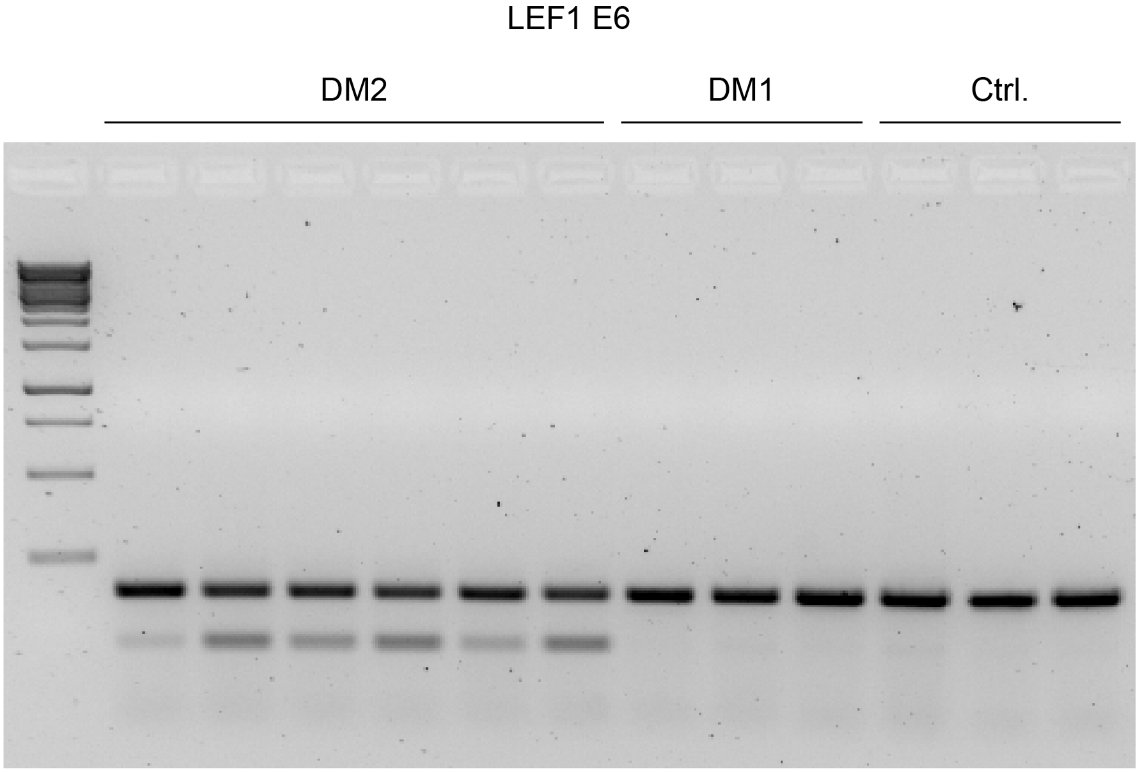

LEF1: 185 bp (+E6); 101 bp (-E6)

Supplementary Fig. 1c

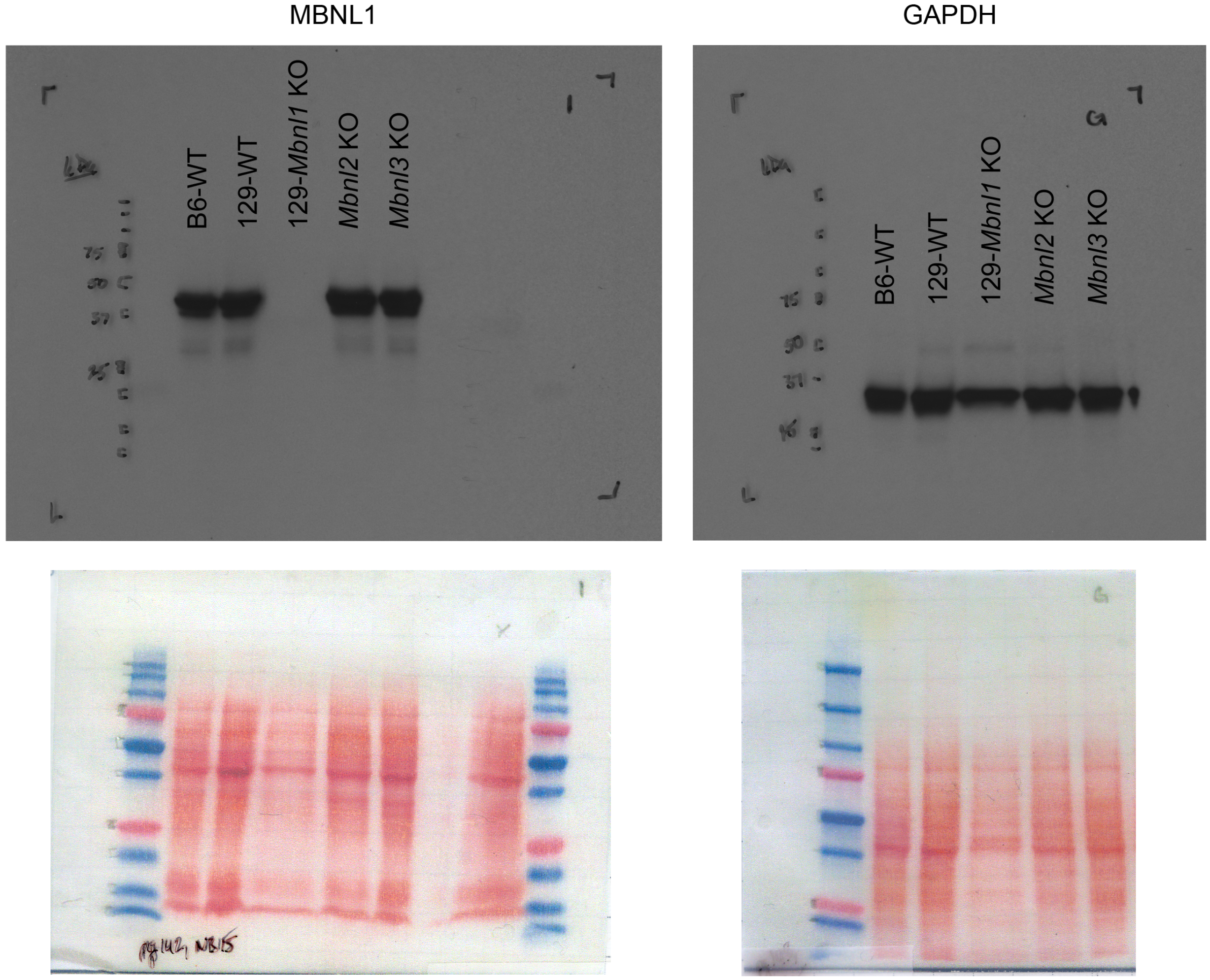

Primary antibodies: anti-MBNL1 A2764 (a gift from Dr. Charles Thornton, 1:5,000) and anti-GAPDH 6c5 (Abcam CN: ab8245, 1:10,000).

Supplementary Fig. 4b

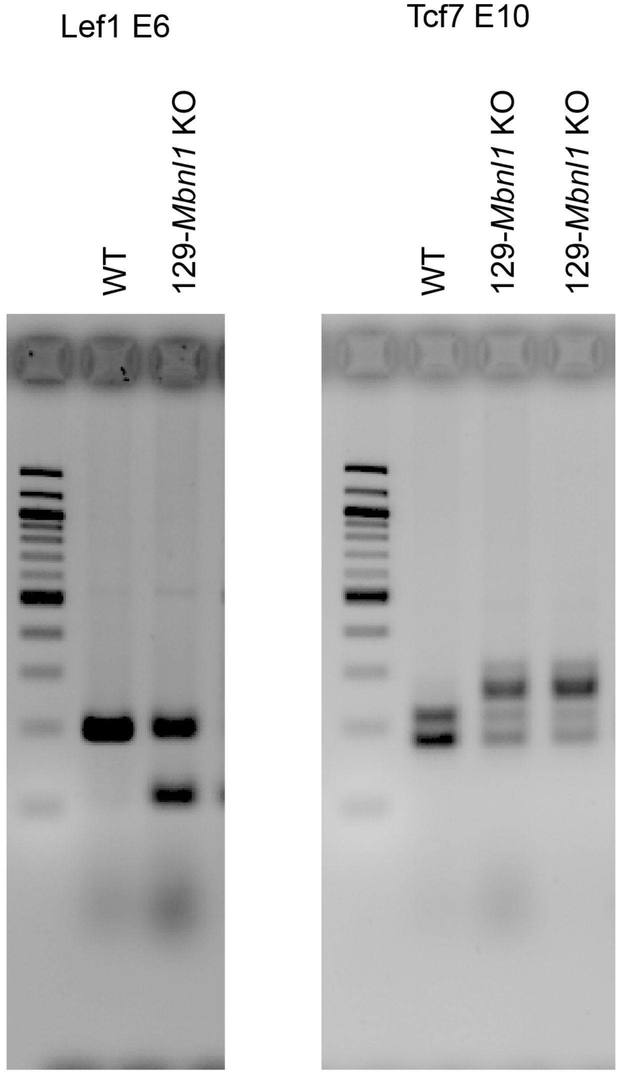

Lef1: 205 bp (+E6); 121 bp (-E6)
Tcf7: 277 bp and 254 bp (+E10); 204 bp and 181 bp (-E10)

Supplementary Fig. 4d

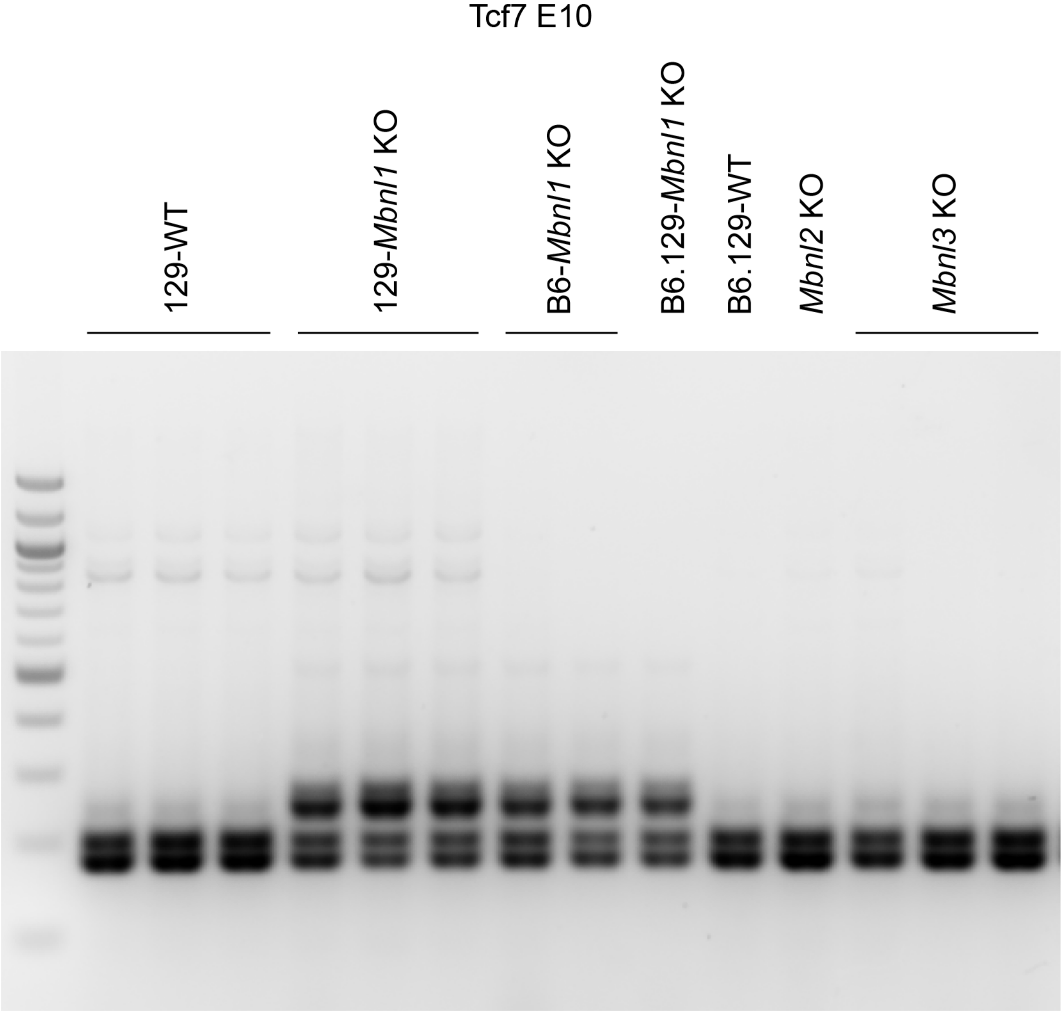

Tcf7: 277 bp and 254 bp (+E10); 204 bp and 181 bp (-E10)

Supplementary Fig. 4e

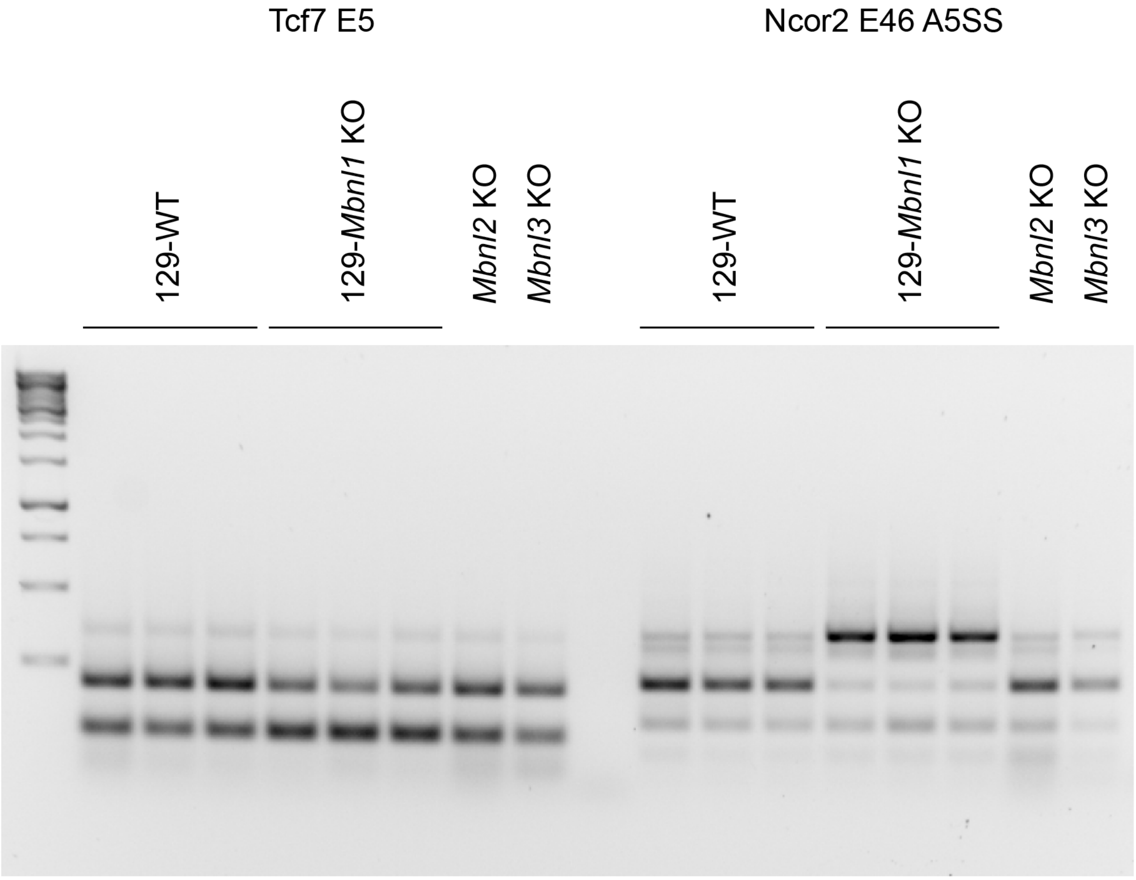

Tcf7: 206 bp (+E5); 113 bp (-E5)
Ncor2: 368 bp (+E46L); 227 bp (+E46S); 140 bp (-E46)

Supplementary Fig. 4e

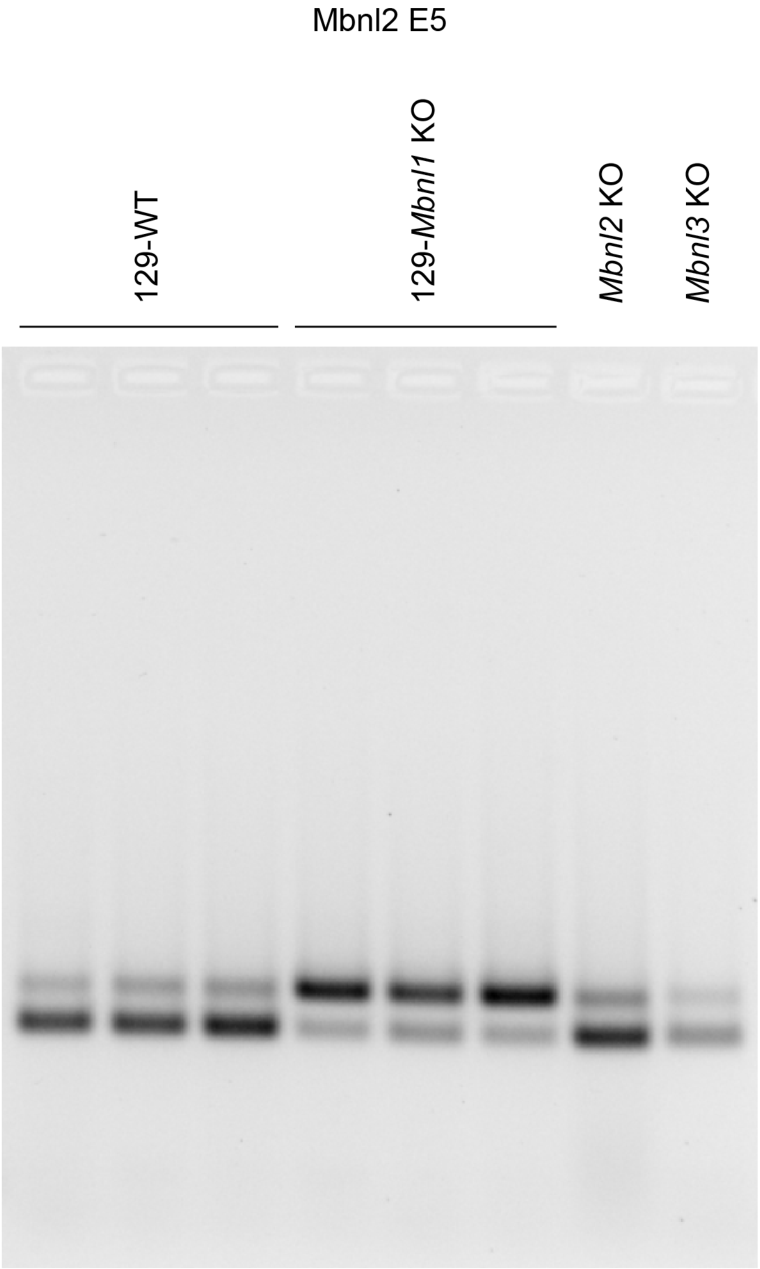

Mbnl2: 255 bp (+E5); 201 bp (-E5)

Supplementary Fig. 4i

LEF1 E6

siCtrl. siMBNL1 siMBNL1/2

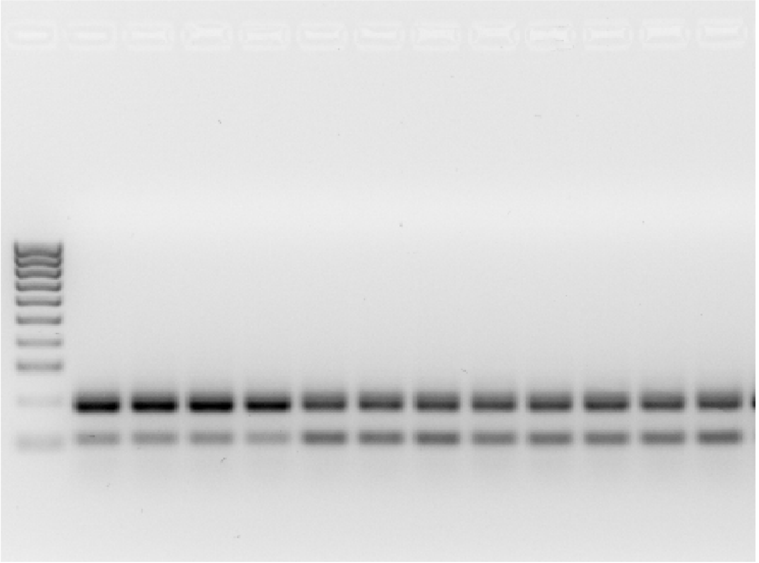

LEF1: 185 bp (+E6); 101 bp (-E6)

Supplementary Fig. 4i

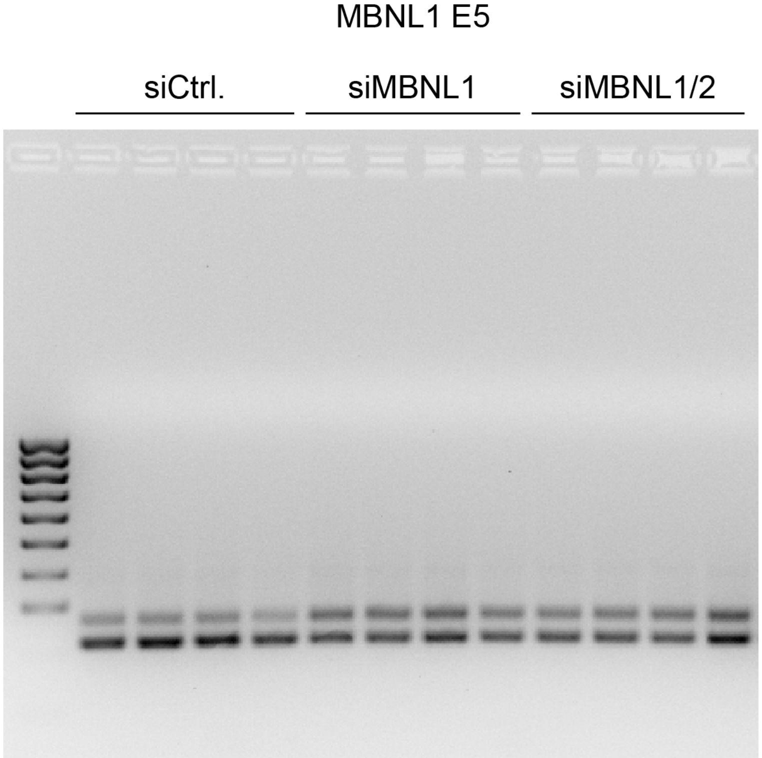

MBNL1: 270 bp (+E10); 216 bp (-E10)

Supplementary Fig. 4i

FMNL3 E6

siCtrl. siMBNL1 siMBNL1/2

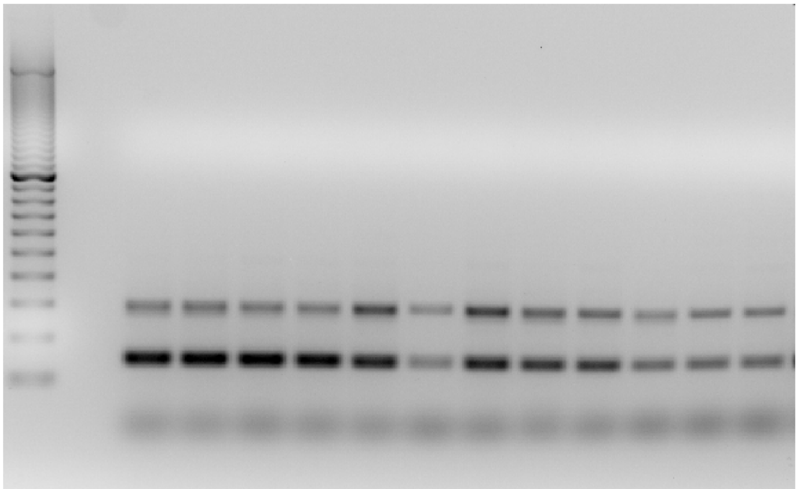

FMNL3: 303 bp (+E6); 150 bp (-E6)

Supplementary Fig. 4i

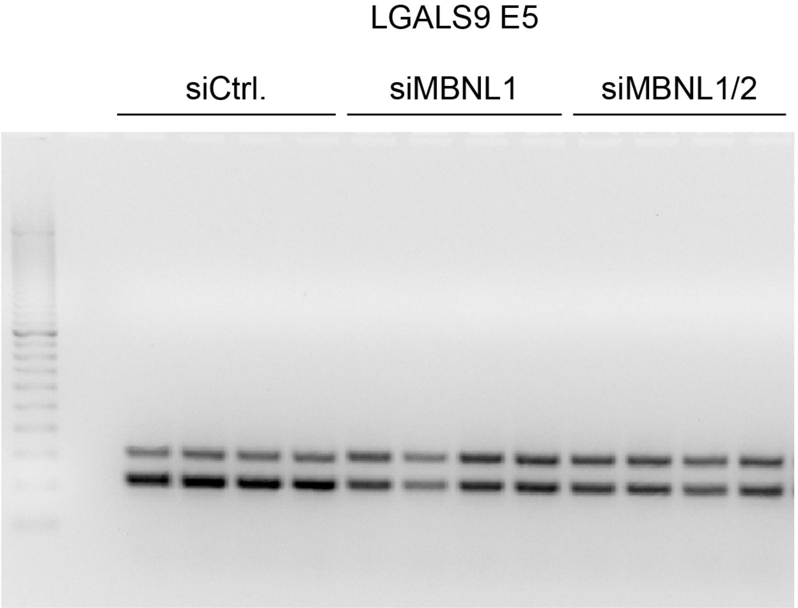

LGALS9: 318 bp (+E5); 222 bp (-E5)

Supplementary Fig. 4i

PLEKHM2 E7

siCtrl. siMBNL1 siMBNL1/2

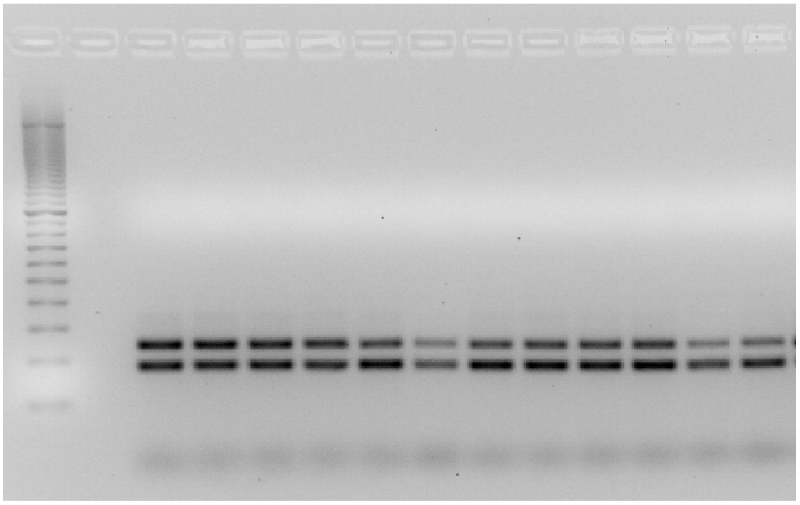

PLEKHM2: 247 bp (+E7); 187 bp (-E7)

Supplementary Fig. 4i

NUMA1 E16

siCtrl. siMBNL1 siMBNL1/2

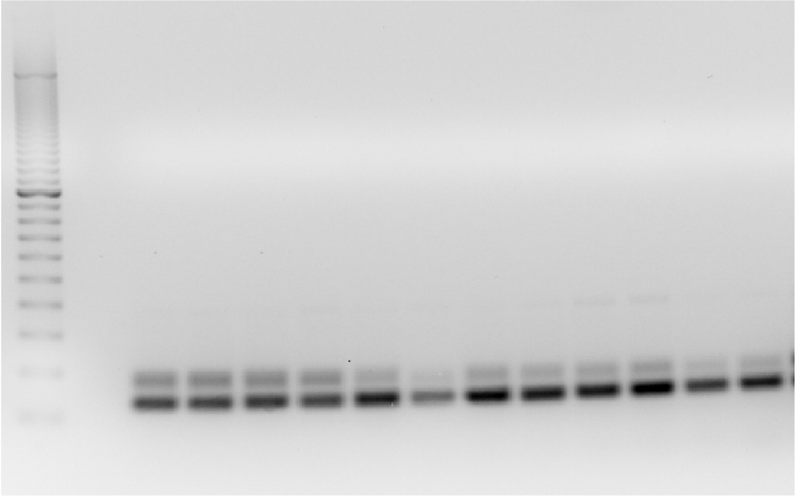

NUMA1: 184 bp (+E6); 142 bp (-E6)

Supplementary Fig. 4i

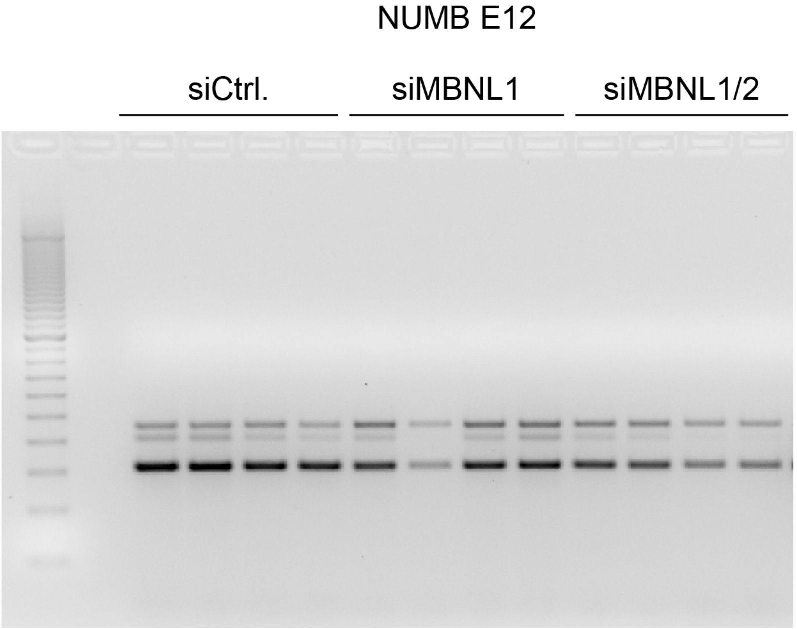

NUMB: 459 bp (+E12); 315 bp (-E12)

Supplementary Fig. 4i

SYNE1 E137

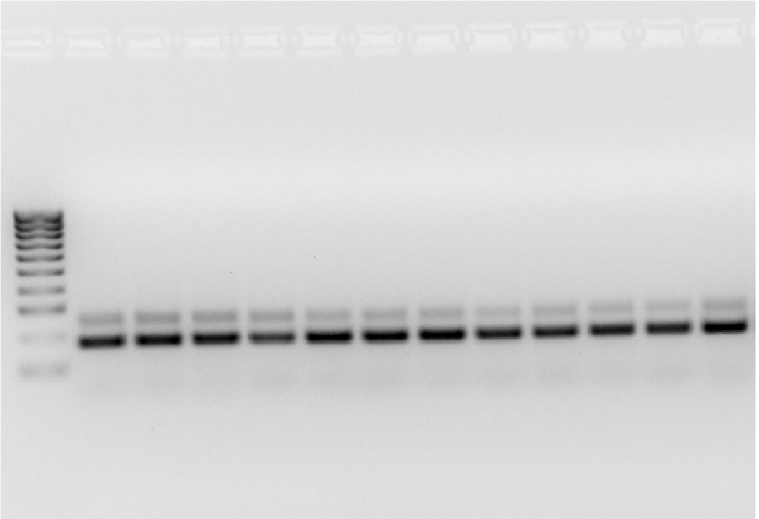

SYNE1: 245 bp (+E137); 176 bp (-E137)

Supplementary Fig. 4i

TCF7 E5

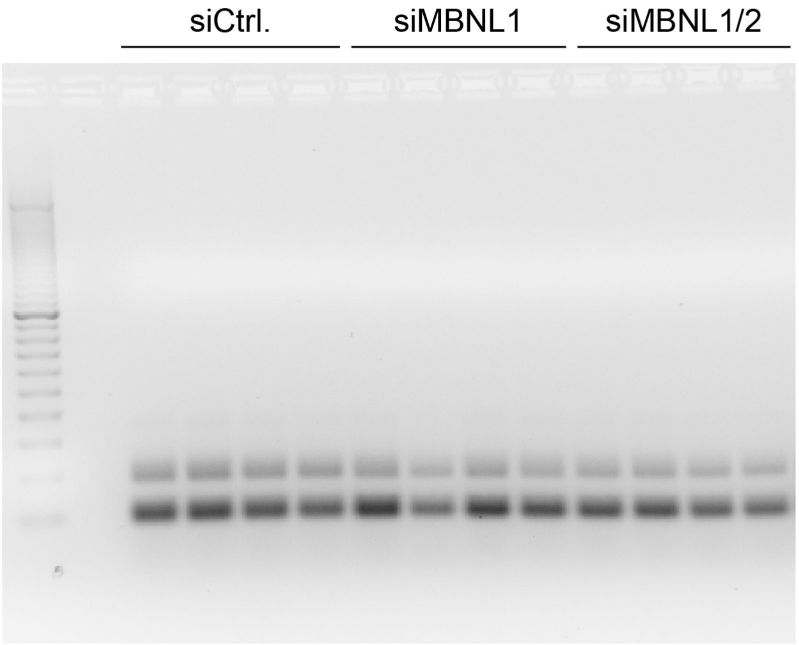

TCF7: 192 bp (+E5); 100 bp (-E5)

Supplementary Fig. 4i

TCF7 E9

siCtrl. siMBNL1 siMBNL1/2

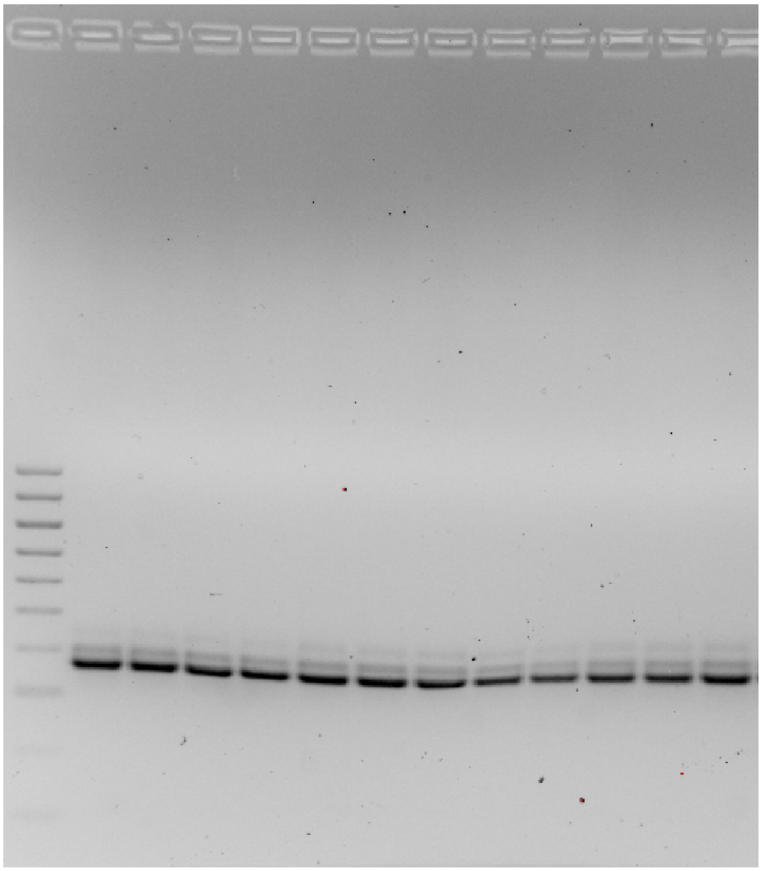

TCF7: 466 bp (+E9); 393 bp and 370 bp (-E9)

Supplementary Fig. 5a

Lef1 E6

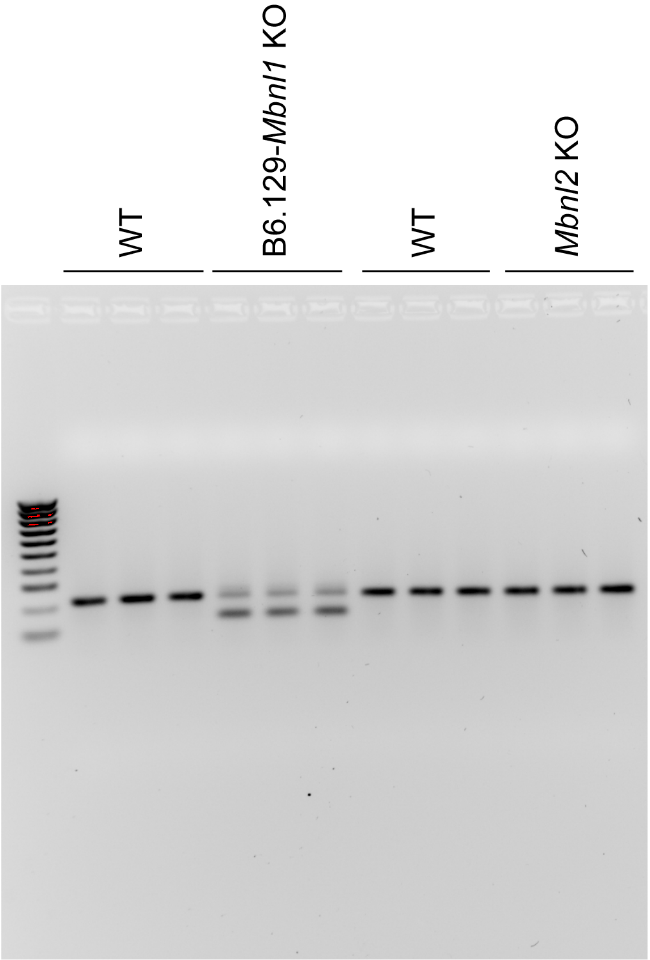

Lef1: 205 bp (+E6); 121 bp (-E6)

Supplementary Fig. 5a

Tcf7 E5

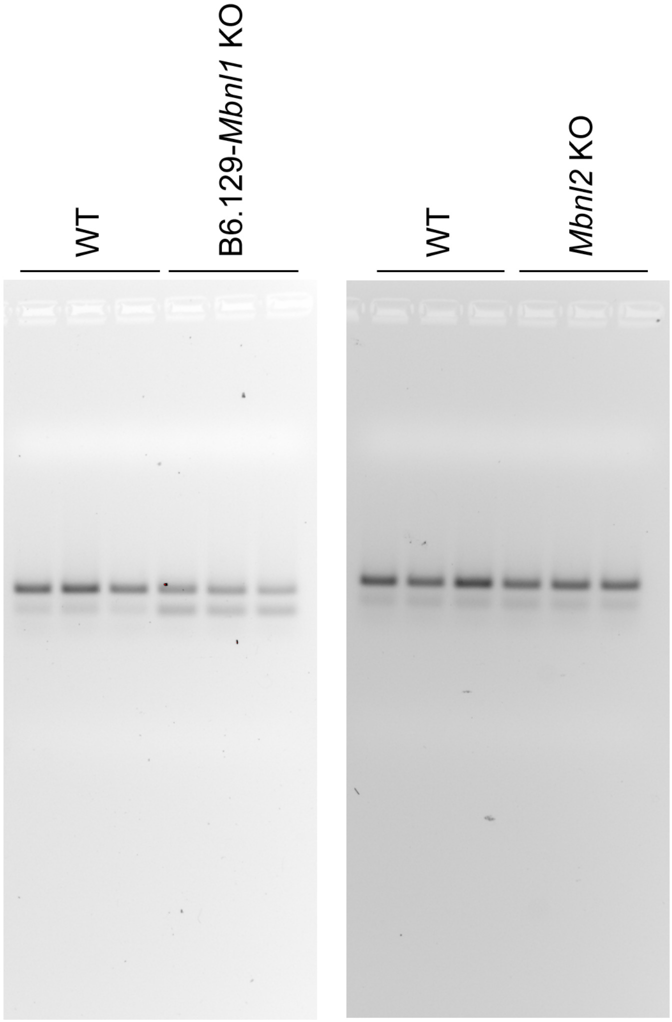

Tcf7: 206 bp (+E5); 113 bp (-E5)

Supplementary Fig. 5a

Tcf7 E10

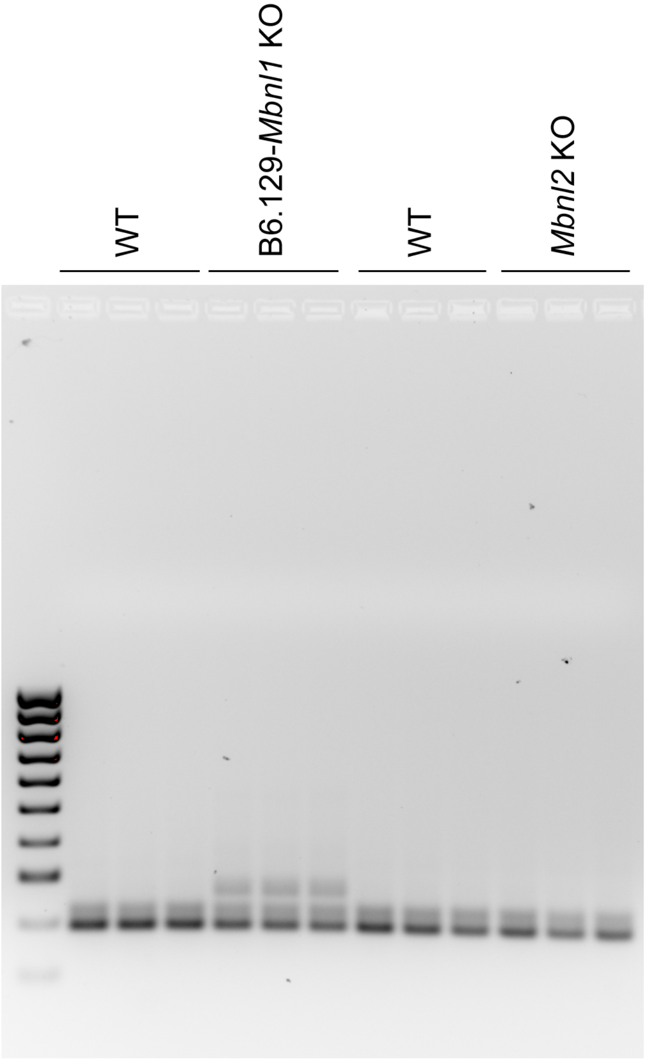

Tcf7: 277 bp and 254 bp (+E10); 204 bp and 181 bp (-E10)

Supplementary Fig. 5c

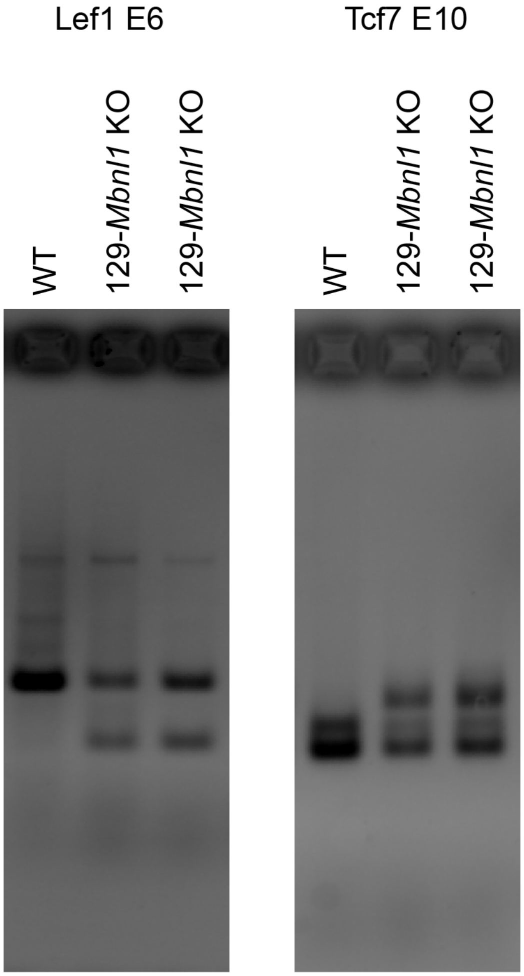

Lef1: 205 bp (+E6); 121 bp (-E6)
Tcf7: 277 bp and 254 bp (+E10); 204 bp and 181 bp (-E10)

Supplementary Fig. 5d

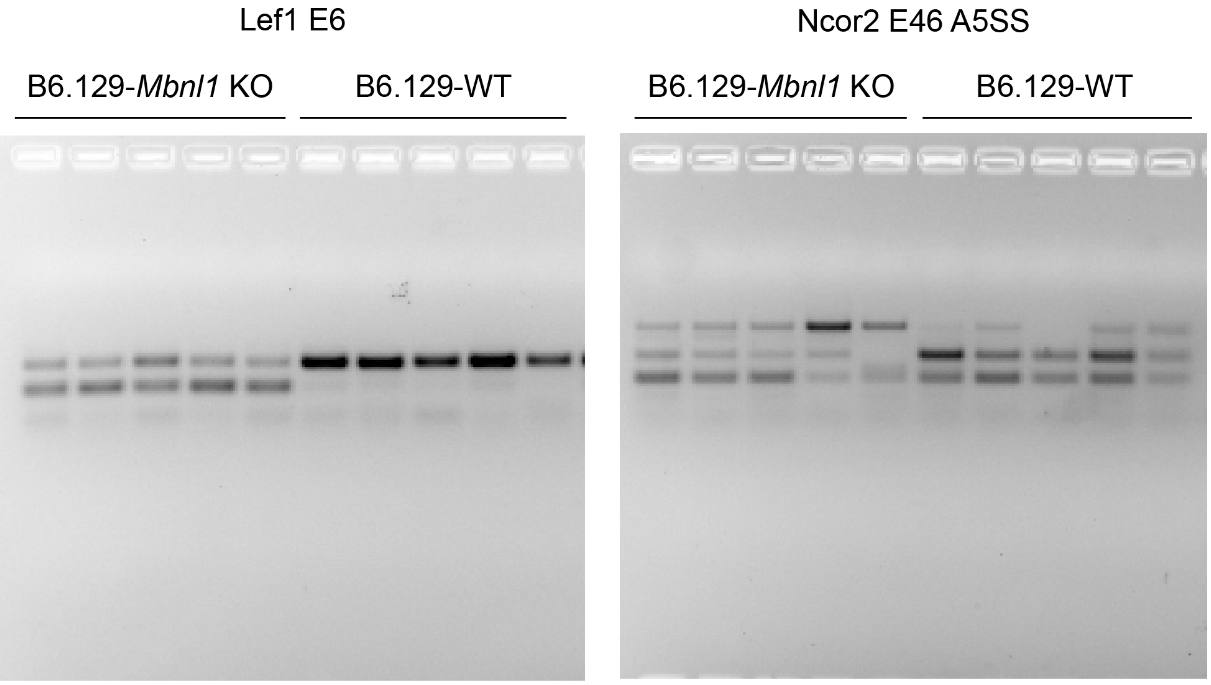

Lef1: 205 bp (+E6); 121 bp (-E6)
Ncor2: 368 bp (+E46L); 227 bp (+E46S); 140 bp (-E46)

Supplementary Fig. 5d

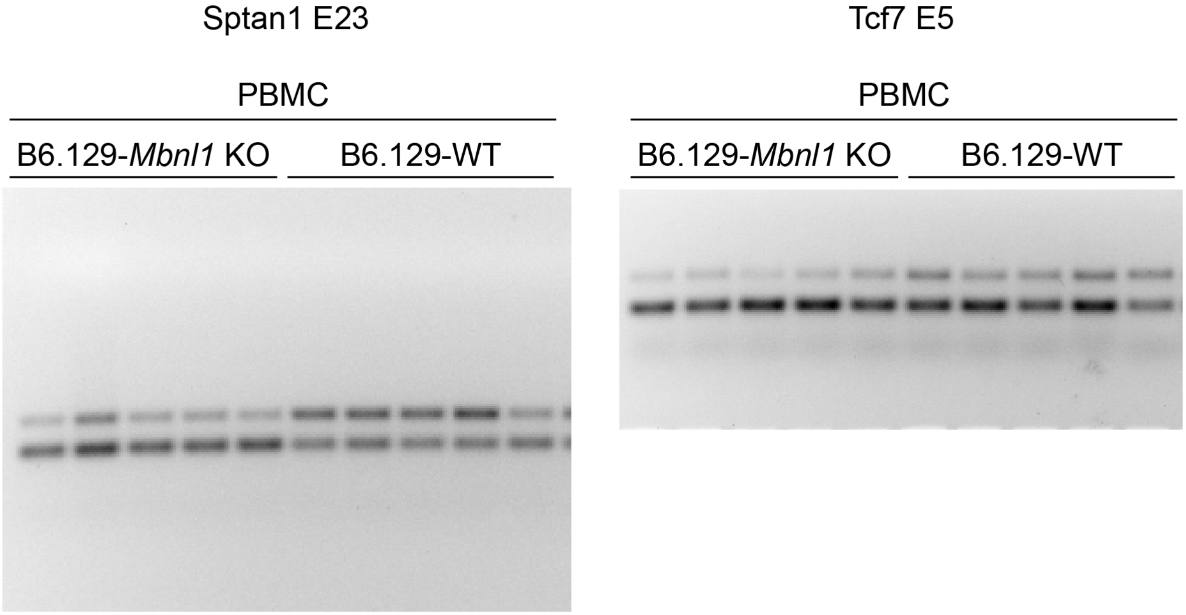

Sptan1: 185 bp (+E23); 125 bp (-E23)
Tcf7: 206 bp (+E5); 113 bp (-E5)

Supplementary Fig. 5d

Sptan1 E23

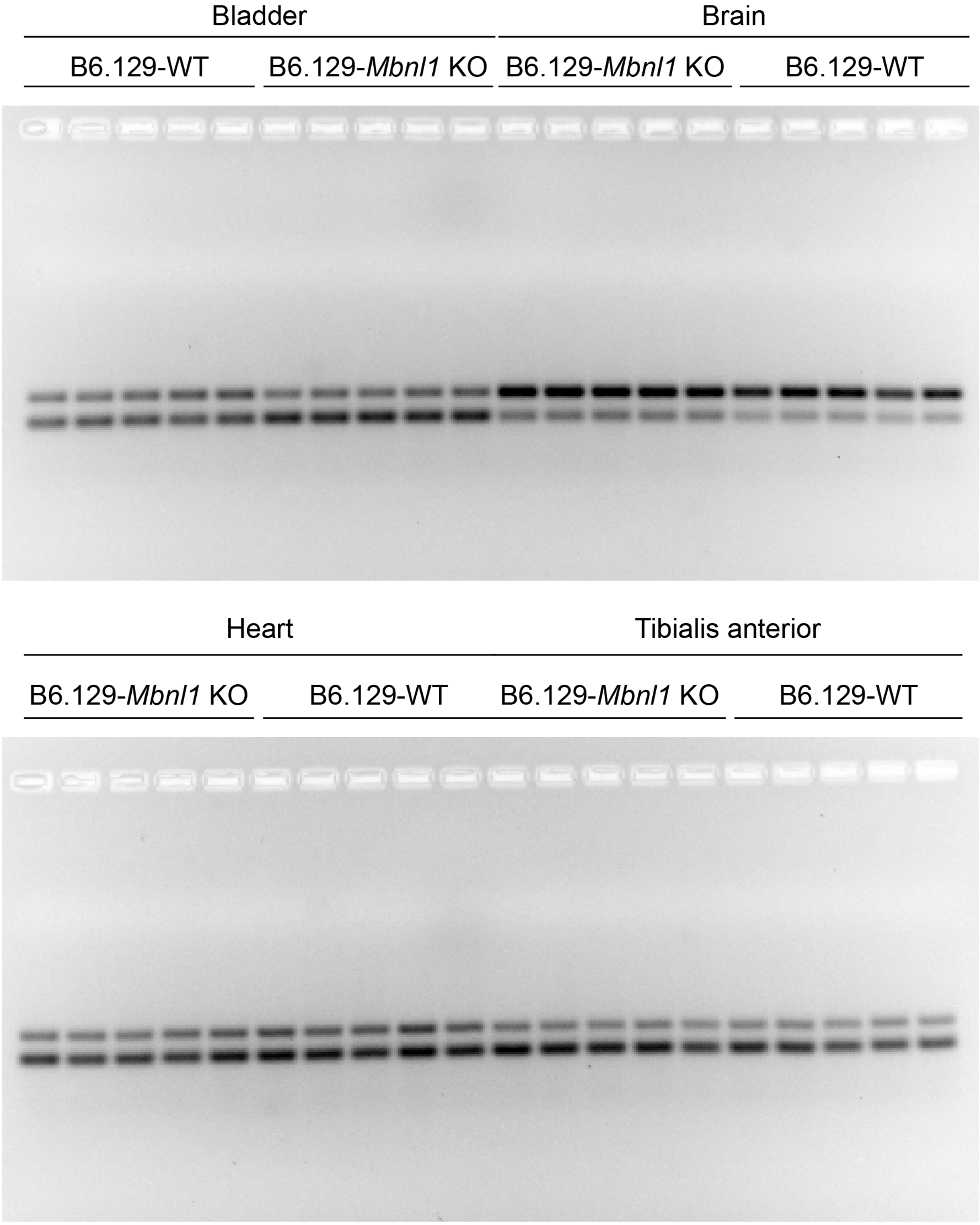

Sptan1: 185 bp (+E23); 125 bp (-E23)

Supplementary Fig. 5d

Tcf7 E5

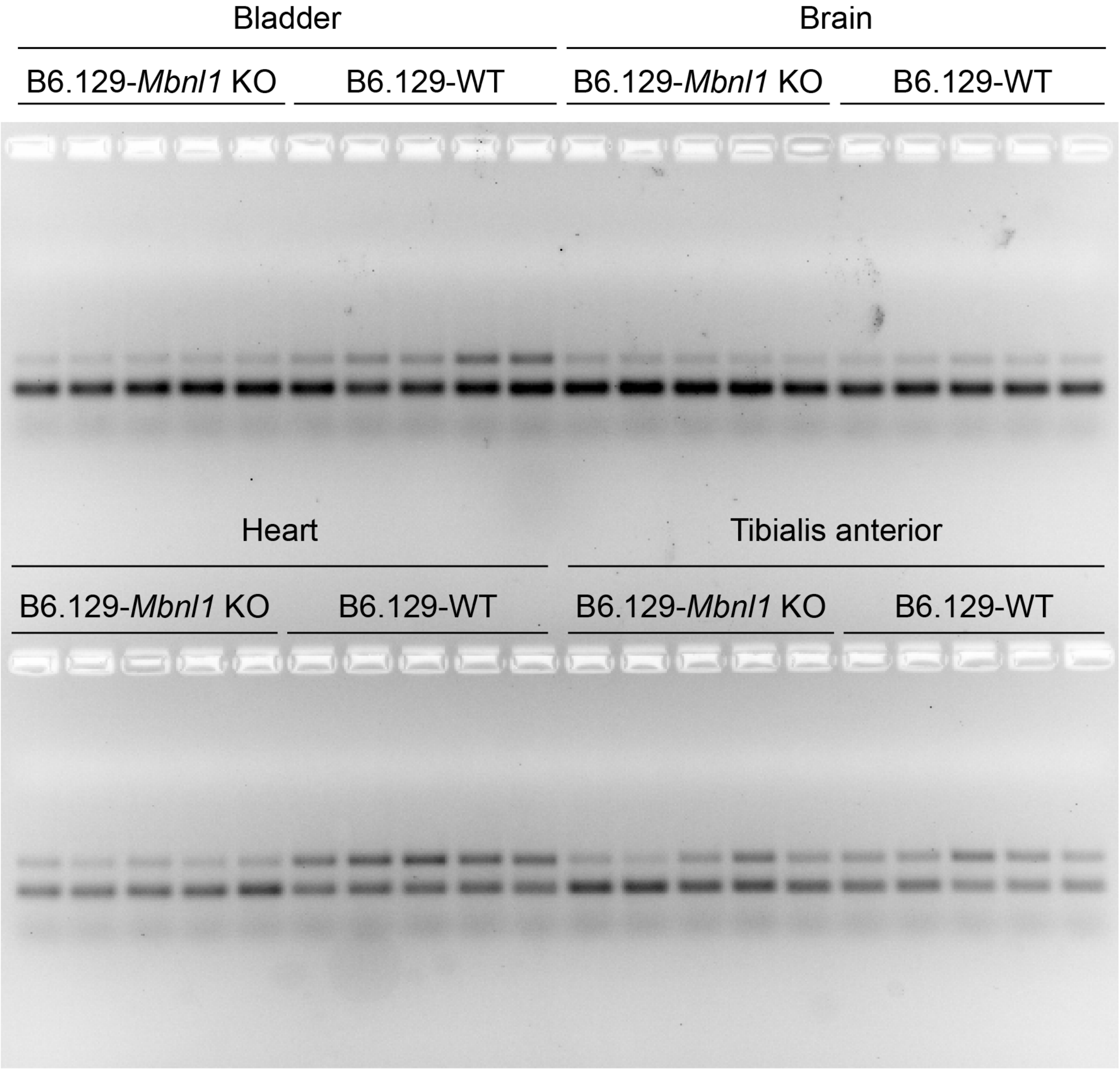

Tcf7: 206 bp (+E5); 113 bp (-E5)

Supplementary Fig. 5d

Lef1 E6

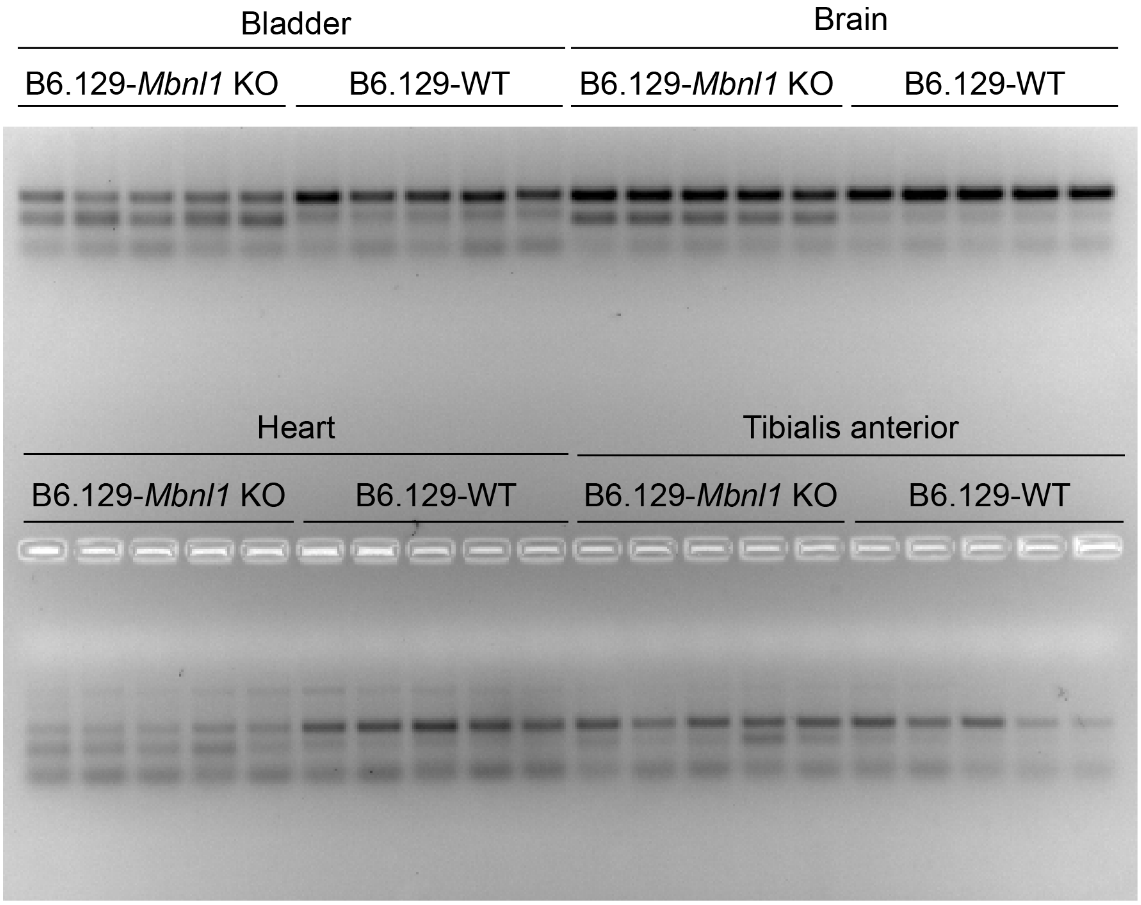

Lef1: 205 bp (+E6); 121 bp (-E6)

Supplementary Fig. 5d

Ncor2 E46

Brain | Heart

B6.129-*Mbnl1* KO | B6.129-WT | B6.129-*Mbnl1* KO | B6.129-WT

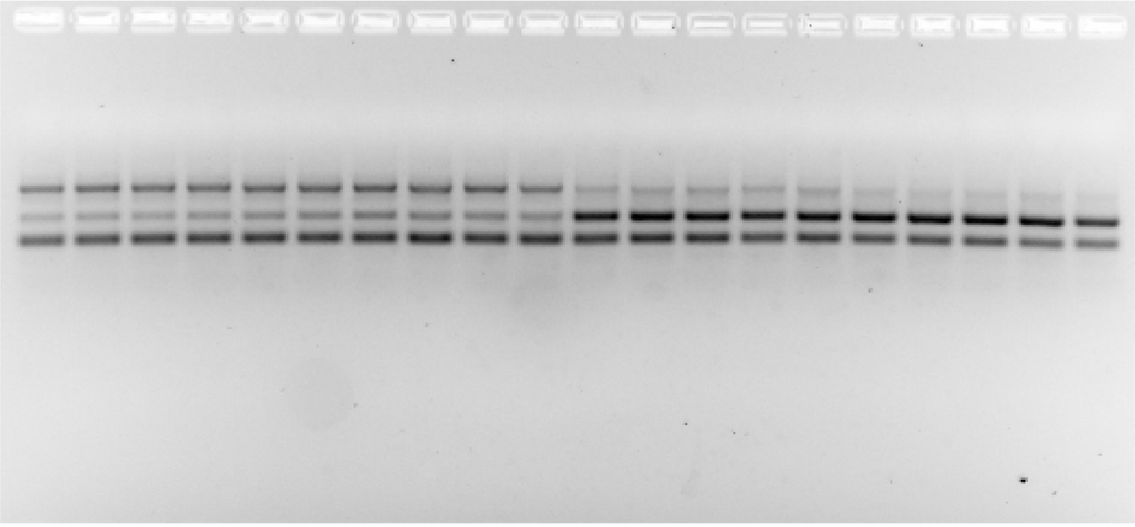

Bladder

B6.129-*Mbnl1* KO | B6.129-WT

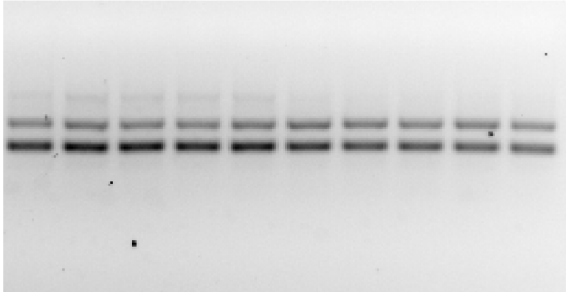

Tibialis anterior

B6.129-*Mbnl1* KO | B6.129-WT

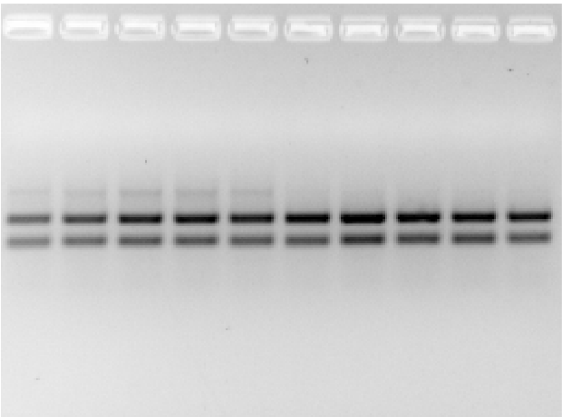

Ncor2: 368 bp (+E46L); 227 bp (+E46S); 140 bp (-E46)

Supplementary Fig. 9b

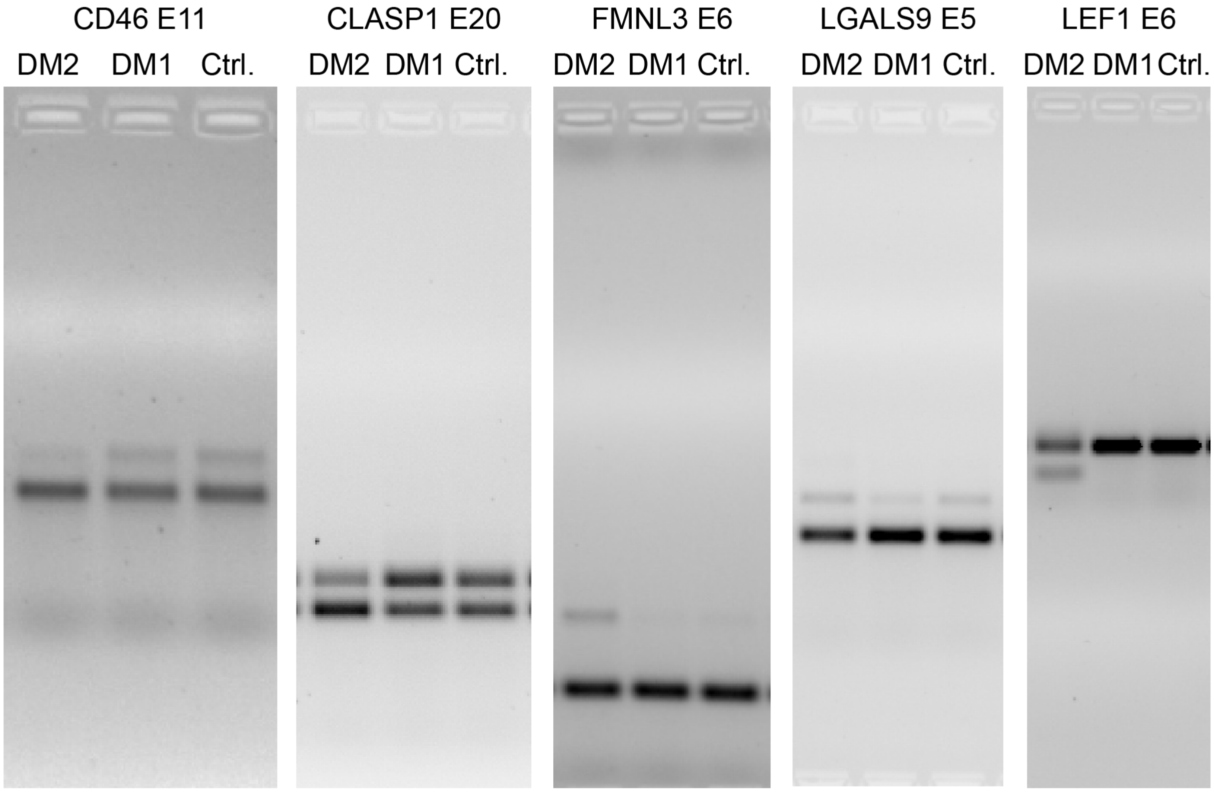

CD46: 318 bp (+E11); 225 bp (-E11)
CLASP1: 182 bp (-E20), 230 bp (+E20)
FMNL3: 303 bp (+E6); 150 bp (-E6)
LGALS9: 318 bp (+E5); 222 bp (-E5)
LEF1: 185 bp (+E6); 101 bp (-E6)

Supplementary Fig. 9b

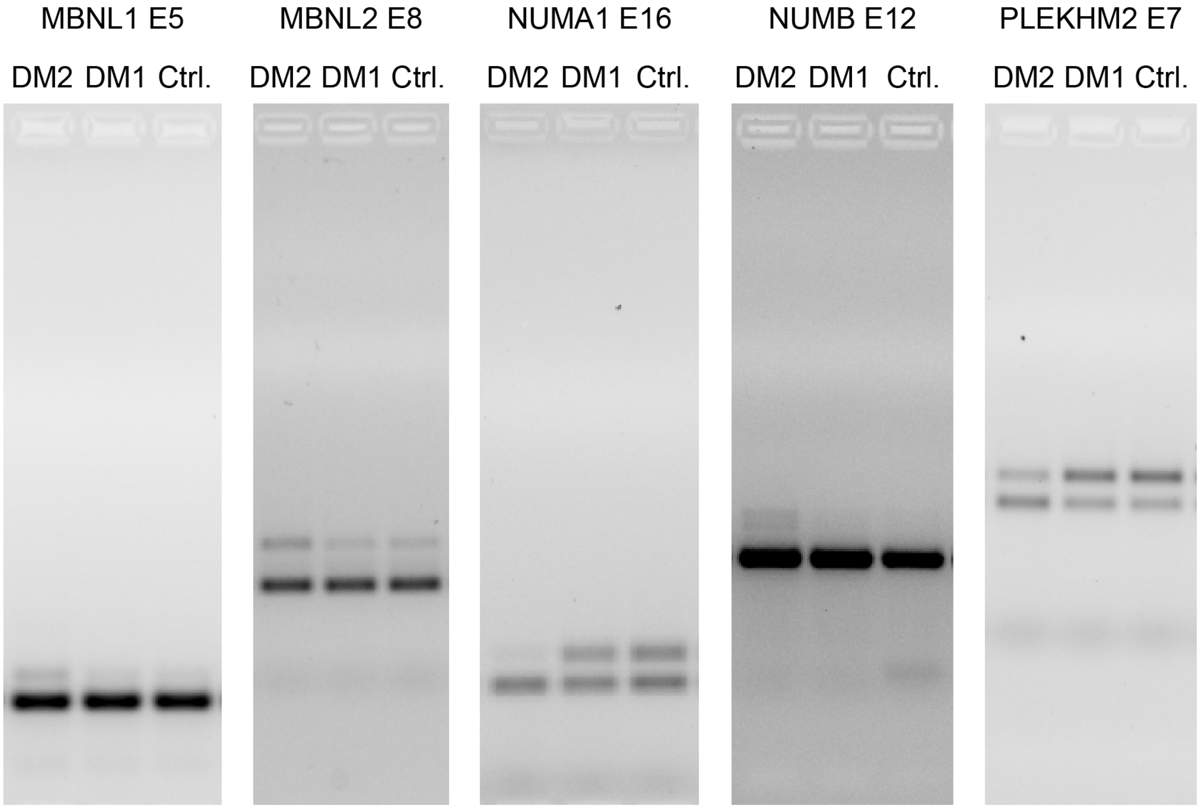

MBNL1: 270 bp (+E5); 216 bp (-E5)
MBNL2: 317 bp (+E7+E8); 281 bp (-E7+E8); 222 bp (+E7-E8); 186 bp (-E7-E8)
NUMA1: 184 bp (+E6); 142 bp (-E6)
NUMB: 459 bp (+E12); 315 bp (-E12)
PLEKHM2: 247 bp (+E7); 187 bp (-E7)

Supplementary Fig. 9b

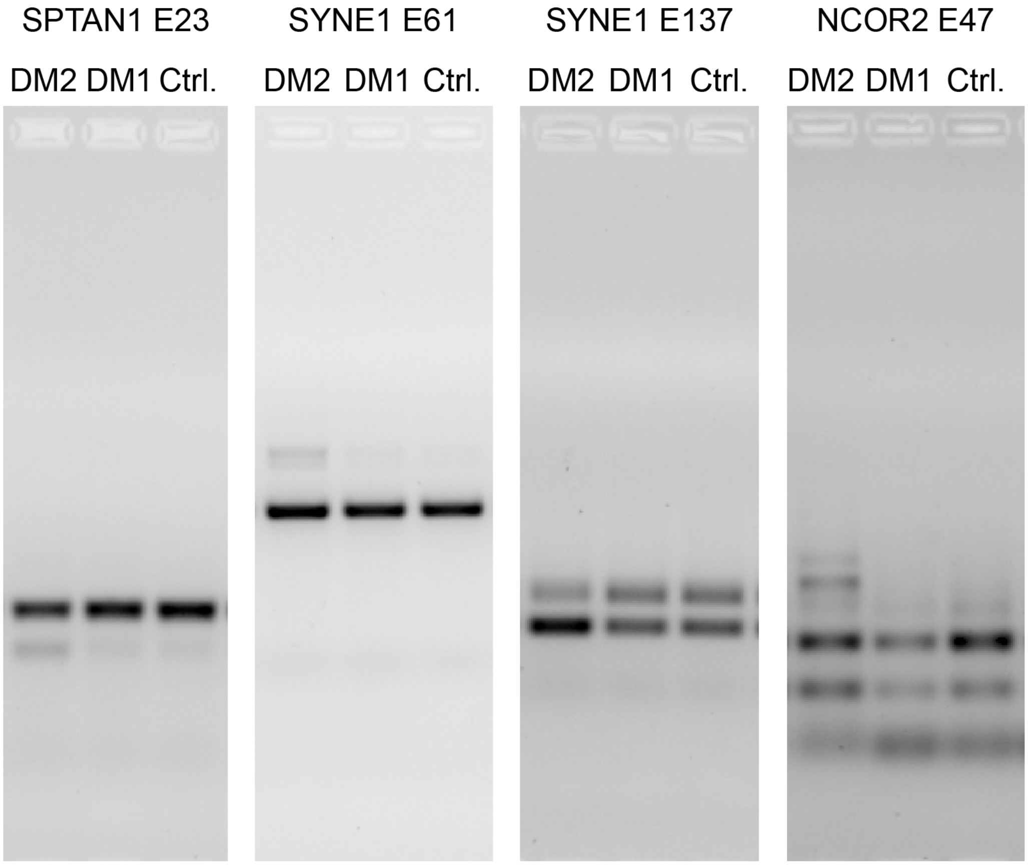

SPTAN1: 213 bp (+E23); 153 bp (-E23)
SYNE1: 403 bp (+E61); 247 bp (-E61)
SYNE1: 245 bp (+E137); 176 bp (-E137)
NCOR2: 365 bp (+E47L); 227 bp (+E47S), 149 bp (-E47)
